# Thymosin **β**4 protects against aortic aneurysm via endocytic regulation of growth factor signaling

**DOI:** 10.1172/JCI127884

**Published:** 2021-05-17

**Authors:** Sonali Munshaw, Susann Bruche, Andia N. Redpath, Alisha Jones, Jyoti Patel, Karina N. Dubé, Regent Lee, Svenja S. Hester, Rachel Davies, Giles Neal, Ashok Handa, Michael Sattler, Roman Fischer, Keith M. Channon, Nicola Smart

**Affiliations:** 1 Burdon Sanderson Cardiac Science Centre, Department of Physiology, Anatomy & Genetics, University of Oxford, Sherrington Building, Oxford, United Kingdom.; 2 Institute of Structural Biology, Helmholtz Zentrum München, Neuherberg, Munich, Germany.; 3 Biomolecular NMR and Center for Integrated Protein Science Munich at Chemistry Department, Technical University of Munich, Garching, Munich, Germany.; 4 BHF Centre of Research Excellence, Division of Cardiovascular Medicine, John Radcliffe Hospital, University of Oxford, Oxford, United Kingdom.; 5 UCL-Institute of Child Health, London, United Kingdom.; 6 Nuffield Department of Surgical Sciences, University of Oxford, Oxford, United Kingdom.; 7 Nuffield Department of Medicine, Target Discovery Institute, University of Oxford, Oxford, United Kingdom.

**Keywords:** Vascular Biology, Cardiovascular disease, Signal transduction

## Abstract

Vascular stability and tone are maintained by contractile smooth muscle cells (VSMCs). However, injury-induced growth factors stimulate a contractile-synthetic phenotypic modulation which increases susceptibility to abdominal aortic aneurysm (AAA). As a regulator of embryonic VSMC differentiation, we hypothesized that Thymosin β4 (Tβ4) may function to maintain healthy vasculature throughout postnatal life. This was supported by the identification of an interaction with low density lipoprotein receptor related protein 1 (LRP1), an endocytic regulator of platelet-derived growth factor BB (PDGF-BB) signaling and VSMC proliferation. LRP1 variants have been implicated by genome-wide association studies with risk of AAA and other arterial diseases. Tβ4-null mice displayed aortic VSMC and elastin defects that phenocopy those of LRP1 mutants, and their compromised vascular integrity predisposed them to Angiotensin II–induced aneurysm formation. Aneurysmal vessels were characterized by enhanced VSMC phenotypic modulation and augmented PDGFR-β signaling. In vitro, enhanced sensitivity to PDGF-BB upon loss of Tβ4 was associated with dysregulated endocytosis, with increased recycling and reduced lysosomal targeting of LRP1–PDGFR-β. Accordingly, the exacerbated aneurysmal phenotype in Tβ4-null mice was rescued upon treatment with the PDGFR-β antagonist Imatinib. Our study identifies Tβ4 as a key regulator of LRP1 for maintaining vascular health, and provides insights into the mechanisms of growth factor–controlled VSMC phenotypic modulation underlying aortic disease progression.

## Graphical abstract

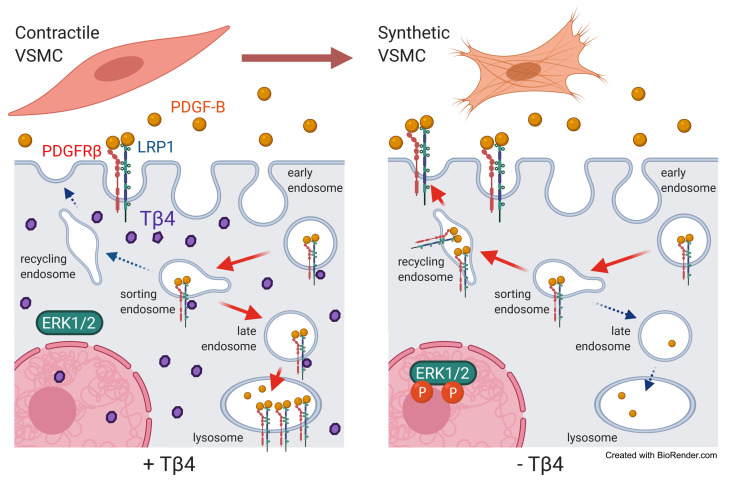

## Introduction

The integrity of the blood vessel wall is key to resisting the shear forces of flowing blood and preventing aneurysmal dilatation and rupture. Endothelial damage triggers macrophage infiltration, leading to compromised vascular stability and susceptibility to aortic aneurysm. Prevalence of aortic aneurysm is 5% among the elderly and, with no pharmacological options, treatment involves a high-risk surgical procedure. While risk of rupture is low for small, asymptomatic aneurysms, risk escalates with increasing aortic dilatation, and rupture is invariably catastrophic, with a mortality of 50%–80% (1). Genome-wide association studies (GWAS) have provided important insights into genetic predisposition for abdominal aortic aneurysm (AAA). However, a major challenge in the postgenomic era is to elucidate the molecular mechanisms through which GWAS hits influence pathogenesis (2, 3).

Vascular stability is determined by competing degenerative (smooth muscle cell [VSMC] apoptosis and elastolysis) and regenerative mechanisms (VSMC replenishment and synthesis of an elastin-rich extracellular matrix [ECM]). In their contractile state, VSMCs play an essential role in regulating vascular tone. In disease, however, growth factors such as platelet-derived growth factor BB (PDGF-BB), secreted by damaged endothelium and immune cells, induce a contractile-synthetic phenotypic switch in VSMCs to facilitate proliferation, migration, and altered ECM synthesis. Although reparative in the short term, chronic VSMC dedifferentiation leads to medial thickening and stiffness, and exacerbates inflammation and vascular instability (4). Indeed, inhibiting VSMC phenotypic transformation has been shown to attenuate progression of vascular disease (5).

Understanding mechanisms of embryonic VSMC differentiation may inform strategies for maintaining contractile phenotype, to prevent the pathological changes that underlie vascular disease. *TMSB4X*, encoding the actin monomer (G-actin) binding protein Thymosin β4 (Tβ4), is the most abundant transcript in healthy and AAA patient aorta (6), yet its endogenous roles in the adult vasculature have not been explored (7). We previously defined an essential requirement for Tβ4 in mural cell differentiation in the developing mouse embryo (8). A proportion of Tβ4-null embryos die at E12.5 with vascular hemorrhage, coincident with a reduction in VSMC coverage of the aorta (8). These findings are consistent with similar roles for Tβ4 in smooth muscle differentiation in the coronary (9) and yolk sac vasculature (10). Despite the severe embryonic defects, the majority of Tβ4-KO mice survive to adulthood (8). To determine whether Tβ4 is required to maintain vascular integrity postnatally, we sought to investigate the phenotype of adult vessels. Tβ4-null aortas were significantly dilated with highly disorganized and irregular VSMC morphology accompanied by aberrant elastin deposition, defects that are observed in patients with AAA (11) and associated with severely compromised vascular stability. In keeping with this, we confirmed predisposition of global and VSMC-specific Tβ4 loss-of-function mice to disease in an experimental model of aortic aneurysm (1 mg/kg/d Angiotensin II). Aortic dilatation in mutant mice was underpinned by enhanced contractile-synthetic VSMC switching and dysregulated PDGFR-β signaling.

Seeking insight into the underlying mechanisms, we identified an interaction between Tβ4 and low density lipoprotein receptor related protein 1 (LRP1), which functions in VSMC development and protection by regulating growth factor signaling (12, 13) and ECM remodeling (14, 15). The interaction was confirmed by nuclear magnetic resonance (NMR) spectroscopy and mapped to the LRP1 cytoplasmic tail. Notably, Tβ4 binds residues near the NPxY motifs, which are associated with signal transduction and receptor recycling. LRP1 variants have been identified by GWAS as major risk loci for AAA (16), carotid (17), and coronary artery disease (18). LRP1 is involved in vascular remodeling, inflammation, differentiation, and cell migration (19), roles shared with Tβ4, and has been shown in animal studies to protect against aneurysm and atherosclerosis (12, 20). Among its many roles, LRP1 functions as an endocytic coreceptor for PDGFR-β to both potentiate and attenuate downstream signaling activity (21). From early endosomes, the LRP1–PDGFR-β complex may be recycled to the cell membrane or targeted for lysosomal degradation; such trafficking critically determines sensitivity to PDGF isoforms and, thereby, cellular responses. We report the hyperactivation of LRP1–PDGFR-β signaling in Tβ4-null aortic VSMCs and find that augmented sensitivity in vitro correlates with increased recycling of LRP1–PDGFR-β complexes to the cell surface, concomitant with reduced lysosomal targeting. We demonstrate that, following activation, PDGFR-β associates with Filamin A, the actin-crosslinking protein responsible for endocytic sorting and rapid recycling of signaling receptors. Significantly enhanced Filamin A levels in Tβ4 knockdown VSMCs may underlie the increased cell surface receptor levels and sensitivity of the cells to PDGF-BB. That dysregulated PDGFR-β signaling promotes aneurysm formation in Tβ4-KO mice was confirmed upon rescue of the mutant phenotype to control level with Imatinib, a PDGFR-β antagonist. Taken together, these findings suggest a requirement for Tβ4 in the normal attenuation of VSMC PDGFR-β signaling, via lysosomal targeting of the receptor, to maintain contractile VSMC phenotype and vascular stability. Our study defines a novel mechanism by which Tβ4 controls LRP1-mediated VSMC responses to protect against vascular disease. Given that LRP1 has been implicated in multiple GWAS as a key regulator of vascular protection, understanding the molecular mechanisms through which it preserves vascular health may enable the development of novel therapies for modulation of VSMC phenotypic switching and disease progression.

## Results

### A role for Tβ4 in maintenance of adult vascular stability.

**Figure 1:**
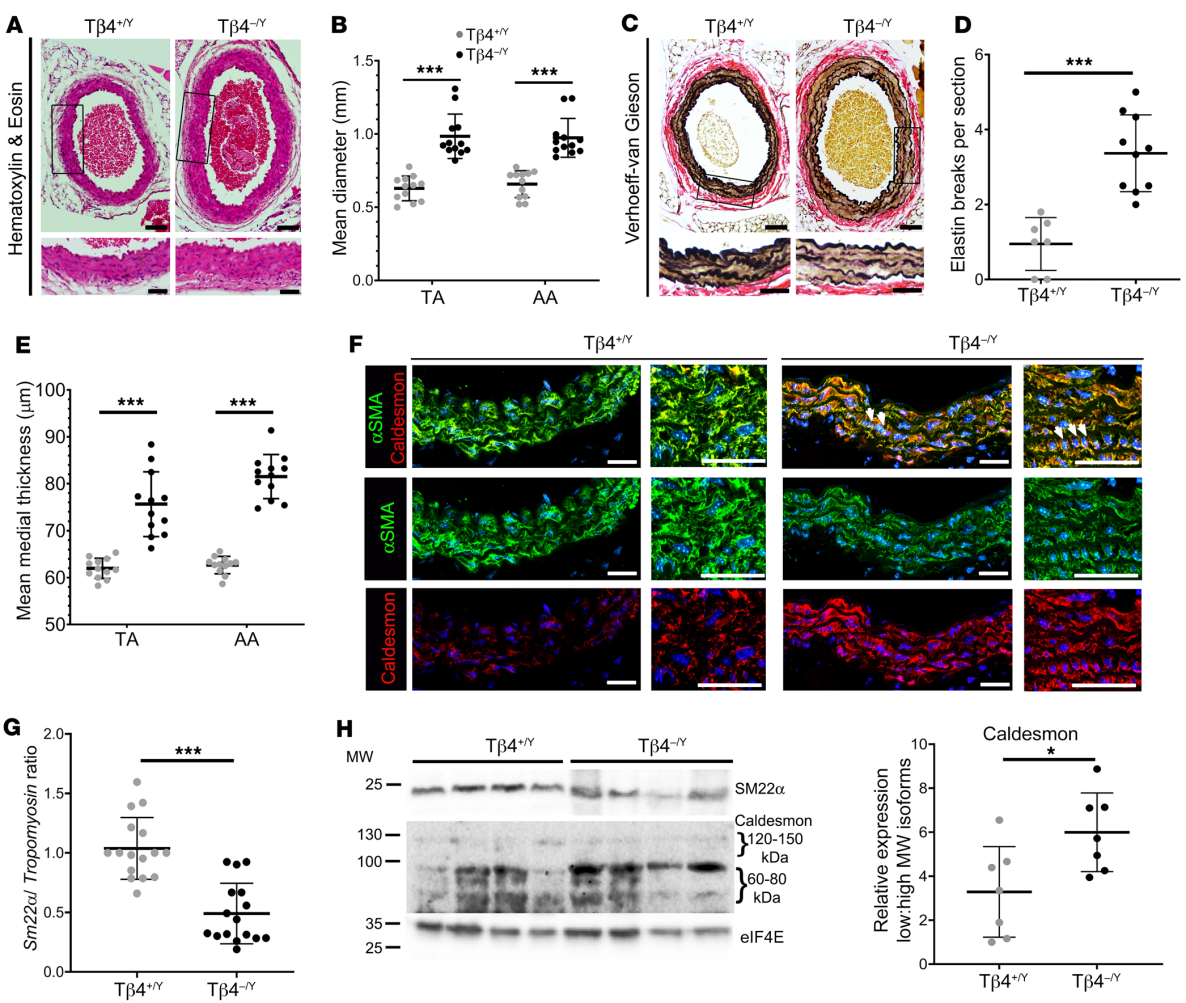
Tβ4-null mice display baseline aortic defects in adulthood. (**A**) Hematoxylin and eosin staining to assess morphology and aortic dilatation, quantified in **B**. Verhoeff–van Gieson staining (**C**) to assess elastin integrity, quantified both by number of breaks per section (**D**) and by an elastin damage score (Supplemental Figure 1). Medial thickness is quantified (**E**). Mean of 6 sections per aorta (**B**, **D**, and **E**). Contractile/synthetic smooth muscle markers were assessed by immunofluorescence (**F**, quantified in Supplemental Figure 2C), qRT-PCR (**G**), and Western blotting (**H**, representative of *n* = 7; SM22α quantification in Supplemental Figure 2D). Altered contractile/synthetic marker profile was accompanied by altered morphology, with dense clusters of cells of VSMCs with a cuboidal, rather than elongated, appearance (white arrowheads). Data are mean ± SD, with each data point representing an individual animal (12- to 16-week-old male mice). Significant differences were calculated using Mann-Whitney nonparametric tests (**B**, **D**, **E**, and **H**) or a 2-tailed unpaired Student’s *t* test (**G**), with Holm-Sidak correction for multiple comparisons (**B** and **E**). *P ≤ 0.05; ***P ≤ 0.001 for Tβ4^+/Y^ vs Tβ4^–/Y^. Scale bars: **A** and **C**: 100 μm (whole aorta); 50 μm (enlarged); **F**: all 50 μm.

Although vascular defects cause lethality in a proportion of *Tmsb4x*/Tβ4 null embryos (36% of Tβ4^–/Y^ males; 16% of Tβ4^–/–^ females) (8), most survive to adulthood. This prompted us to investigate whether vessel structure and function were entirely normal in viable adults. Due to the higher mortality in male embryos, we focused our studies on male mice. Compared with control Tβ4^+/Y^ aortas, Tβ4^–/Y^ aortas of 12- to 16-week-old mice were significantly dilated (mean 1.5-fold, determined histologically from both abdominal [AA] and thoracic [TA] sections; Figure 1, A and B). Moreover, aberrant elastin lamellar integrity suggested severely compromised vascular stability (3.6-fold more elastin breaks per section; Figure 1, C and D) and higher elastin damage score, according to a previously defined scoring system (22), exemplified and quantified in Supplemental Figure 1, A and B; supplemental material available online with this article; https://doi.org/10.1172/JCI127884DS1). Tβ4^–/Y^ aortas displayed medial thickening (Figure 1, A and E), and a 1.4-fold elevation of systemic vascular compliance was revealed by combined MRI and arterial blood pressure measurements (stroke volume/pulse pressure; Supplemental Figure 2A). Masson’s trichrome staining revealed limited aortic fibrosis (Supplemental Figure 2B). As platelets are a major source of factors, such as PDGF-BB, which promote medial layer defects, we excluded differences in the density of platelets (CD41) and activated platelets (P Selectin/PECAM-1) associating with the intimal layer (Supplemental Figure 3A) and confirmed comparable aortic *Pdgfb* expression between genotypes (Supplemental Figure 3B). Due to the requirement for Tβ4 in VSMC differentiation (8, 9, 23), we assessed the phenotype of medial layer VSMCs by measuring expression of established contractile (αSMA and SM22α) and so-called synthetic markers (*Tropomysin* and low-molecular-weight isoforms of Caldesmon) (24) by quantitative reverse transcription real-time PCR (qRT-PCR), immunofluorescence, and Western blotting (Figure 1, F–H; further quantification in Supplemental Figure 2, C and D; all full, uncut gels are published as online supplemental material). Significant reductions in the proportions of contractile/synthetic protein expression confirmed an overall shift toward more synthetic VSMCs in Tβ4^–/Y^ aortas, which was consistent with the disorganized VSMC morphology observed (Figure 1F). While Tβ4^+/Y^ aortas contained regularly aligned, elongated VSMCs, Tβ4^–/Y^ aortas frequently contained clusters of small, densely packed cuboidal cells. Western blotting confirmed that the overall increase in Caldesmon levels (Figure 1F; quantified in Supplemental Figure 2C) results from a proportionally larger increase in the lower molecular weight isoforms associated with the synthetic phenotype (25, 26) (Figure 1H). Collectively, these data suggest that Tβ4^–/Y^ VSMCs are less differentiated and more synthetic in phenotype than Tβ4^+/Y^ VSMCs and that these defects may compromise vascular stability.

**Figure 2:**
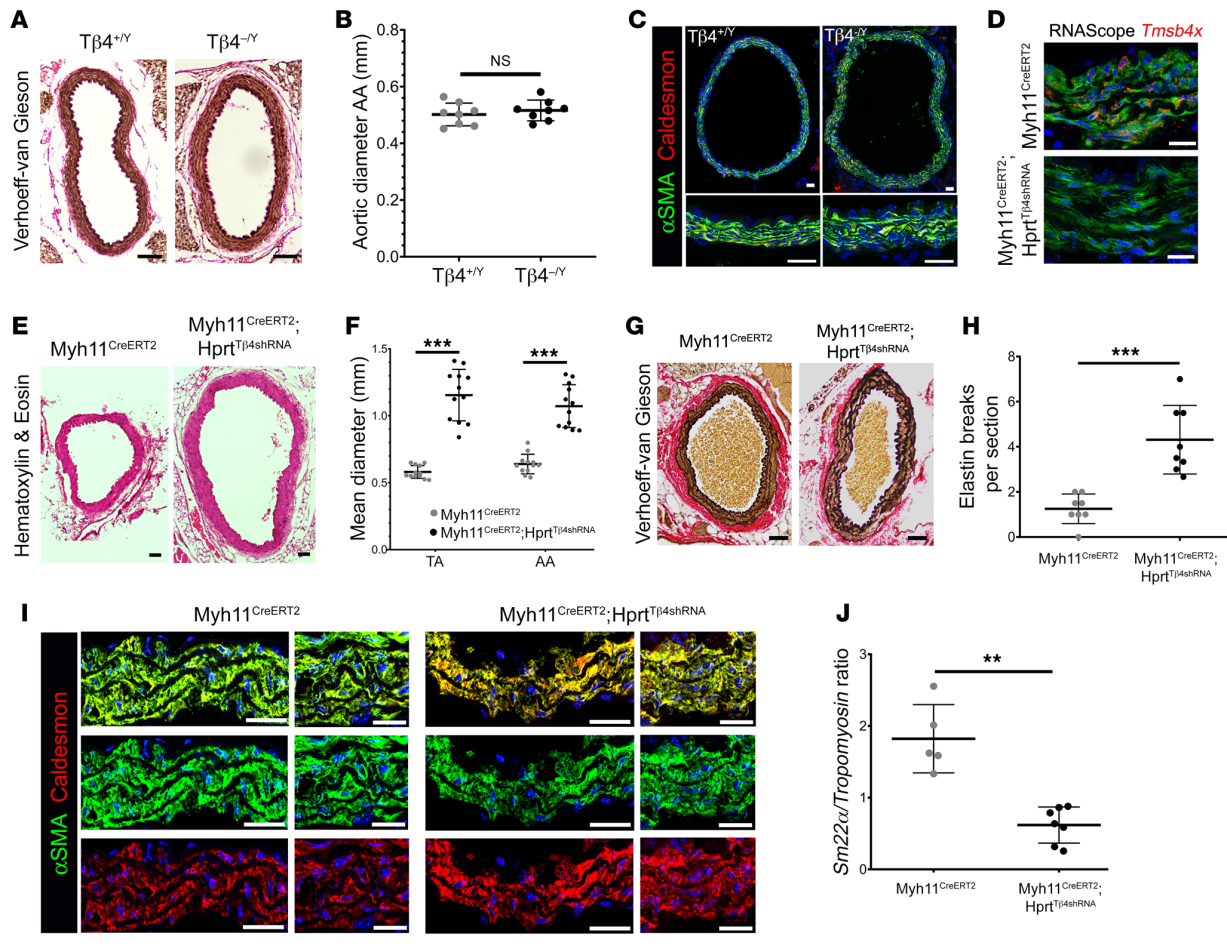
A postnatal, smooth muscle cell-autonomous requirement for Tβ4 for maintenance of healthy aorta. Verhoeff–van Gieson staining (**A**) to visualize elastin integrity, structure, diameter (**B**), and medial thickness (Supplemental Figure 4A) of aortas in P7 male mice. Immunofluorescence to assess smooth muscle phenotype (**C**). *Tmsb4x* was deleted from medial VSMCs of 3-week-old Myh11^CreERT2^ Hprt^Tβ4shRNA^ knockdown mice; RNAScope for *Tmsb4x* mRNA, compared with Myh11^CreERT2^ Hprt^+/+^ control mice (**D**), quantified in Supplemental Figure 4B. Hematoxylin and eosin staining (**E**) assessed aortic dilatation and medial thickness in 12-week-old mice, quantified in **F** and Supplemental Figure 4C, respectively. Verhoeff–van Gieson staining (**G**) assessed elastin integrity, quantified both by number of breaks per section (**H**) and by elastin damage score (Supplemental Figure 1). Ratio of contractile/synthetic VSMC markers in Myh11^CreERT2^ Hprt^Tβ4shRNA^, compared with Myh11^CreERT2^ Hprt^+/+^ aortas, both at the protein (**I**; quantified in Supplemental Figure 4E) and mRNA level (qRT-PCR; **J**). Data are mean ± SD, with each data point representing an individual animal. Significance was calculated using a Mann-Whitney nonparametric test (**B** and **J**) or 2-tailed unpaired Student’s *t* tests (**F** and **H** with Holm-Sidak correction for multiple comparisons in **F**). **P ≤ 0.01; ***P ≤ 0.001. Scale bars: **A**, **E**, and **G**: 100 μm; **C**: 50 μm; **D**: 20 μm; **I**: 50 μm (low), 20 μm (high).

Perturbations in embryonic vascular development can predispose to aortic disease later in life (27) and, thus, congenital VSMC differentiation defects in Tβ4-null mice may persist to adulthood and underlie medial degeneration. However, as previously reported, surviving embryos appeared to adequately compensate by normalizing growth factor signaling to develop an overtly normal vasculature by E14.5 (8). We therefore examined aortas of P7 male Tβ4^–/Y^ mice and compared them with those of littermate Tβ4^+/Y^ controls. Histological analysis of elastin integrity, aortic diameter, medial thickness, VSMC morphology, and phenotypic marker expression confirmed that Tβ4^–/Y^ mice were indistinguishable from controls in the immediate postnatal period (Figure 2, A–C, and Supplemental Figure 4A), confirming adequate compensation during development. This raises the intriguing possibility that Tβ4 may be required throughout life to maintain vascular health and prevent defects in adulthood. To test this hypothesis, and to simultaneously determine if any postnatal requirement for Tβ4 is VSMC-autonomous rather than paracrine, given the known roles in endothelial (7, 8) and immune cells (28, 29), we induced VSMC-specific loss of Tβ4 in postnatal mice. This was achieved by crossing a conditional (floxed) Tβ4 shRNA-expressing line (Hprt^Tβ4shRNA^; described in refs. 8 and 9) with Myh11^CreERT2^ (30) and administering 3 doses of tamoxifen (80 mg/kg) to 3-week-old male mice before examining their aortas at 12 weeks of age. Loss of *Tmsb4x* mRNA from aortic VSMCs was determined by RNA in situ hybridization (RNAScope; Figure 2D; quantification Supplemental Figure 4B). Compared with tamoxifen-dosed Myh11^CreERT2^ Hprt^+/+^ control mice, Myh11^CreERT2^ Hprt^Tβ4shRNA^ knockdown mice displayed a 1.8-fold aortic dilatation (Figure 2, E and F) and increased medial thickening (Supplemental Figure 4C). A substantial disruption of elastin lamellae was observed (3.4-fold increase in number of breaks per section, Figure 2, G and H) and minimal fibrosis (Supplemental Figure 4D), while irregular VSMC morphology accompanied a shift toward expression of synthetic markers (Figure 2, I and J; quantification Supplemental Figure 4E). The recapitulation of the global knockout phenotype suggests that reduction in Tβ4 levels over 8 weeks of postnatal life impairs vascular stability and defines a VSMC-autonomous, protective role for Tβ4 in adult vessel homeostasis.

### Tβ4 interacts with the vasculoprotective endocytic receptor LRP1.

To gain insight into the possible mechanisms by which Tβ4 maintains vascular health, we performed a yeast 2-hybrid screen to identify putative binding partners from an E11.5 murine embryonic library (Supplemental Table 1). A leading candidate was LRP1, associated in human (16–18) and animal studies (12, 19) with protection against AAA and atherosclerosis. The clone identified among the prey plasmids after stringent selection contained most of the intracellular domain (ICD) sequence of *Lrp1*.

**Figure 3:**
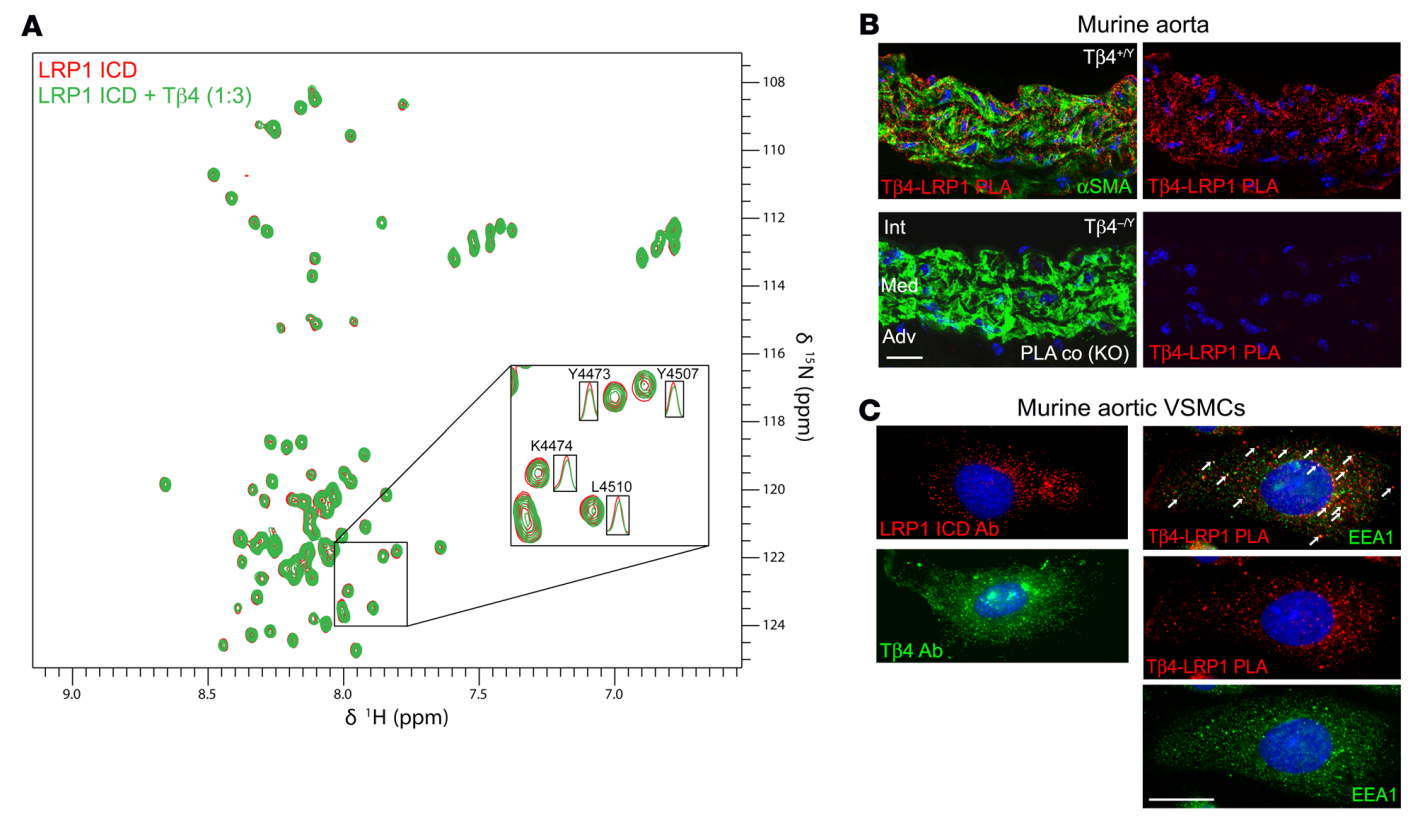
Tβ4 interacts with LRP1 in the endocytic compartment of aortic smooth muscle cells. HMQC NMR spectra of ^15^N-labeled ICD of LRP1 and 3:1 Tβ4 (**A**). Proximity ligation assay demonstrated less than 40 nm proximity, suggesting an interaction of Tβ4 with LRP1 in murine aorta (**B**; representative image of *n* = 3). Immunofluorescence for LRP1 and Tβ4 revealed localization to punctate vesicular structures (**C**) in murine primary aortic VSMCs. PLA for Tβ4 and LRP1, with some signals localizing to early endosomes, labelled with EEA1 (**C**). Scale bars: **B**: 20 μm; **C**: 10 μm.

While Tβ4 binds with high affinity to monomeric actin (31), most of its characterized interactions (e.g., with PINCH, ILK, and Stabilin-2) are described as fuzzy, in that they are weak and transient yet specific interactions with intrinsically disordered proteins that lack well-defined 3D structure (32). To validate and gain insight into the nature of the Tβ4-LRP1 interaction, we used NMR spectroscopy. We first evaluated the structural conformation of the LRP1 ICD, purified by affinity chromatography after expression in *E*. *coli*. ^1^H-^15^N SOFAST Heteronuclear Multiple Quantum Coherence (HMQC) (33) spectra revealed minimal dispersion of amide resonances (Supplemental Figure 5), indicating that LRP1 ICD is intrinsically disordered, as previously reported (34). Titration of synthetic Tβ4 into ^15^N-labeled LRP1 ICD showed minor spectral changes and some line broadening of amide resonances, indicating a weak binding interaction between the 2 proteins. Notably, at a 3:1 ratio of Tβ4 to LRP1 ICD, line broadening was most apparent in amide resonances corresponding to residues of and adjacent to the 2 NPxY motifs (Figure 3A), which include the tyrosine phosphorylation sites Y4473 and Y4507. This suggests that the interaction between Tβ4 and LRP1 ICD may be focused around the NPxY motifs, associated with signal transduction and receptor recycling.

**Figure 4:**
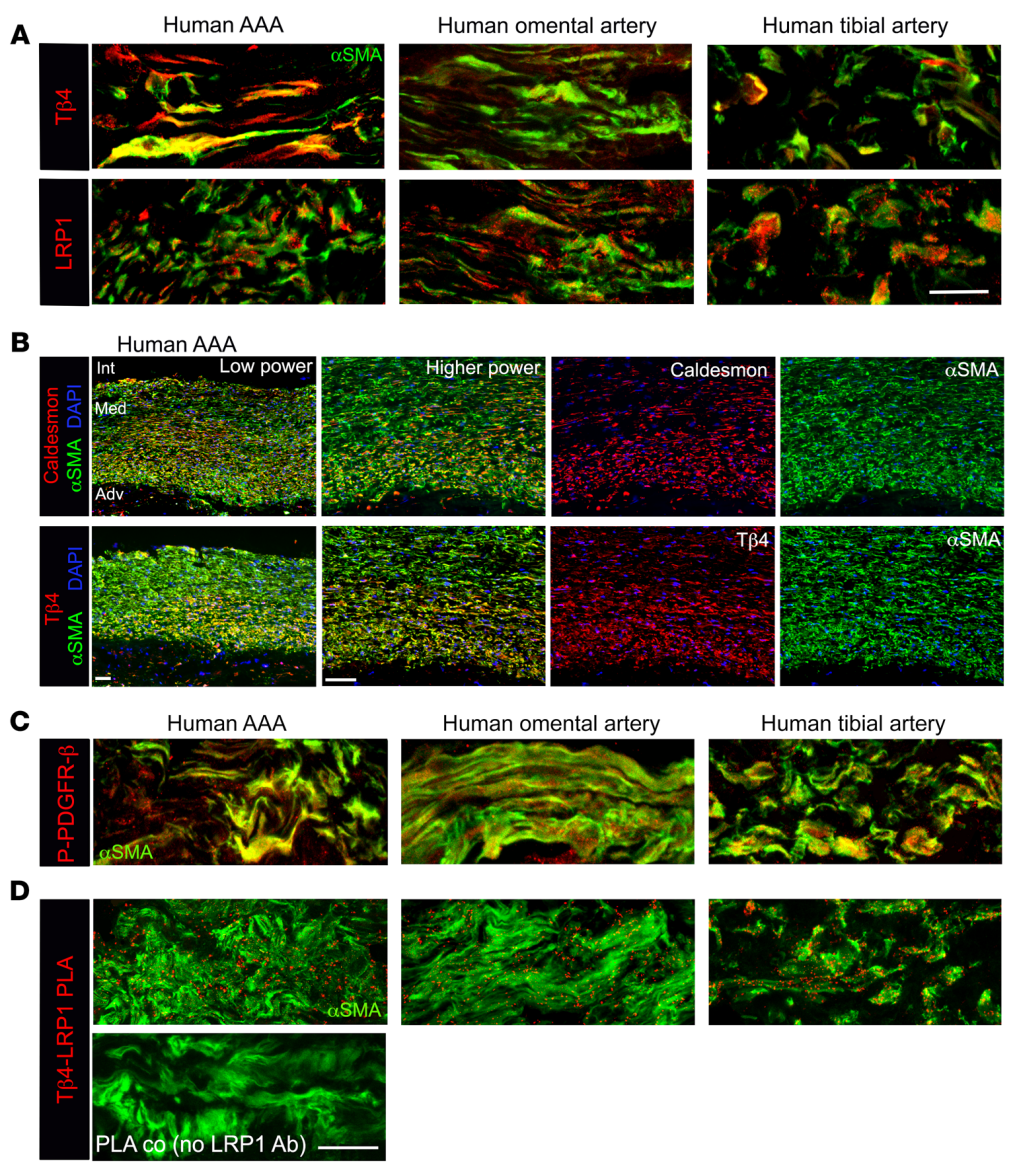
Tβ4 interacts with LRP1 in human arterial smooth muscle cells. (**A**) Immunofluorescence, with quantification shown in Supplemental Figure 6, to assess Tβ4 and LRP1 expression in human aorta from AAA patients and matched omental artery from the same patients (*n* = 10); these readouts were additionally measured in human tibial arteries (*n* = 4). (**B**) Qualitative correlation by immunofluorescence of Tβ4 with caldesmon levels in adjacent AAA sections; Tβ4 levels did not appear to correlate with αSMA in the same sections. The extent of activated (phosphorylated) PDGFR-β (**C**) and Tβ4-LRP1 PLA (**D**) in human AAA and omental and tibial arteries, quantified in Supplemental Figure 6. Scale bars: **A** and **D**: 20 μm (scale bar in **D** applies to **C**); **B**: 50 μm. Int: intima; Med: media; Adv; adventitia.

We next sought to localize the Tβ4-LRP1 interaction in situ within the aortic wall and to investigate subcellular localization of the complex. Proximity ligation assay (PLA) demonstrates close association of proteins (< 40 nm), and foci of Tβ4-LRP1 PLA signals were detected within medial VSMCs of murine aorta (Figure 3B). Specificity for the PLA was ensured by lack of signal in Tβ4^–/Y^ aortas (Figure 3B) and with omission of the LRP1 antibody (shown in Figure 4D). We examined localization of Tβ4-LRP1 foci more closely in primary murine aortic VSMCs. By immunofluorescence, LRP1 localized to punctate structures, consistent with its known incorporation into endocytic vesicles (Figure 3C). While Tβ4 was distributed throughout the cytoplasm and nucleus as expected, we observed strong puncta, suggesting that it may also localize to endosomal compartments. Indeed, Tβ4-LRP1 PLA signals overlapped with endosomes, shown in early endosomes by early endosome antigen 1 (EEA1) expression (Figure 3C).

Conservation across species was confirmed by detection of Tβ4-LRP1 PLA signals in human VSMCs, both in aneurysmal aorta from AAA patients prospectively recruited to the Oxford Abdominal Aortic Aneurysm Study (35) and healthy vessels (omental artery biopsies from the same patients). Further comparisons were made with nonaneurysmal tibial arteries from patients with lower limb occlusive arterial disease (Figure 4). By immunofluorescence, Tβ4 levels were higher in AAA samples than in other arteries, while LRP1 levels were comparable (Figure 4A, quantified in Supplemental Figure 6). In the absence of a healthy aorta comparison, Tβ4 levels cannot be correlated with or causally implicated in disease, given the structural and functional differences between the aorta and smaller caliber arteries. However, examination of adjacent sections suggested that Tβ4 levels may be elevated in medial layer cells, which were more synthetic in phenotype, with Tβ4 levels appearing to correlate with Caldesmon but not αSMA levels across different regions of the same aorta (Figure 4B). Overall, Caldesmon and Tβ4 are expressed in a gradient, with higher levels in modulated VSMCs closer to the adventitia than intima and in outgrowths within the adventitia (Figure 4B), as occurs during aneurysmal aortic wall remodeling. In contrast, αSMA shows relatively uniform expression throughout the medial layer. These observations are consistent with our murine data that suggests that synthetic markers are upregulated prior to any substantial loss of contractile markers.

Since the evidence for dysregulated LRP1–PDGFR-β signaling in aortic disease primarily derives from murine studies, we examined pathway activity and noted moderately higher levels of activated (Tyr1021-phosphorylated) PDGFR-β in AAA, compared with omental and tibial arteries, the caveat of different artery comparisons notwithstanding (Figure 4C, quantified in Supplemental Figure 6). Tβ4-LRP1 association was detectable by PLA in both AAA and nonaneurysmal arteries (Figure 4D, quantified in Supplemental Figure 6). These observations support an in situ association of Tβ4-LRP1 in the vessel wall and, together with the interaction data, point to a potential role for Tβ4 in modulating the vasculoprotective function of LRP1.

### Loss of Tβ4 increases susceptibility to aortic aneurysm.

**Figure 5:**
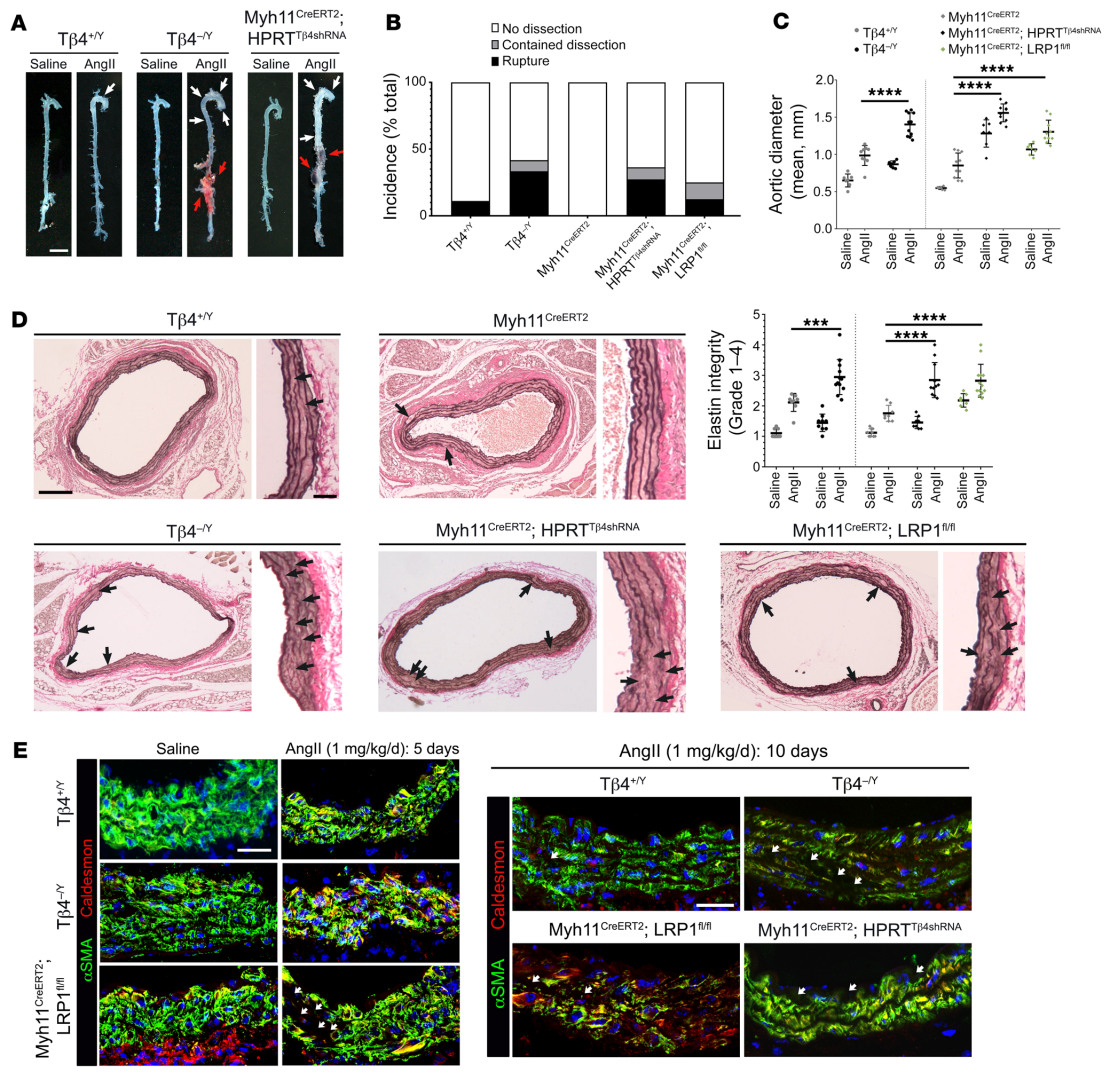
Mice lacking Tβ4 display more severe aneurysmal phenotypes, associated with augmented VSMC phenotypic switching. Whole-mount aortas from saline- or AngII-infused mice (**A**). White arrowheads indicate ascending and descending aortic aneurysms; red arrowheads indicate rupture. Incidence of dissection and rupture quantified per genotype in (**B**). Verhoeff–van Gieson staining of abdominal aorta to quantify aortic diameter (**C**) and visualize elastin integrity (**D**, breaks indicated with black arrowheads), quantified as elastin degradation score (as illustrated in Supplemental Figure 1). Immunofluorescence to assess medial layer morphology and VSMC phenotype at 5 and 10 days of AngII infusion (**E**). White arrowheads indicate regions devoid of VSMC markers, consistent with cell death. Data are mean ± SD, with each data point representing an individual animal. Significance was calculated using 1-way ANOVA with Tukey’s multiple comparison tests (**C** and **D**). ***P ≤ 0.001; ****P ≤ 0.0001. Samples were harvested after 10 days of AngII infusion, except in **E** (left, 5 days). Scale bars: **A**: 2 mm; **D**: 500 μm; inset 100 μm; **E**: 50 μm.

Our assessment of the Tβ4^–/Y^ and Myh11^CreERT2^ Hprt^Tβ4shRNA^ vasculature at baseline reveals defects that phenocopy those previously reported in smooth muscle–specific *Lrp1*^–/–^ mice (12, 13, 20) (aortic dilatation, disrupted elastin layers, and poorly differentiated VSMCs; Figures 1 and 2 and comparison with Myh11^Cre^ *Lrp1*^fl/fl^ aortas in Supplemental Figure 7). These observations not only support a common pathway but also suggest that Tβ4-KO mice may, like LRP1 nulls, be similarly predisposed to develop aortic aneurysm. We tested this supposition, using the well-characterized murine model, in which aneurysm is induced by Angiotensin II infusion (AngII; 1 mg/kg/d) via a subcutaneously implanted osmotic mini pump. AngII at this dose is shown to minimally affect blood pressure in mice, but is thought to activate the angiotensin II type 1 (AT1) receptor on infiltrating leukocytes to promote their recruitment and adhesion (36). Inflammatory macrophages and neutrophils secrete proteases that initiate degradation of the medial ECM (37). VSMCs respond to injury by undergoing phenotypic modulation; synthetic VSMCs are more proliferative and migratory and also secrete elastolytic proteases to exacerbate the destruction of the elastin lamellae, leading to dilatation and frequently rupture or dissection (38). Analysis of whole-mount aortas after 10 days of AngII infusion revealed an increased susceptibility of Tβ4^–/Y^ mice to aneurysm, compared with Tβ4^+/Y^ controls (Figure 5A). Phenotypes ranged from more pronounced ascending and descending aortic aneurysm (defined as > 1.5-fold dilatation; mild) to abdominal aortic rupture, hematoma formation, and death in less than 5 days (severe) (quantified in Figure 5B; examples of ruptures shown in Supplemental Figure 8 were excluded from d10 analysis in Figure 5). Although rare (< 10%), dissections were detectable in Tβ4^–/Y^ as blood tracking into the adventitial matrix or between medial and adventitial layers (Supplemental Figure 8). Given the prominent role of inflammation in driving aneurysm progression with AngII treatment and the numerous antiinflammatory roles ascribed to Tβ4 (28, 29, 39), we also investigated aneurysm susceptibility in tamoxifen-dosed Myh11^CreERT2^ Hprt^Tβ4shRNA^ knockdown mice, alongside Myh11^CreERT2^ Hprt^+/+^ controls (Figure 5A) and Myh11^CreERT2^ *Lrp1*^fl/fl^ mice, in order to avoid disrupting Tβ4-LRP1 function in immune cells. Similar to global Tβ4 knockouts, VSMC-specific Tβ4 knockdown mice displayed an increased incidence of rupture, as well as aortic aneurysms and a higher mortality rate over the 10-day time course (Figure 5, A–C and representative ruptures shown histologically in Supplemental Figure 8). Mean aortic diameter, measured on histological sections, was increased by 1.6-fold in Tβ4^–/Y^ mice and 1.8-fold in Myh11^CreERT2^ Hprt^Tβ4shRNA^ mice, compared with their respective AngII-treated controls. This compares with a 1.5-fold increased diameter in Myh11^Cre^ *Lrp1*^fl/fl^ aortas (Figure 5C). Elastin integrity was severely breached in Tβ4^–/Y^, Myh11^CreERT2^ Hprt^Tβ4shRNA^ and Myh11^Cre^ *Lrp1*^fl/fl^ aortas, with more breaks per section and higher mean integrity scores than in Tβ4^+/Y^ and Myh^CreERT2^ Hprt^+/+^ controls (2.95, 2.85, and 2.82 versus 2.11 and 1.76, respectively; Figure 5D). By these parameters, elastin degeneration in Tβ4^–/Y^ and Myh11^CreERT2^ Hprt^Tβ4shRNA^ aortas was comparable with that in Myh11^Cre^ *Lrp1*^fl/fl^ aortas (Figure 5D). Accentuated VSMC dedifferentiation was also evident in Tβ4^–/Y^ and Myh11^CreERT2^ *Lrp1*^fl/fl^ aortas, compared with respective controls (Tβ4^+/Y^ only shown, Figure 5E). At 5 days, the prominent morphological alterations were accompanied by increased expression of synthetic markers Caldesmon (Figure 5E and Supplemental Figure 9A ) and Vimentin (Supplemental Figure 9B) and a reciprocal loss of contractile markers αSMA (Figure 5E) and Calponin (Supplemental Figure 9A). Consistent with this, proliferation levels increased in some Tβ4^–/Y^ aortas compared with controls (*Ccnd1* qRT-PCR; Supplemental Figure 9C). After 10 days of AngII infusion, a further shift in phenotype was observed, along with a greater VSMC loss, in Tβ4^–/Y^, Myh11^CreERT2^ Hprt^Tβ4shRNA^, and Myh11^Cre^ *Lrp1*^fl/fl^ aortas compared with controls (Figure 5E). VSMC degeneration is rapid in the AngII infusion model and detection of TUNEL^+^ apoptotic cells within the medial layer was rare; most aortas lacked TUNEL^+^ VSMCs and, in fact, intimal endothelial cells and adventitial cells were more frequently TUNEL^+^ than VSMCs (Supplemental Figure 9D). Although there appear to be more TUNEL^+^ VSMCs in Tβ4-null aortas, quantification of apoptosis in this model is confounded since severely affected medial regions, which include large numbers of necrotic cells devoid of VSMC markers, typically contain fewer TUNEL^+^ nuclei than regions with less advanced disease, as exemplified in the comparison of Tβ4^–/Y^ at d5 versus d10 (Supplemental Figure 9D). Thus, we cautiously avoid overstating conclusions around the extent of apoptosis in aneurysmal Tβ4-null aortas.

**Figure 6:**
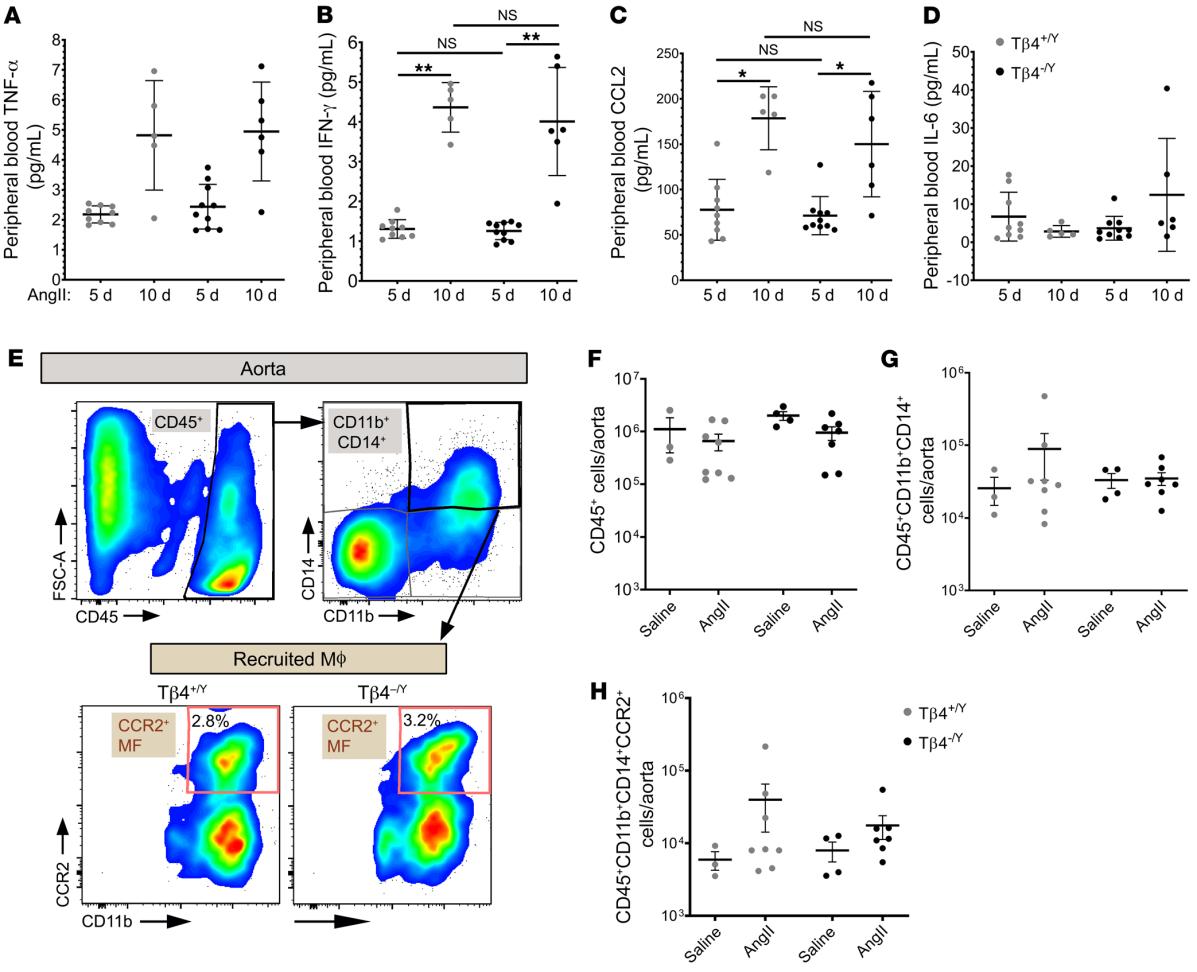
Inflammatory responses to AngII are not significantly altered with loss of Tβ4. Sera from Tβ4^+/Y^ and Tβ4^–/Y^ mice were assayed by multiplexed automated ELISA for TNF-α (**A**), IFN-γ (**B**) CCL2 (**C**), and IL-6 (**D**) levels after 5 and 10 days of AngII infusion. Flow cytometry was used to quantify macrophages (Mϕ) recruited to aortas of Tβ4^+/Y^ and Tβ4^–/Y^ mice after 5 days of AngII (gating strategy shown in **E**, with percentage of CCR2^+^ macrophages out of total live cells shown). Quantification of total CD45^+^ leukocytes (**F**), CD11b^+^CD14^+^ monocytes (**G**), and CCR2^+^ recruited macrophages (**H**). Significance was calculated using 1-way ANOVA with Bonferroni’s correction for multiple comparisons. *P ≤ 0.05; **P ≤ 0.01.

Beyond using a VSMC-specific targeting strategy, we sought to further exclude a causative difference in inflammatory responses between genotypes by determining expression of proinflammatory cytokines in peripheral blood by multiplexed automated ELISA. While the levels of tumor necrosis factor α (TNF-α), interferon γ (IFN-γ), and C-C motif chemokine 2 (CCL2) increased markedly over the course of AngII infusion, there were no significant differences between genotypes at any time point. IL-6 levels were unchanged between d5 and d10 of AngII treatment and also unaffected by loss of Tβ4 (Figure 6, A–D). This was borne out in the quantification of immune cells recruited to the aorta (Figure 6, E–H). Aortic leukocytes (CD45^+^) and monocytes (CD45^+^CD11b^+^CD14^+^) were assessed by flow cytometry. Recruited monocytes were further defined based on expression of the chemokine receptor CCR2, implicated in recruitment of monocyte-derived macrophages to the aorta and development of AAA (40). Although the numbers of macrophages (CD45^+^CD11b^+^CD14^+^CCR2^+^) increased with 5 days of AngII treatment, compared with saline, there were no differences between Tβ4^+/Y^ and Tβ4^–/Y^. Collectively, these data demonstrate that loss of Tβ4 predisposes to aortic aneurysm, phenocopying VSMC-specific LRP1 mutants. Exacerbated disease progression in Tβ4 mutants does not relate to aggravated inflammation, rather to the more advanced VSMC phenotypic modulation and degeneration of the elastin lamellae.

### LRP1-mediated signaling and endocytosis are dysregulated in the absence of Tβ4.

**Figure 7:**
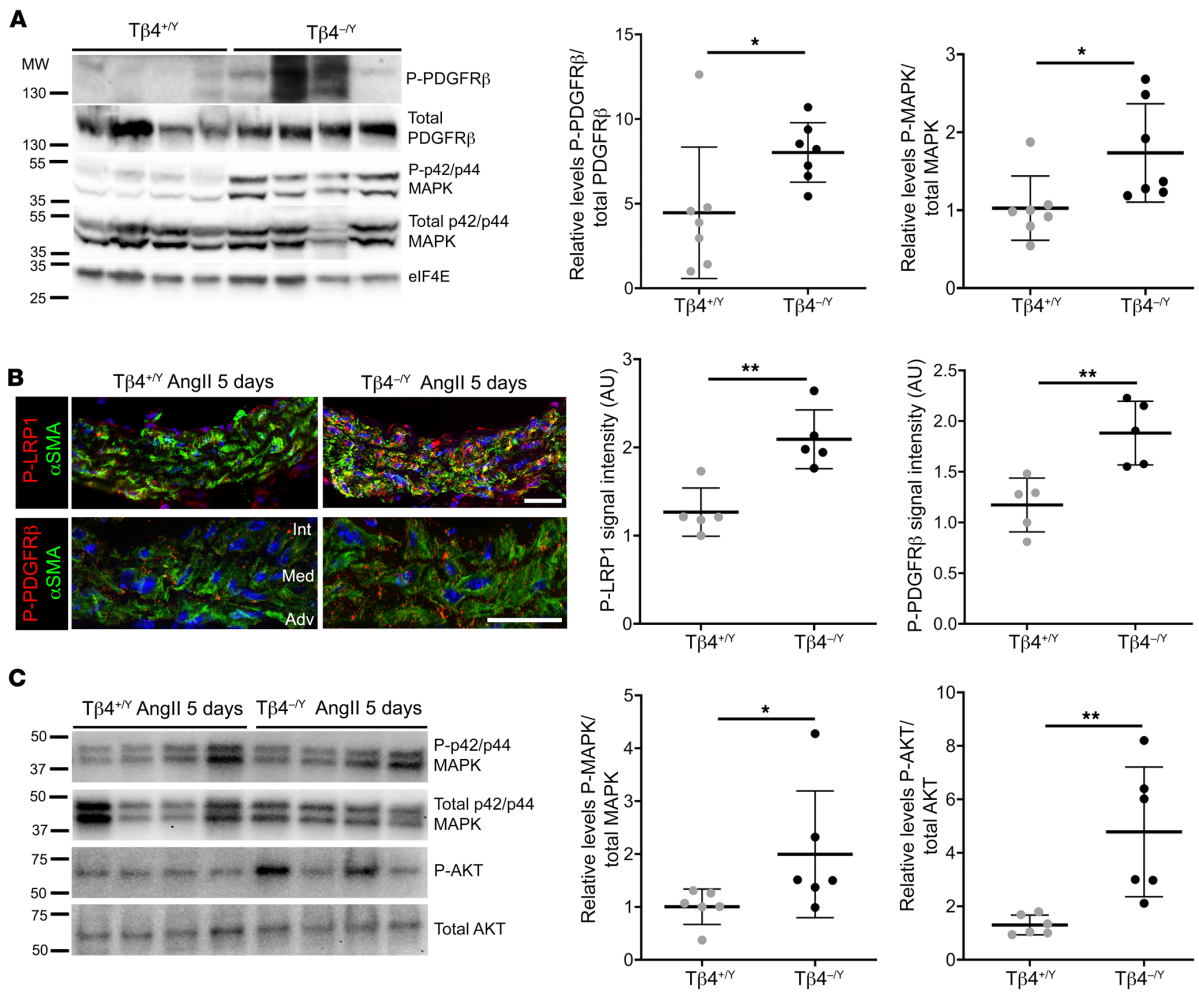
LRP1-mediated signaling is dysregulated in Tβ4-null aortas. Baseline PDGFR-β pathway activity in Tβ4^+/Y^ and Tβ4^–/Y^ aortas from adult mice, determined by Western blotting for phosphorylated PDGFR-β and p42/p44 MAPK, relative to total levels (**A**; representative of *n* = 7). Phospho-LRP1 (Tyr 4507) and Phospho-PDGFR-β (Tyr1021) levels in AAA VSMCs after 5 days of AngII infusion (**B**) and Western blotting of downstream effectors, phosphorylated p42/p44 MAPK and AKT, expressed relative to total p42/p44 MAPK and AKT, respectively (**C**; representative of *n* = 6). Data are mean ± SD, with each data point representing an individual animal. Significance was calculated using a Mann-Whitney nonparametric test. *P ≤ 0.05; **P ≤ 0.01. int: intima; med: media; adv: adventitia. Scale bars: **B**: 50 μm.

The VSMC-autonomous protective functions of LRP1 have largely been attributed to its role in regulating endocytosis of PDGFR-β (12, 21) and secreted ligands that control cellular phenotype by remodeling the ECM (14, 15). We therefore sought further evidence for interaction of Tβ4 with the LRP1 pathway, by investigating relevant functional readouts of the affected pathways. PDGF-BB binding to PDGFR-β stimulates autophosphorylation at multiple tyrosine residues. PDGFR-β tyrosine kinase activity requires LRP1-mediated endocytosis and, in turn, leads to phosphorylation of LRP1 on its intracellular domain (Tyr 4507) to facilitate adaptor protein binding and activation of downstream pathways (41–43), including phosphatidylinositol 3-kinase (PI3K), AKT, p42/p44 MAP kinase (ERK1/2), and c-Jun N-terminal kinase (JNK) (44). Even at baseline, a modest increase in pathway activity was apparent in Tβ4^–/Y^ adult mouse aortas, as shown by Western blotting for phospho-PDGFR-β (Tyr1021) and phospho p42/p44/MAP kinase (Figure 7A). Elevated PDGFR-β signaling in Tβ4^–/Y^ was further exacerbated in injury. After 5 days of AngII infusion, when injury-induced phenotypic switching was initiated, 1.6- and 1.5-fold increases in phospho-LRP1 and phospho–PDGFR-β, respectively, were observed in Tβ4^–/Y^ compared with Tβ4^+/Y^ VSMCs (Figure 7B). This was reflected in enhanced activation of downstream effector kinases p42/p44 MAPK and AKT, quantified by immunoblotting of aortic lysates (Figure 7C).

As well as controlling growth factor signaling, LRP1 governs VSMC phenotype by regulating endocytic turnover of a number of secreted matricellular proteins that dynamically remodel components of the ECM. In particular, connective tissue growth factor (CTGF), a multifunctional protein that modulates the interaction of cells with the matrix (45), high-temperature requirement factor A1 (HTRA1), an elastolytic serine protease (46), and plasminogen activator inhibitor-1 (PAI-1), a serine protease inhibitor (47), are LRP1 ligands known to impact VSMC phenotype and disease progression (15, 48). An accumulation of CTGF, HTRA1, and PAI-1 was observed in the medial VSMCs of Tβ4^–/Y^ compared with Tβ4^+/Y^ aortas (Supplemental Figure 10). Increased levels of CTGF and HTRA1 were reported in VSMC-specific *Lrp1*-KO mice (15), consistent with the notion of a common regulatory mechanism involving LRP1 and Tβ4.

**Figure 8:**
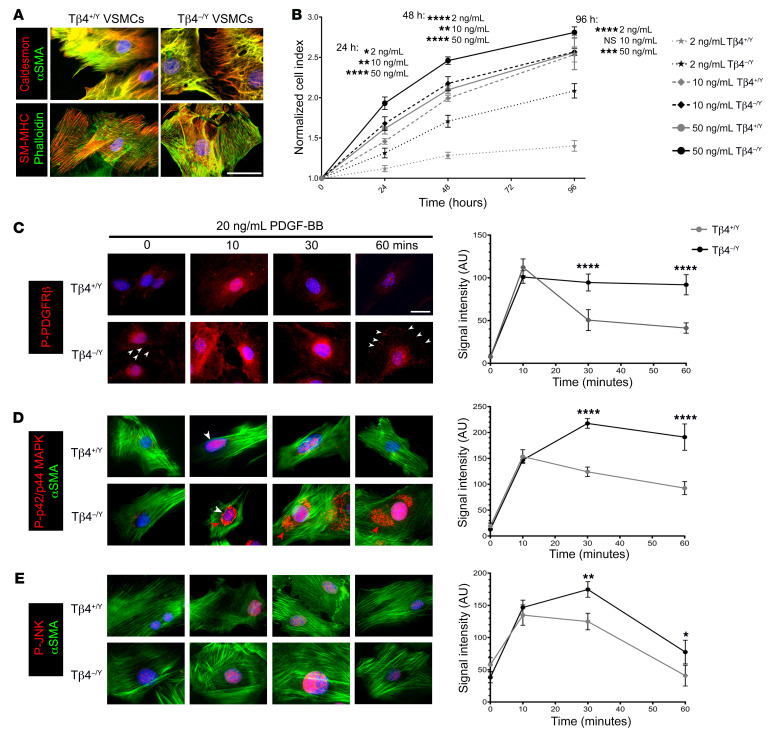
PDGFR-β signaling is dysregulated in Tβ4-null aortic VSMCs. Isolated VSMCs from Tβ4^+/Y^ and Tβ4^–/Y^ aortas (**A**). Proliferation curves of VSMCs treated with 2, 10, and 50 ng/mL PDGF-BB (**B**). Time course of PDGF-BB treatment and quantitative immunofluorescence of phosphorylated PDGFR-β (**C**), p42/p44 MAPK (**D**), and JNK (**E**). In **C**, white arrowheads highlight cell surface staining; in **D**, white arrowheads show nuclear staining, red arrowheads indicate perinuclear (Golgi) staining. Data are mean ± SEM, *n* = 3 experiments, each from a separate VSMC isolation. Significance was calculated using 1-way ANOVA with Tukey’s post hoc tests. *P ≤ 0.05; **P ≤ 0.01; ***P ≤ 0.001; ****P ≤ 0.0001. Scale bars: 50 μm (scale bar in **C** applies to **D** and **E**).

Signaling responses were more closely interrogated in vitro in primary VSMC cultures established from the descending aortas of Tβ4^+/Y^ and Tβ4^–/Y^ mice. While some degree of VSMC dedifferentiation and contractile-synthetic phenotypic switching is inherent upon culture in high serum-containing medium, Tβ4^+/Y^ VSMCs typically exhibited a characteristic spindle-like morphology, whereas Tβ4^–/Y^ VSMCs were typically more rhomboid (Figure 8A). Although synthetic markers such as Caldesmon were expressed at comparable levels in the majority of VSMCs, expression of contractile VSMC markers, including αSMA and SM-MHC, was reduced in Tβ4^–/Y^ VSMCs. Given the augmented signaling in vivo, we hypothesized that VSMCs lacking Tβ4 would be more sensitive to PDGF-BB–stimulated cellular responses, such as proliferation and migration. Indeed, over a range of tested PDGF-BB doses (2 ng/mL to 50 ng/mL), proliferation rate was significantly greater in Tβ4^–/Y^ than in Tβ4^+/Y^ VSMCs (Figure 8B). By contrast, increased sensitivity to PDGF-BB did not correlate with enhanced migration, as assessed by scratch wound assay (Supplemental Figure 11). In fact, Tβ4^–/Y^ VSMCs migrated significantly more slowly than Tβ4^+/Y^ VSMCs. As this result was unexpected, we additionally compared migration rates in Myh11^CreERT2^ *Lrp1*^fl/fl^ VSMCs, which are similarly known to be hypersensitive to PDGF-BB, and found their migration likewise to be reduced compared with controls (Supplemental Figure 11).

To systematically compare signaling responses, serum-starved VSMCs were treated with 20 ng/mL PDGF-BB over a 60-minute time course for analysis of pathway components by immunofluorescence. Phosphorylation of PDGFR-β, p42/p44 MAPK, and JNK was strongly induced within 10 minutes of treatment (Figure 8, C–E). In Tβ4^+/Y^ VSMCs, phosphorylation of pathway components diminished by 30 minutes and returned to near baseline by 60 minutes. This contrasted with Tβ4^–/Y^ VSMCs, in which phosphorylation remained high (*P* < 0.0001 for PDGFR-β) or further increased between 10 and 30 minutes (*P* < 0.05 for p42/p44 MAPK, JNK) and remained significantly elevated (close to maximal Tβ4^+/Y^ levels) even at 60 minutes. These data reveal that PDGFR-β pathway activity is both enhanced in magnitude and more sustained in duration in Tβ4^–/Y^, compared with Tβ4^+/Y^ VSMCs, in response to the same dose of PDGF-BB. Of note, although only nuclear P-MAPK signals were quantified, a striking accumulation of perinuclear P-MAPK was also apparent in most Tβ4^–/Y^ but not Tβ4^+/Y^ VSMCs (Figure 8D). Association of tyrosine-phosphorylated MAPK with Golgi occurs during the G2/M phase of the cell cycle (49), which is consistent with enhanced proliferation in Tβ4^–/Y^ VSMCs.

### Tβ4 modulates LRP1–PDGFR-β signaling via receptor-mediated endocytosis.

**Figure 9:**
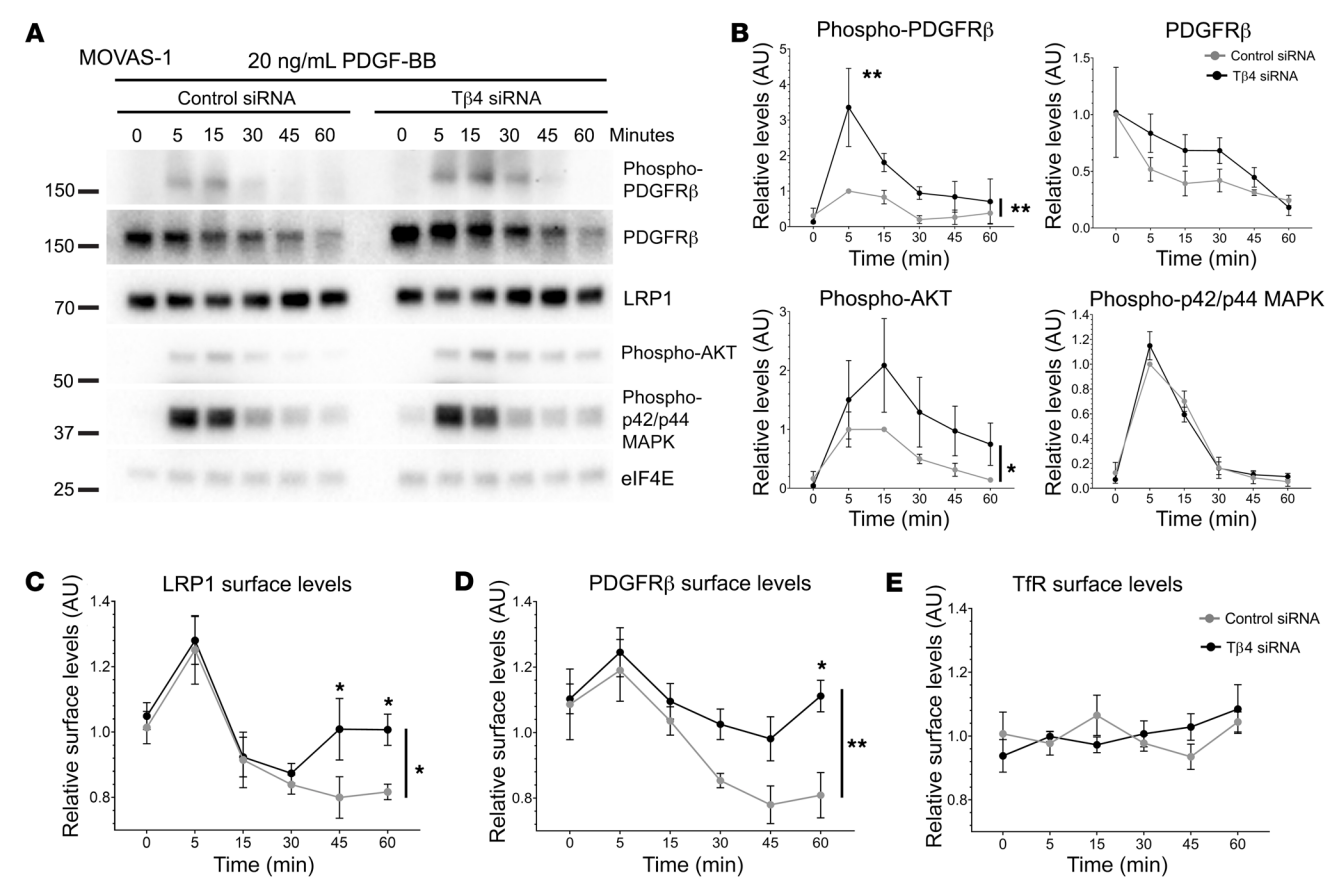
Enhanced sensitivity of VSMCs to PDGF-BB results from increased LRP1–PDGFR-β cell surface exposure. Western blotting to assess PDGFR-β pathway activation in MOVAS-1 cells over a 60-minute time course after treatment with 20 ng/mL PDGF-BB (**A**), quantified in (**B**). Surface biotinylation assays measure levels of LRP1 (**C**), PDGFR-β (**D**), and TfR (**E**) at the cell surface. Data are mean ± SEM; *n* = 3 experiments in **A** and **B**; *n* = 4 experiments in **C**–**E**. Significance was calculated using 1-way ANOVA with Tukey’s post hoc tests. Asterisks to the right of time course graphs indicate overall ANOVA significance for control versus Tβ4 siRNA; asterisks above data points denote significance for individual time points. *P ≤ 0.05; **P ≤ 0.01.

Endocytosis of PDGFR-β and its coreceptor LRP1 is required not only to transduce signals to downstream effectors but also to terminate pathway activity (21, 41). From endosomes, the LRP1–PDGFR-β complex may be recycled to the cell membrane or targeted for lysosomal degradation. Regulation of receptor trafficking critically determines sensitivity to PDGF ligands and magnitude of cellular responses. We, therefore, sought to investigate a role for Tβ4 in endocytosis of the LRP1–PDGFR-β complex, to determine if this may, at least in part, explain the effects of Tβ4 on PDGF-BB signaling. Initial studies were performed in MOVAS-1 murine aortic cells, with siRNA-mediated knockdown of Tβ4 (reduced to 10.1% ± 7.9% at the mRNA level; Supplemental Figure 12). Western blotting confirmed an enhanced and more sustained activation of components of the PDGFR-β pathway in serum-starved, Tβ4 siRNA-treated MOVAS-1, upon addition of 20 ng/mL PDGF-BB (Figure 9A). Y1021-phosphorylated PDGFR-β and S473-phosphorylated AKT were significantly enhanced, although phosphorylated p42/p44 was unaffected in Tβ4 knockdown MOVAS-1 (Figure 9B). Total PDGFR-β levels declined steadily over the 60 minutes, consistent with lysosomal targeting and degradation and decline was notably slower in Tβ4 knockdown cells (Figure 9B). In parallel, we performed surface biotinylation assays to measure levels of LRP1 and PDGFR-β at the cell membrane over the same 60-minute time course of PDGF-BB treatment. Surface levels of LRP1 peaked after 5 minutes of treatment, with a comparable fold-change and rate of decline in knockdown and control cells up to 30 minutes (Figure 9C), suggesting that Tβ4 is not required for translocation of receptors to the membrane or the initial steps of receptor-mediated endocytosis. However, whereas LRP1 levels in control cells declined further and remained significantly below baseline through to 60 minutes, consistent with the expected degradation (50), LRP1 levels in Tβ4 siRNA-treated cells recovered almost to baseline by 45 minutes (Figure 9C). Similar profiles were observed for PDGFR-β, except that the rate of decline in surface levels was more gradual in Tβ4 siRNA-treated cells, compared with controls. Levels fell between 5 and 45 minutes of treatment. Thereafter, a steep recovery of surface levels was observed in knockdown but not in control cells (Figure 9D). In contrast, surface levels of the transferrin receptor (TfR), which constitutively internalizes and rapidly recycles the iron carrier protein in a ligand-independent manner via receptor-mediated endocytosis (51), were not affected by loss of Tβ4, thereby ruling out a generalized endocytosis defect (Figure 9E). Collectively, these data suggest that in the absence of Tβ4, LRP1–PDGFR-β complexes are preferentially recycled to the cell surface and proportionally fewer receptors are targeted for lysosomal degradation, leading to enhanced and sustained pathway activation.

**Figure 10:**
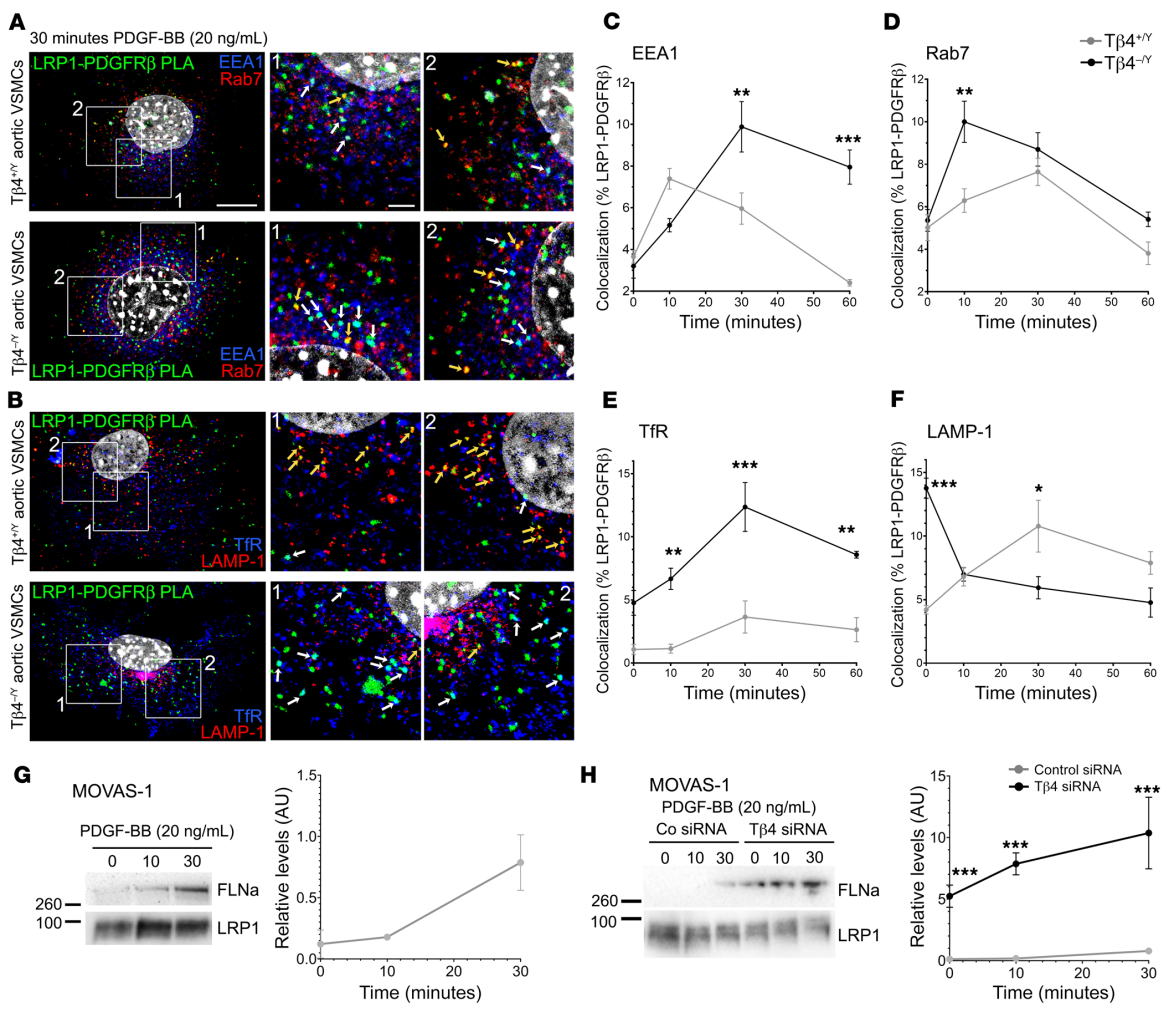
Loss of Tβ4 leads to dysregulated LRP1–PDGFR-β receptor trafficking. LRP1–PDGFR-β complexes, identified by PLA in primary aortic VSMCs, trafficking through endocytic compartments over a 60-minute time course (**A**–**F**). Shown at 30 minutes, colocalizing with early endosomes (EEA1; white arrows, **A**) and late endosomes (Rab7; yellow arrows, **A**) and with recycling endosomes (TfR; white arrows, **B**) and lysosomes (LAMP-1; yellow arrows, **B**). Quantification of colocalization in (**C**–**F**). By Western blotting, FLNa levels increase in MOVAS-1 in response to PDGF-BB treatment (**G**); FLNa levels are significantly elevated with reduced Tβ4 (Tβ4 siRNA vs Co siRNA; **H**). Data are mean ± SEM; *n* = 3 experiments, each from a separate VSMC isolation. Significance was calculated using 1-way ANOVA with Tukey’s post hoc tests. *P ≤ 0.05; **P ≤ 0.01; ***P ≤ 0.001. Scale bars: **A**: 5 μm (applies to all whole-cell views in **A** and **B**); boxed areas 1 and 2 shown magnified to left, with scale 2 μm (applies to all magnified views).

To test this notion further, we tracked the subcellular trafficking of LRP1–PDGFR-β complexes (as PLA signals) through successive endocytic compartments over the 60-minute PDGF-BB time course, in primary murine aortic VSMCs from Tβ4^+/Y^ control and Tβ4^–/Y^ Tβ4 null mice (Figure 10, A–F). Using antibodies that preferentially label early endosomes (EEA1), late endosomes (Rab7), recycling endosomes (TfR) and lysosomes (LAMP-1), we quantified the proportion of LRP1–PDGFR-β colocalization to each compartment using the ImageJ plug-in JACoP v2.0 (52). In Tβ4^+/Y^ control cells, LRP1–PDGFR-β internalization within EEA1^+^ early endosomes and Rab7^+^ late endosomes increased over the first 10 and 30 minutes’ treatment, respectively, before declining to below baseline levels (serum-starved cells; Figure 10, A, C, and D). In Tβ4^–/Y^ cells, the proportions of LRP1–PDGFR-β in early endosomes increased further between 10 and 30 minutes and remained elevated in both early and late endosomes over the 60-minute time course, compared with control VSMCs (Figure 10, A, C, and D). In Tβ4^+/Y^ control cells, a relatively small degree of LRP1–PDGFR-β recycling was observed (TfR^+^ recycling endosomes; Figure 10, B and E), and redistribution into LAMP-1^+^ lysosomes peaked after 30 minutes of treatment (Figure 10, B and F). Recycling of LRP1–PDGFR-β in Tβ4^–/Y^ cells was significantly elevated at all time points, peaking at 30 minutes (Figure 10, B and E), and was accompanied by a corresponding decline in levels of lysosomally targeted LRP1–PDGFR-β (Figure 10, B and F), albeit from an unexpectedly elevated baseline level in Tβ4^–/Y^ VSMCs. These data indicate a requirement for Tβ4 in the downmodulation of PDGFR-β signaling, following acute stimulation by PDGF-BB via the targeting of LRP1–PDGFR-β complexes to lysosomes.

The endocytosis data suggest that the Tβ4-LRP1 interaction is not required for the initial activation and internalization of LRP1–PDGFR-β complexes; rather, that Tβ4 influences the differential sorting of complexes, either for lysosomal destruction or receptor recycling to potentiate PDGF-BB signaling. To gain insight into the molecular mechanisms underlying this process, we performed proteomic analyses to identify proteins immunoprecipitated with LRP1 from aortic lysates (Supplemental Table 2). This was complemented by identification of proteins that immunoprecipitated with LRP1 and PDGFR-β from MOVAS-1 cells, before and 10 minutes after treatment with 20 ng/mL PDGF-BB (Supplemental Table 2). Mass spectrometry identified multiple clathrin coat proteins, myosins, and other components of the endocytic machinery. Of particular note, Filamin A (FLNa) was detected in both aorta and MOVAS-1 and its interaction with PDGFR-β increased significantly after 10 minutes of PDGF-BB treatment (Supplemental Table 2). FLNa crosslinks F-actin into orthogonal networks and mediates recycling of a broad range of membrane receptors, including the chemokine receptor CCR2b, β2-adrenergic receptor, and calcitonin receptor, by controlling receptor entry into endosomal actin microdomains that recruit cargo for the rapid recycling pathway (53, 54). A clear correlation exists between FLNa levels and receptor fate, with high FLNa promoting recycling and lower levels favoring lysosomal targeting. Rapid switching between these fates is fine-tuned by proteolytic degradation of FLNa (55). Indeed, we observed a rapid stabilization of FLNa in MOVAS-1 cells, and upregulation within 10 to 30 minutes of PDGF-BB addition (Figure 10G), presumably to prevent complete degradation of the PDGFR-β pool. Remarkably, in Tβ4 knockdown MOVAS-1, FLNa levels were 43-fold higher than control cells in the absence of PDGF-BB and still increased further after PDGF-BB treatment (Figure 10H). Elevated FLNa levels may indeed explain the preferential sorting of LRP1–PDGFR-β complexes into recycling endosomes, although the basis for Tβ4-dependent (dys)regulation of FLNa will require further investigation. Collectively, our data support a mechanism of LRP1–PDGFR-β endocytic sorting that is controlled by Tβ4 and FLNa, to modulate cell surface receptor levels and ligand sensitivity. A failure to adequately attenuate signaling in Tβ4^–/Y^ mice may explain the advanced dedifferentiation (synthetic phenotype) observed at baseline, which is further exacerbated when PDGF-BB levels increase during disease.

### Restoration of normal PDGFR-β signaling rescues aneurysmal phenotype of Tβ4 null mice.

**Figure 11:**
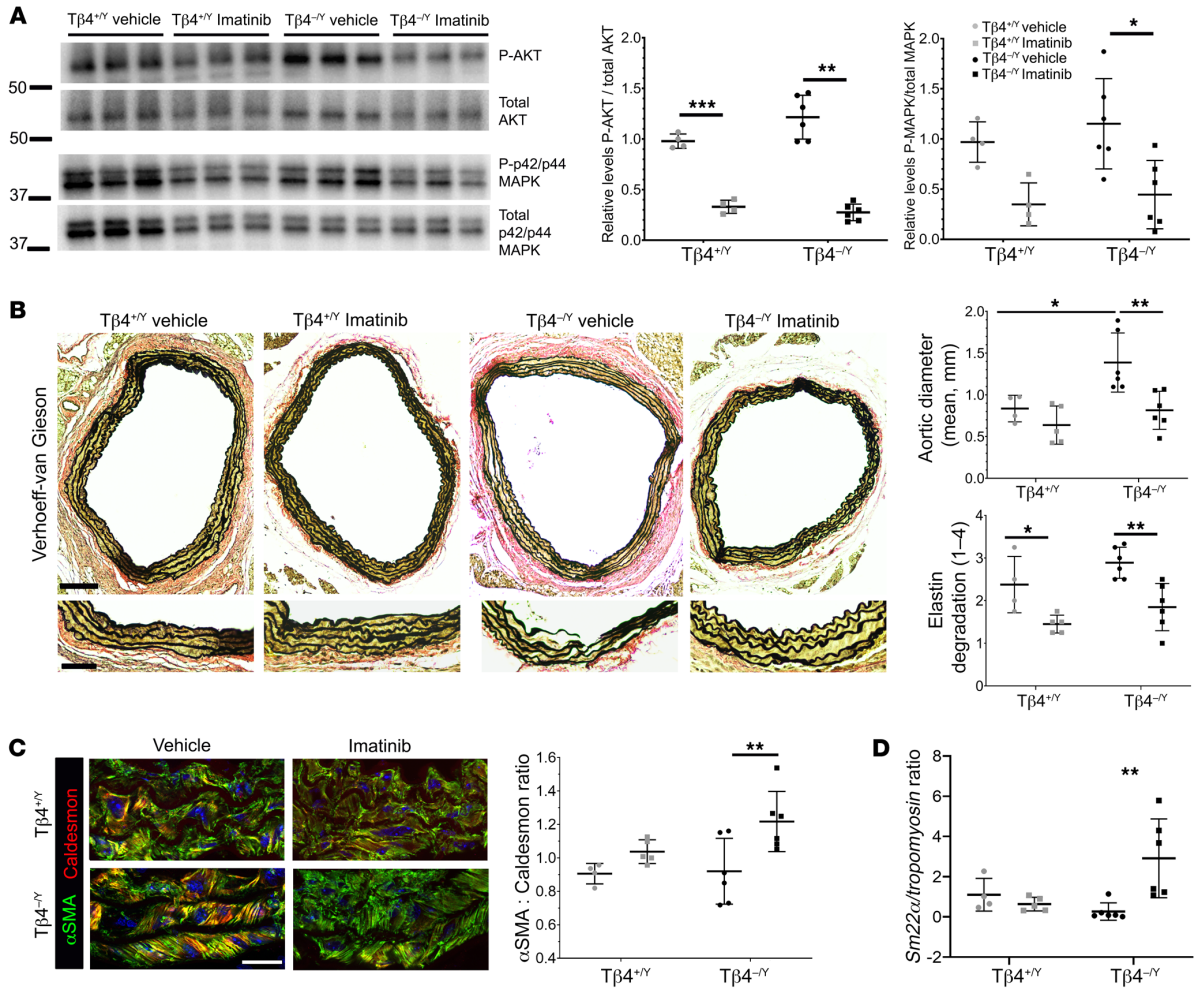
Restoration of normal PDGFR-β signaling ameliorates aneurysmal phenotype of Tβ4-null mice. Imatinib attenuated PDGFR-β pathway activity in aortas of AngII-infused Tβ4^+/Y^ and Tβ4^–/Y^ mice, confirmed by Western blotting of downstream effectors, phosphorylated AKT, and p42/p44 MAPK, expressed relative to total AKT and p42/p44 MAPK, respectively (**A**). Loading control is eIF4e, shown in Supplemental Figure 13C. Verhoeff–van Gieson staining (**B**) to quantify aortic diameter and assess elastin integrity (degradation score, as in Supplemental Figure 1). Immunofluorescence for αSMA (contractile) and Caldesmon (synthetic; **C**) and qRT-PCR for *Sm22α* (contractile) and *Tropomyosin* (synthetic; **D**), to further assess VSMC phenotype. All samples were harvested after 8 days of AngII infusion. Data are mean ± SD, with each data point representing an individual animal. Significance was calculated using 2-way ANOVA with Tukey’s multiple comparison tests. *P ≤ 0.05; **P ≤ 0.01; ***P ≤ 0.001. Scale bars: **B**: 500 μm, inset 100 μm; **C**: 50 μm.

To evaluate the extent to which dysregulated LRP1–PDGFR-β signaling predisposes to AngII-induced aneurysm, we sought to rescue the exacerbated phenotype of Tβ4^–/Y^ mice by pharmacological inhibition of the pathway. Imatinib (also known as Gleevec) is a tyrosine kinase inhibitor with relative specificity for PDGF receptors, as well as c-kit and Abl, which was shown to block autophosphorylation of PDGFR-β and tyrosine phosphorylation of the LRP1 ICD (12), and to protect in mouse models of aneurysm (56, 57) and atherosclerosis (12). Mice were gavaged with 10 mg/kg Imatinib or sterile water daily for 2 days prior to osmotic mini pump implantation (1 mg/kg/d AngII) and for 8 days after implantation until harvest. Imatinib significantly attenuated PDGFR-β signaling in both Tβ4^+/Y^ and Tβ4^–/Y^ aortas treated with AngII: P-PDGFR-β, *P* < 0.01; P-LRP1, *P* < 0.001; P-AKT, *P* < 0.001; P-ERK1/2, *P* < 0.01. Notably, levels of phosphorylated PDGFR-β and LRP1 (immunofluorescence, Supplemental Figure 13, A and B), as well as AKT and p42/p44 MAPK (Western blotting, Figure 11A) were comparable between Tβ4^+/Y^ and Tβ4^–/Y^ aortas after Imatinib treatment. Imatinib did not affect inflammation, assessed at the level of peripheral blood cytokine levels, which were unchanged by genotype or treatment (Supplemental Figure 14). We examined aortas histologically to determine whether restoration of signaling in Tβ4^–/Y^ aortas was sufficient to preserve vascular integrity. Verhoeff-van Gieson staining revealed a striking degree of aortic protection, which was more pronounced in Tβ4^–/Y^ than Tβ4^+/Y^ mice (Figure 11B); aortic diameter was reduced 1.3-fold in Tβ4^+/Y^ and 1.7-fold in Tβ4^–/Y^ and elastin degradation was reduced by 1.6-fold in both Tβ4^+/Y^ and Tβ4^–/Y^. VSMC phenotype was similarly preserved in Imatinib-treated Tβ4^–/Y^ aortas, with increased expression of contractile markers Calponin 1 (Western blot, Supplemental Figure 13C), αSMA (immunofluorescence, Figure 11C), and *Sm22a* (qRT-PCR, Figure 11D) and a corresponding decrease in expression of synthetic markers, Caldesmon (immunofluorescence, Figure 11C) and *Tropomyosin* (qRT-PCR, Figure 11D). While a similar trend was apparent in Imatinib-treated Tβ4^+/Y^ aortas, the magnitude of rescue was more modest and not statistically significant (Figure 11, C and D, and Supplemental Figure 13C).

## Discussion

Collectively, our results demonstrate a postnatal requirement for Tβ4 in VSMCs to maintain a differentiated, contractile phenotype, both in homeostasis and in the context of disease. Global and VSMC-specific Tβ4-null mice displayed increased susceptibility to aortic aneurysm and a higher incidence of dissection, rupture, and mortality. Accelerated disease progression was characterized by augmented contractile-synthetic VSMC switching and underpinned by dysregulated PDGFR-β signaling, which results from a failure to functionally regulate trafficking of PDGFR-β and coreceptor LRP1. Consistent with this, the defects we describe in Tβ4-null mice closely phenocopy those reported with VSMC-specific loss of LRP1, both during development (58) and in disease (12, 13, 20). Of note, these phenotypes manifest even when loss is induced postnatally, confirming a maintenance role for Tβ4-LRP1 in vascular homeostasis, rather than persistence of developmental defects predisposing to disease. Although we did not detect any overt exacerbation of inflammatory responses in global Tβ4^–/Y^ mice, it was important, due to the recognized roles for LRP1 and Tβ4 in endothelial cells (7, 59) and macrophages (60, 61), to distinguish a cell-autonomous VSMC role from potential paracrine contributions. However, it would be of interest to further investigate whether Tβ4 functionally regulates LRP1 in other cell types to influence vascular disease outcome.

From a clinical perspective, these findings are highly relevant. GWAS have identified LRP1 variants as major risk loci for AAA (16), carotid artery (17), and coronary artery disease (18). *TMSB4X* is the most abundant transcript in healthy and AAA aorta (6), yet the role of Tβ4 in vascular protection and regulation of LRP1-mediated growth factor signaling had not been recognized. Our study delineates a mechanism by which Tβ4 controls LRP1-mediated VSMC responses to protect against vascular disease. Paradoxically, we found Tβ4 levels to be higher in AAA compared with omental and tibial arteries, which may appear to contradict our demonstration of an exacerbated aneurysmal phenotype with loss of Tβ4. It is important to acknowledge the structural and functional differences between the aorta and smaller arteries, maintained by differential gene expression. A limitation of our study, in this regard, is the inaccessibility of healthy aorta controls. Thus, we cannot directly attribute increased Tβ4 to disease; rather, it may reflect a difference in artery size or function. In fact, this would be consistent with a cDNA array study that did not find *TMSB4X* to be differentially expressed between healthy aorta and AAA (6). However, further investigation into the role of Tβ4 in human aortic disease is warranted, since the array study did not distinguish cell-type–specific *TMSB4X* expression, and our qualitative assessment of AAA sections suggests that Tβ4 levels correlate with a synthetic, rather than contractile, smooth muscle phenotype. This may infer that Tβ4 levels are upregulated in synthetic VSMC populations to compensate for the dysregulated LRP1 function that is suggested from GWAS to occur in arterial disease. Further research should carefully address Tβ4 fluctuations within distinct cells of the medial layer and inflammatory infiltrate to determine causal versus consequential changes in relation to LRP1-regulated signaling and progression of aneurysmal disease.

The significance of VSMC phenotypic switching in aneurysm is still not fully understood, but recent studies demonstrate that clonal expansion of dedifferentiated VSMC subpopulations causes their outgrowth from the medial layer to invade the adventitia and false channel borders in AngII-induced mouse aortic aneurysm (62). Autophagy was shown to play an important role in eliminating these synthetic cells to preserve vessel integrity and reduce the occurrence and severity of aortic dissection. Understanding VSMC heterogeneity and identifying the regulators of contractile-synthetic switching may enable the fine tuning of VSMC phenotypes that are beneficial for repair. Interestingly, while loss of Tβ4 and LRP1 augmented PDGFR-β–stimulated proliferation in vitro, VSMC migration was, in our hands, inhibited. Although this finding is inconsistent with some studies demonstrating enhanced VSMC migration upon loss of LRP1 (13, 63), others have demonstrated inhibition, confirming a requirement for LRP1 (64, 65), just as there is a clear requirement for Tβ4 (66) in cell migration. Whether LRP1 promotes or inhibits migration of VSMCs appears to be dependent on extracellular matrix and ligand-binding cues (67, 68). Moreover, there are likely distinct paracrine stimulatory roles for Tβ4, in addition to direct effects upon remodeling of the actin cytoskeleton (69, 70) and further work is required to disentangle these.

PDGF-BB, secreted from infiltrating macrophages during the initiating phases of aortic disease, potently drives VSMC phenotypic switching. The vasculoprotective effects of endogenous Tβ4 that we report appear to be mediated, at least in part, via control of LRP1–PDGFR-β exposure on the cell surface to influence the sensitivity of VSMCs to PDGF-BB and potentially other ligands regulated by LRP1 (71). Tβ4 was found to alter cellular responses to PDGF-BB by shifting the balance between receptor degradation and recycling. De novo actin filament assembly is essential for endocytosis, particularly remodeling of structures at the cell surface to allow inward movement of vesicles. However, internalization of LRP1 and PDGFR-β is unaffected by loss of Tβ4, as is LRP1 ICD phosphorylation, which occurs upon internalization. Moreover, the Tβ4-LRP1 interaction and the lack of an effect on TfR endocytosis suggests a level of selectivity in the role of Tβ4, rather than a generic mechanism of receptor recycling based on actin cytoskeletal remodeling. Tβ4-mediated actin polymerization promotes fusion of late endosomes and lysosomes, but is not required for fusion of early endosomes (72). Reduced lysosomal targeting of LRP1–PDGFR-β is consistent with a defect in late endosome-lysosome fusion but this seems an unlikely explanation for the increased distribution to recycling endosomes, as cargoes are sorted directly from early to recycling endosomes, bypassing late endosomes (73). Recycling of signaling receptors occurs via a selective and carefully regulated mechanism, namely actin-stabilized sequence-dependent recycling tubule (ASSERT) scaffold formation (74), which also requires actin polymerization, thus a generalized defect in actin dynamics would be expected to impact this process. In our study, FLNa was identified to interact with LRP1 and PDGFR-β, and dramatically elevated levels in Tβ4 knockdown VSMCs may indeed account for the augmented receptor recycling. FLNa has been implicated in controlling the trafficking and fate of diverse receptors, including the calcitonin receptor (54), CCR2, and β-adrenergic receptor (53). Thus, Tβ4 may similarly influence endocytic regulation of certain other signaling receptors. It should be noted that a previous study reported a modest (0.76-fold) reduction of FLNa levels in sm*Lrp1*^–/–^ VSMCs (70) which, given the phenotypic similarity and inferred mechanisms, may appear discordant with the striking (43-fold) upregulation of FLNa in Tβ4-knockdown MOVAS-1. These findings may perhaps be explained by distinct Tβ4/LRP1 roles, directly or indirectly relating to FLNa expression, or by the contrasting requirement to compensate for loss of the respective proteins.

With the exception of actin, other Tβ4 interacting partners are classified as intrinsically disordered proteins, which lack well-defined 3D structure under native conditions yet fulfill important functions in signaling and physiological regulation (32). The interactions of Tβ4 with PINCH, ILK, and stabilin-2 are weak, transient, and fuzzy, involving specific partner recognition but without adoption of stable folded structures. We confirmed that the cytoplasmic tail of LRP1 is similarly disordered and interacts weakly with Tβ4. Structural disorder is proposed to be functionally advantageous, increasing speed of interaction and adaptability to different binding partners (32). The high intracellular concentrations of Tβ4 (300–600 μM) (75) permit weak complexes (micromolar *K_D_*) to form easily in cells that respond rapidly to external signals (32). While further work is required to pinpoint precisely when, and in which subcellular compartments, Tβ4 engages with LRP1, relative to LRP1–PDGFR-β internalization and activation, binding near the NPxY motifs supports a potential role in regulating signal transduction and/or engagement with the endocytic machinery (34, 43). Given the disease relevance of LRP1, not just in vascular disease but also in the pathogenesis of Alzheimer’s disease (76), further investigation into the mechanism controlling receptor trafficking is warranted. Prevalence of aortic aneurysm is 5% among the elderly and treatment involves a high-risk surgical procedure with no pharmacological therapeutic options. Understanding how turnover of LRP1 and its coreceptors is controlled, and how this impacts sensitivity and responses to disease-associated growth factors, raises the possibility of developing novel strategies to maintain differentiated VSMC phenotype and treat aortic disease.

## Methods

Detailed descriptions of the animal models, human tissue sampling, and experimental methods are provided in the Supplemental Methods. The mass spectrometry proteomics data have been deposited to the ProteomeXchange Consortium via the PRIDE partner repository with the data set identifier PXD024162.

### Statistics.

Randomization of animals to treatment or genotype groups was introduced at the time of mini pump implantation (aneurysm) or harvest (baseline). Thereafter, tissues were processed and analyzed by an independent observer blinded to treatment. Statistical analyses were performed with GraphPad Prism software. For the quantitative comparison of 2 groups, 2-tailed unpaired Student’s *t* test was used to determine any significant differences, after assessing the requirements for a *t* test using a Shapiro-Wilk test for normality and an *F* test to compare variances. Alternatively, a Mann-Whitney nonparametric test was used. For comparison of 3 groups or more, a 1-way ANOVA with Tukey’s post hoc test was used. For analyses involving 2 independent variables, a 2-way ANOVA with Bonferroni’s, Holm-Sidak, or Dunnett’s post hoc test was used, after Shapiro-Wilk test for normality. Significance is indicated in the figures, as follows: *P ≤ 0.05; **P ≤ 0.01; ***P ≤ 0.001; ****P ≤ 0.0001.

### Study approval.

All procedures involving the use and care of animals were performed in accordance with the Animals (Scientific Procedures) Act 1986 (Home Office, United Kingdom) and approved by the University of Oxford or University College London Animal Welfare and Ethical Review Boards. The Oxford Abdominal Aortic Aneurysm (OxAAA) study was approved by the Oxford regional ethics committee (reference: 13/SC/0250).

## Author contributions

SM, SB, and NS carried out experiments and data analysis, with additional data contributed by ANR, AJ, KND, SSH, RD, and GN. ANR and JP performed surgical procedures. RL and AH designed and conducted the OxAAA study, which enabled the human tissue analyses. KMC, RF, and MS provided valuable intellectual input. NS established the hypotheses, secured funding, supervised the study, and wrote the manuscript.

## Supplementary Material

Supplemental data

Supplemental Table 2

## Version 1 03/30/2021

In-Press Preview

## Version 2 05/17/2021

Electronic publication
