# Supplementary material for: Thymosin β4 protects against aortic aneurysm via endocytic regulation of growth factor signaling
Source: J Clin Invest. 2021 May 17;131(10):e127884. doi: 10.1172/JCI127884 (PMC8121525; doi:10.1172/JCI127884)

# SUPPLEMENTAL INFORMATION

## for

# Thymosin β4 protects against aortic aneurysm via endocytic regulation of growth factor signalling

Sonali Munshaw [1, 8], Susann Bruche[1, 8], Andia N. Redpath[1, 9], Alisha Jones[2, 3, 9], Jyoti Patel[4], Karina N. Dubé[5], Regent Lee[6], Svenja Hester[7], Rachel Davies[1], Giles Neal[1], Ashok Handa[6], Michael Sattler[2, 3], Roman Fischer[7], Keith M. Channon[4] & Nicola Smart[1]

1. Burdon Sanderson Cardiac Science Centre, Department of Physiology, Anatomy & Genetics, University of Oxford, Sherrington Building, South Parks Road, Oxford OX1 3PT, UK.

2. Institute of Structural Biology, Helmholtz Zentrum München, Neuherberg, 85764 München, Germany.

3. Biomolecular NMR and Center for Integrated Protein Science Munich at Chemistry Department, Technical University of Munich, Garching, 85748 München, Germany.

4. BHF Centre of Research Excellence, Division of Cardiovascular Medicine, John Radcliffe Hospital, University of Oxford, UK.

5. UCL-Institute of Child Health, 30 Guilford Street, London WC1N 1EH, UK

6. Nuffield Department of Surgical Sciences, University of Oxford, Oxford, UK

7. Nuffield Department of Medicine, Target Discovery Institute, University of Oxford, Oxford OX3 7FZ, UK.

8. These authors contributed equally to this work.

9. These authors contributed equally to this work.

Elastin Degradation Scoring

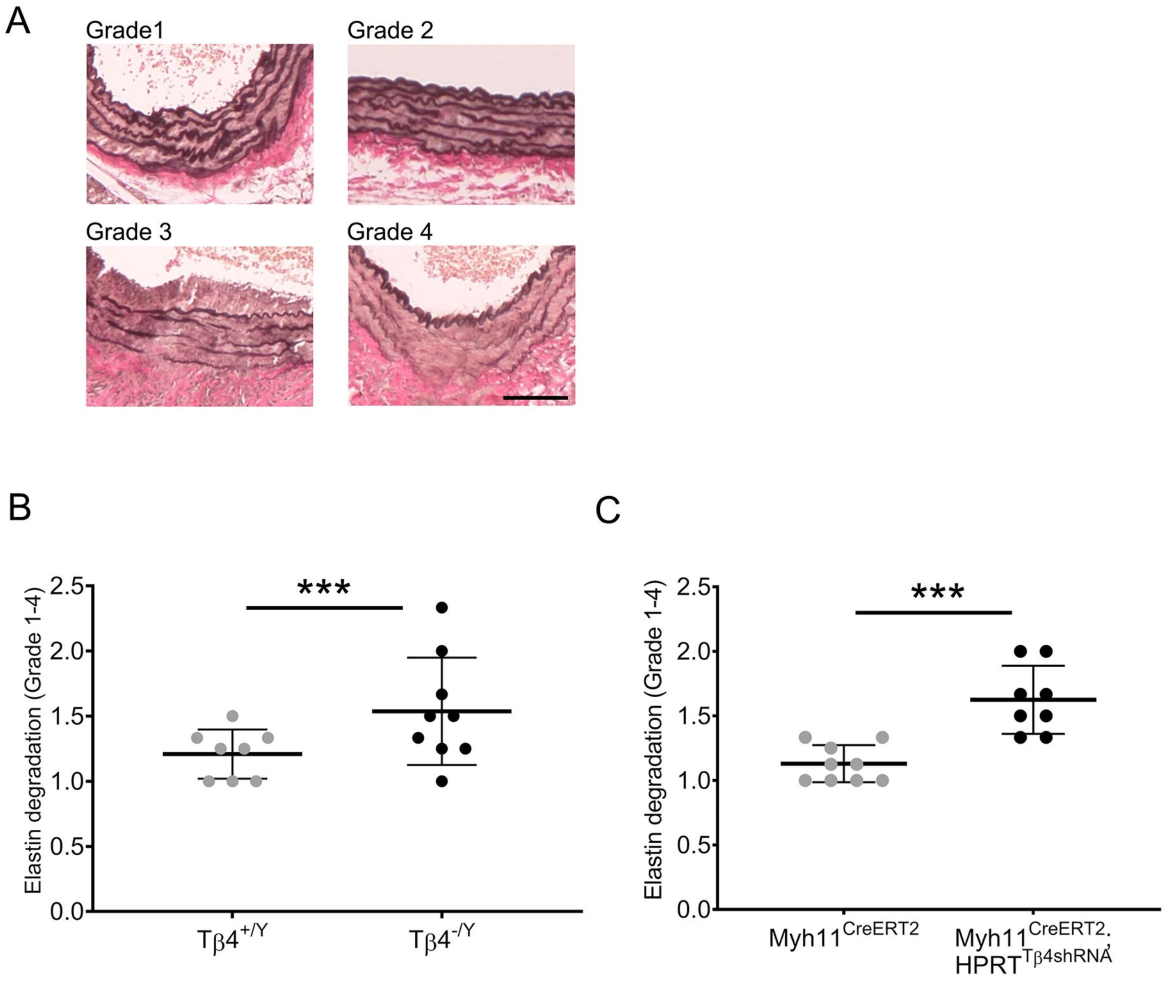


**Supplemental Figure 1. Visual scoring system used to assess severity of elastin degradation.**

**A**: Grades 1-4 relate to increasing severity (1=normal; 2=occasional breaks; 3= frequent breaks, multiple lamellae; 4=severe breakdown). Global (**B**) and VSMC-specific (**C**) loss of Tβ4 aortas were scored, whilst blinded to genotype. 6-8 sections per aorta were assessed and mean score plotted for each animal. Scale bar (applies to all panels): 100μm.

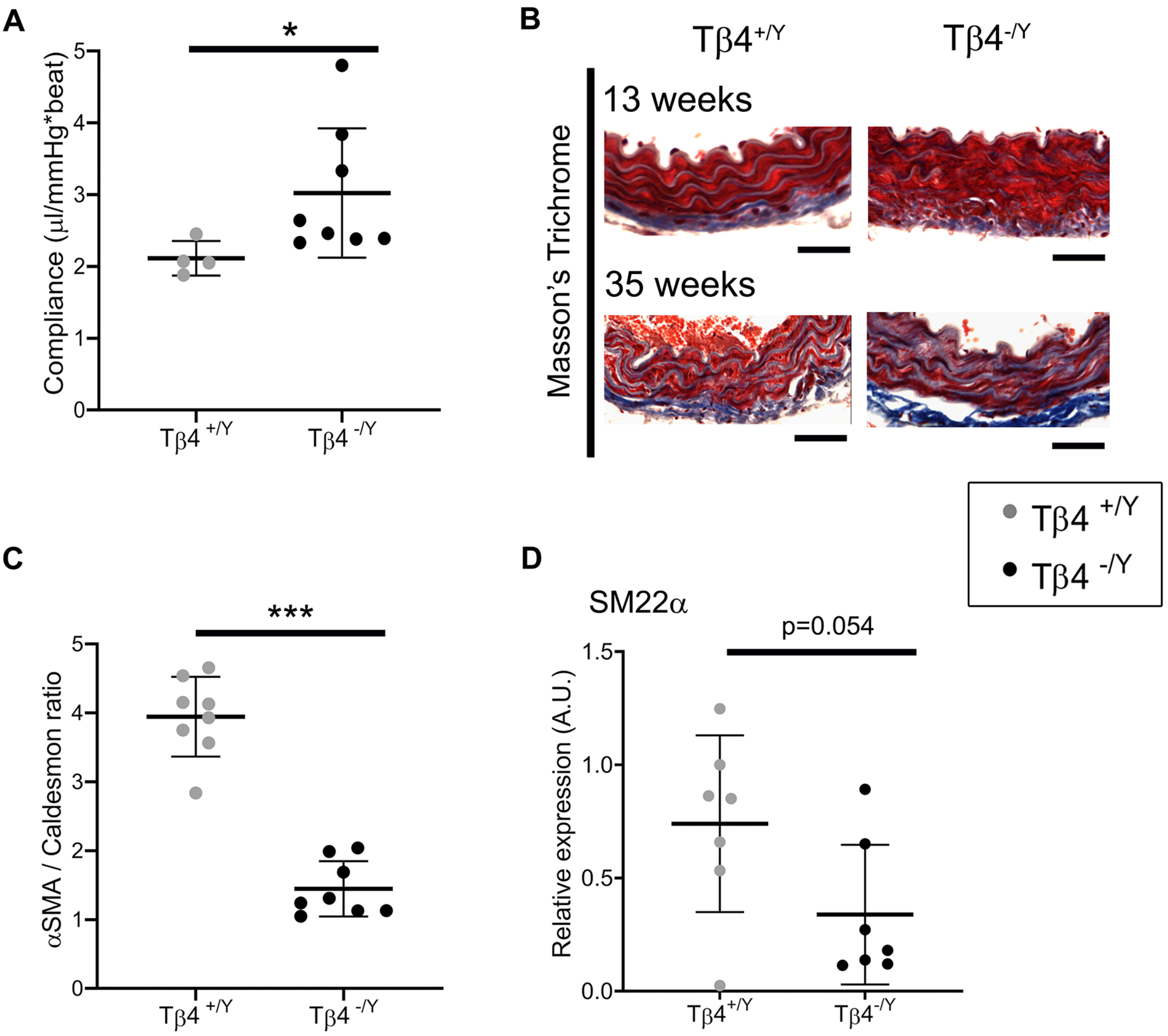


**Supplemental Figure 2. Tβ4-null mice display baseline aortic defects in adulthood (in conjunction with Figure 1). A:** MRI and arterial blood pressure measurements of vascular compliance (stroke volume/pulse pressure). Masson's Trichrome staining (**B**) to visualise fibrosis in young (13 week old) and aging (35 week old) adult mice. Contractile/synthetic smooth muscle markers were assessed by immunofluorescence (shown in Figure 1F; quantification of αSMA (contractile)/ Caldesmon (synthetic) maker ratios; **C**) and western blotting (shown in Figure 1F, quantification of SM22α in **D**). Data are presented as mean ± SD, with each data point representing an individual animal (12-16 week old male mice, except **B**, where a cohort of 35-week old mice were also assessed (n=6). Significant differences were calculated using Mann Witney non-parametric tests (**A, C-D**). * $P \leq 0.05$; *** $P \leq 0.001$ for Tβ4$^{+/Y}$ vs Tβ4$^{-/Y}$. Scale bars: **B**: 50µm.

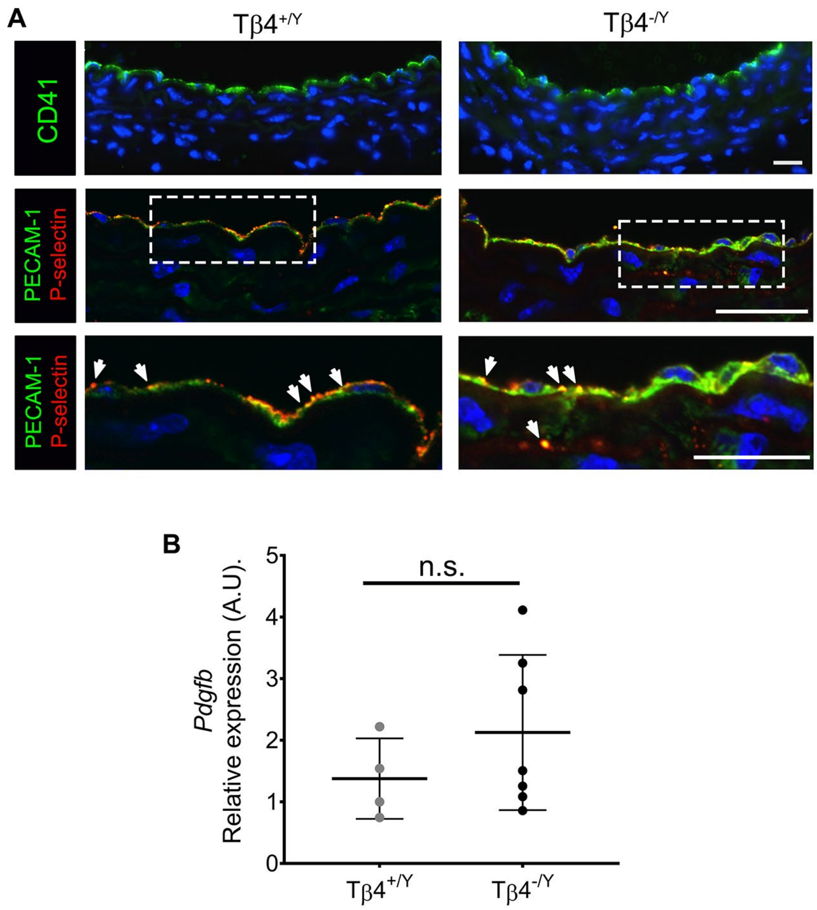


**Supplemental Figure 3. Platelet density is unaltered in Tβ4 null aortas. A**: Immunofluorescence to detect platelets (CD41+) and activated platelets (P-Selectin+/PECAM-1+) in Tβ4 $^{+/Y}$ and Tβ4 $^{-/Y}$ aortas. Boxed regions in second row are magnified in third row. **B**: Expression of *Pdgfb* was assessed by qRT-PCR from abdominal aortic extracts. Data are presented as mean ± SD, with each data point representing an individual animal. Significance was calculated using a Mann Witney non-parametric test. n.s.: not significant. Scale bars in **A**: 20μm (top and middle rows); 10μm (bottom row).

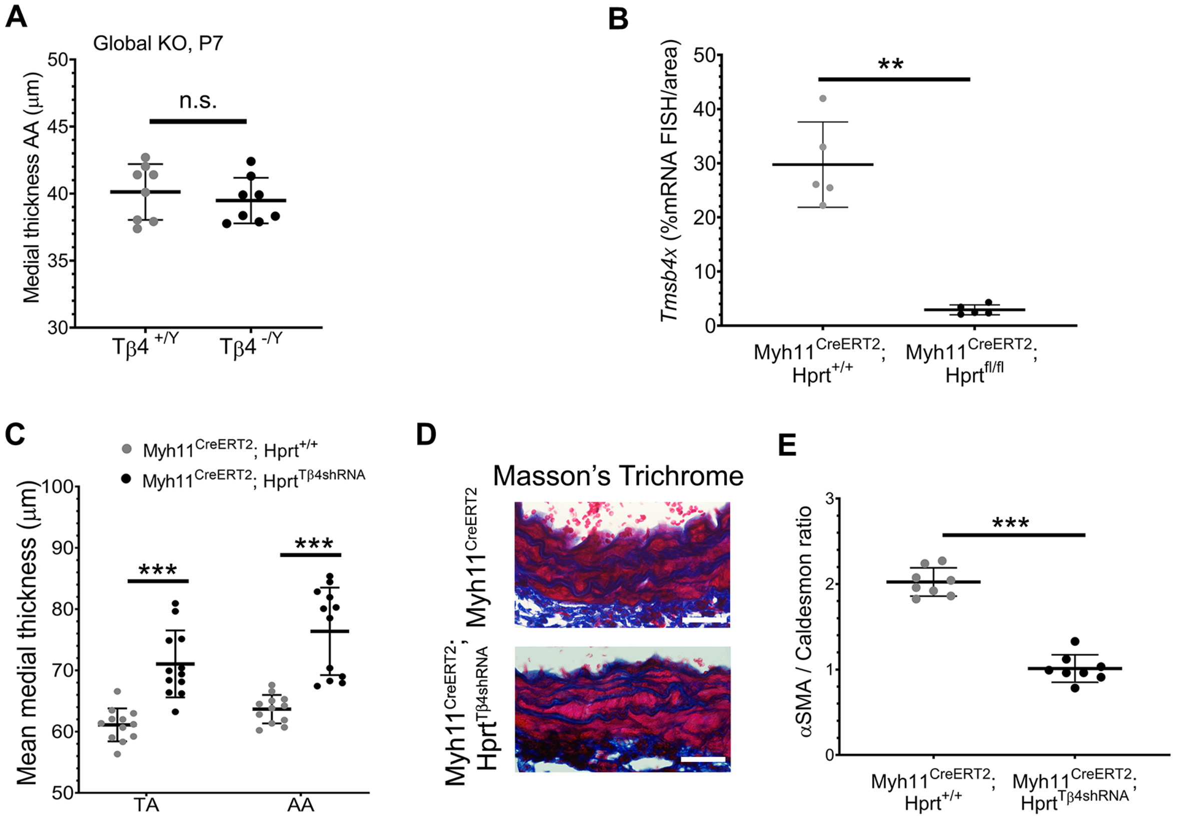


**Supplemental Figure 4. A postnatal, smooth muscle cell-autonomous requirement for Tβ4 for maintenance of healthy aorta (in conjunction with Figure 2).** Medial thickness of global KO and control aortas in postnatal day (P)7 male mice (**A**). *Tmsb4x* was deleted from medial VSMCs of 3 week old Myh11$^{CreERT2}$; Hprt$^{T\beta4shRNA}$ knockdown mice; RNAScope for *Tmsb4x* mRNA, compared with Myh11$^{CreERT2}$; Hprt$^{+/+}$ control mice (shown in Figure 2D), quantified in **B**. Medial thickness in 12-week old Myh11$^{CreERT2}$; Hprt$^{T\beta4shRNA}$ knockdown mice, compared with Myh11$^{CreERT2}$; Hprt$^{+/+}$ control mice mice (**C**). Masson's Trichrome staining to examine collagen deposition (**D**). Ratio of contractile/synthetic VSMC markers (αSMA/Caldesmon, respectively) in Myh11$^{CreERT2}$; Hprt$^{T\beta4shRNA}$, compared with Myh11$^{CreERT2}$;Hprt$^{+/+}$ aortas, quantification (**D**) of immuno-fluorescence in Figure 2I. Data are presented as mean ± SD, with each data point representing an individual animal. Significance was calculated using a Mann Witney non-parametric test (**A**, **B**, **E**) or two-tailed unpaired Student's t-tests (**C**; with Holm-Sidak correction for multiple comparisons). n.s. = not significant; ** $P \leq 0.01$; *** $P \leq 0.001$. Scale bars: **D**: 50µm.

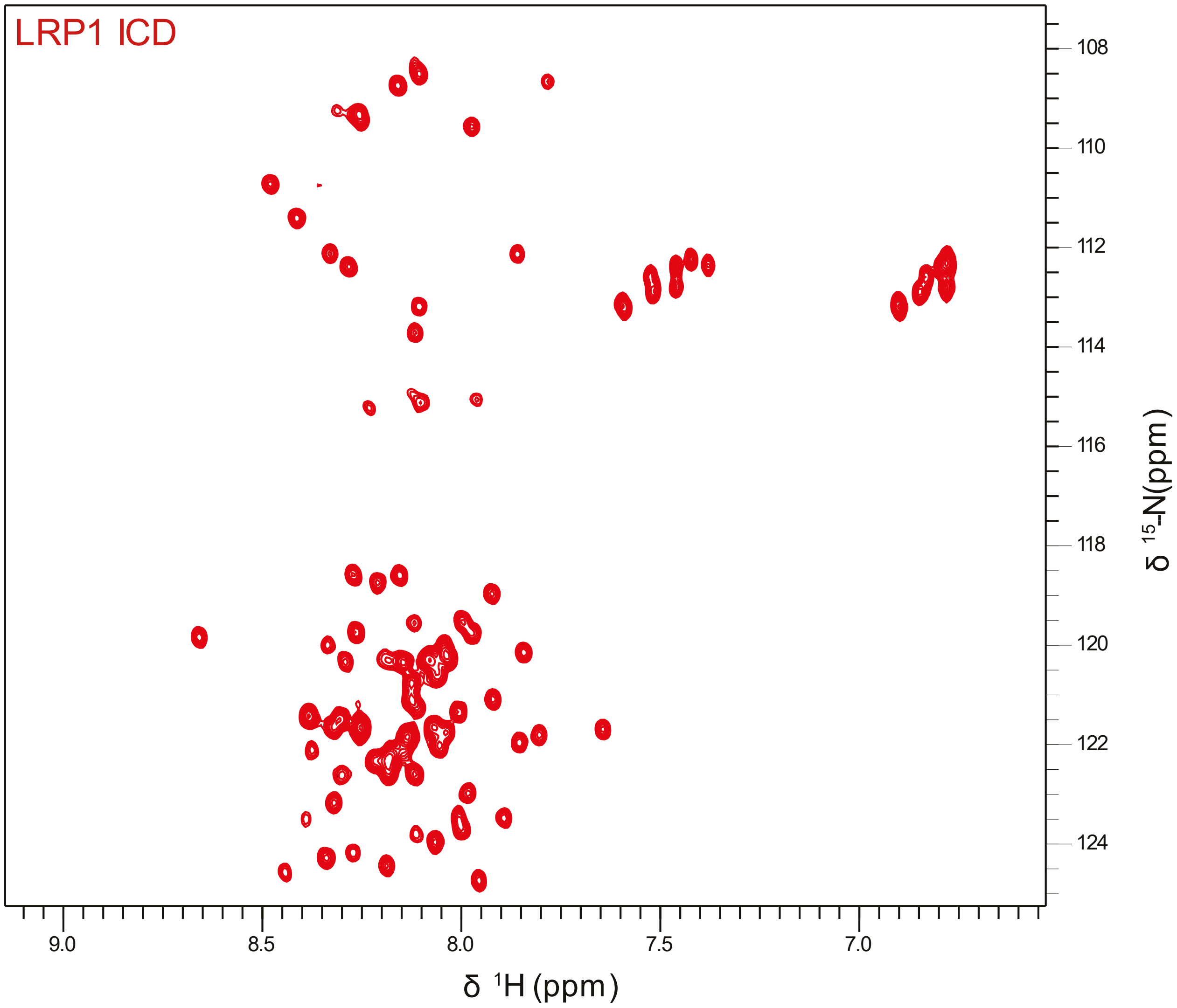


**Supplemental Figure 5. $^{1}H$ $^{15}N$ HSQC NMR spectra of $^{15}N$-labeled intracellular domain (ICD) of LRP1.**

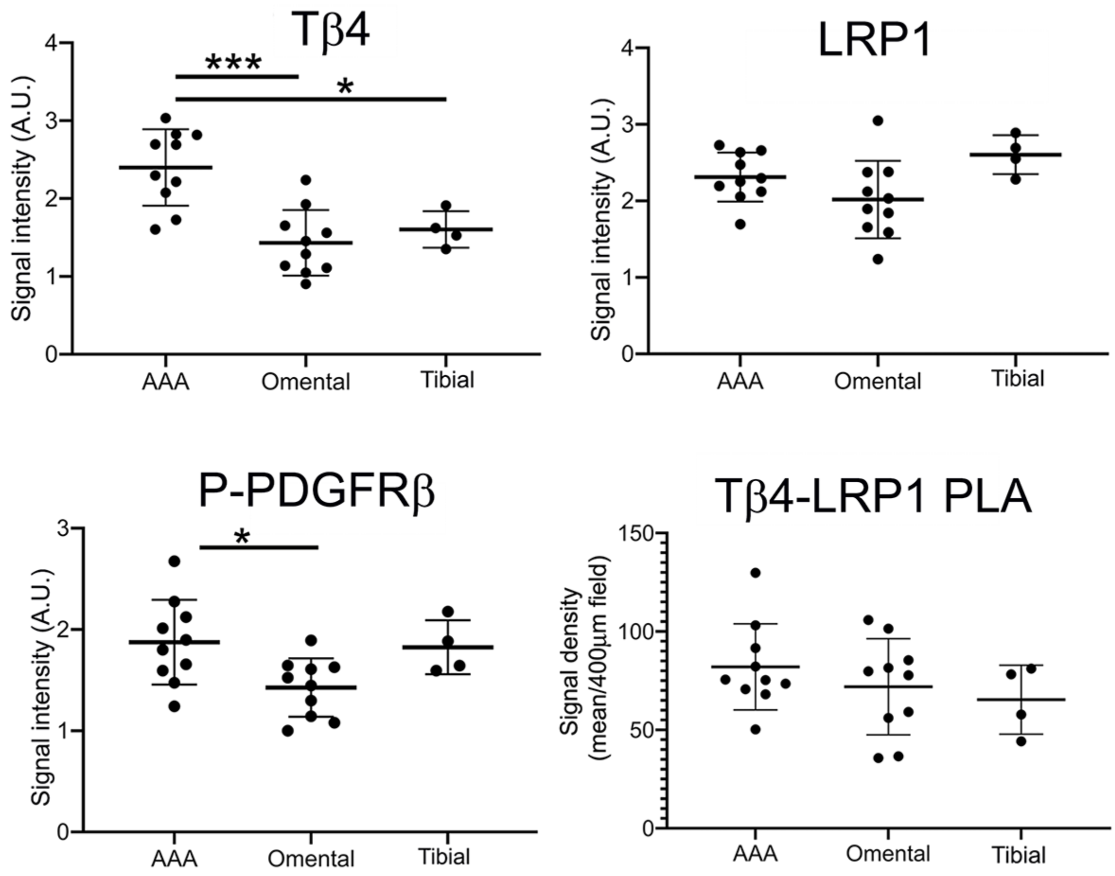


**Supplemental Figure 6. Tβ4 interacts with Low density lipoprotein receptor related protein 1 (LRP1) in human arterial smooth muscle cells (in conjunction with Figure 4).** Quantification of Tβ4 and LRP1 expression and PDGFRβ activation (phosphorylation at Tyr1021) in human aorta from AAA patients and matched omental artery from the same patients (n=10) and in human tibial arteries (n=4). Expression was determined by immunofluorescence, shown in Figure 4A,C. Association of Tβ4 and LRP1 was assessed across these arteries by proximity ligation assay (PLA; shown in Figure 4D). Significance was calculated using a one-way ANOVA with Tukey's multiple comparison tests * $P \leq 0.05$; *** $P \leq 0.001$. AAA: abdominal aortic aneurysm; PLA: proximity ligation assay; int: intima; med: media; adv; adventitia.

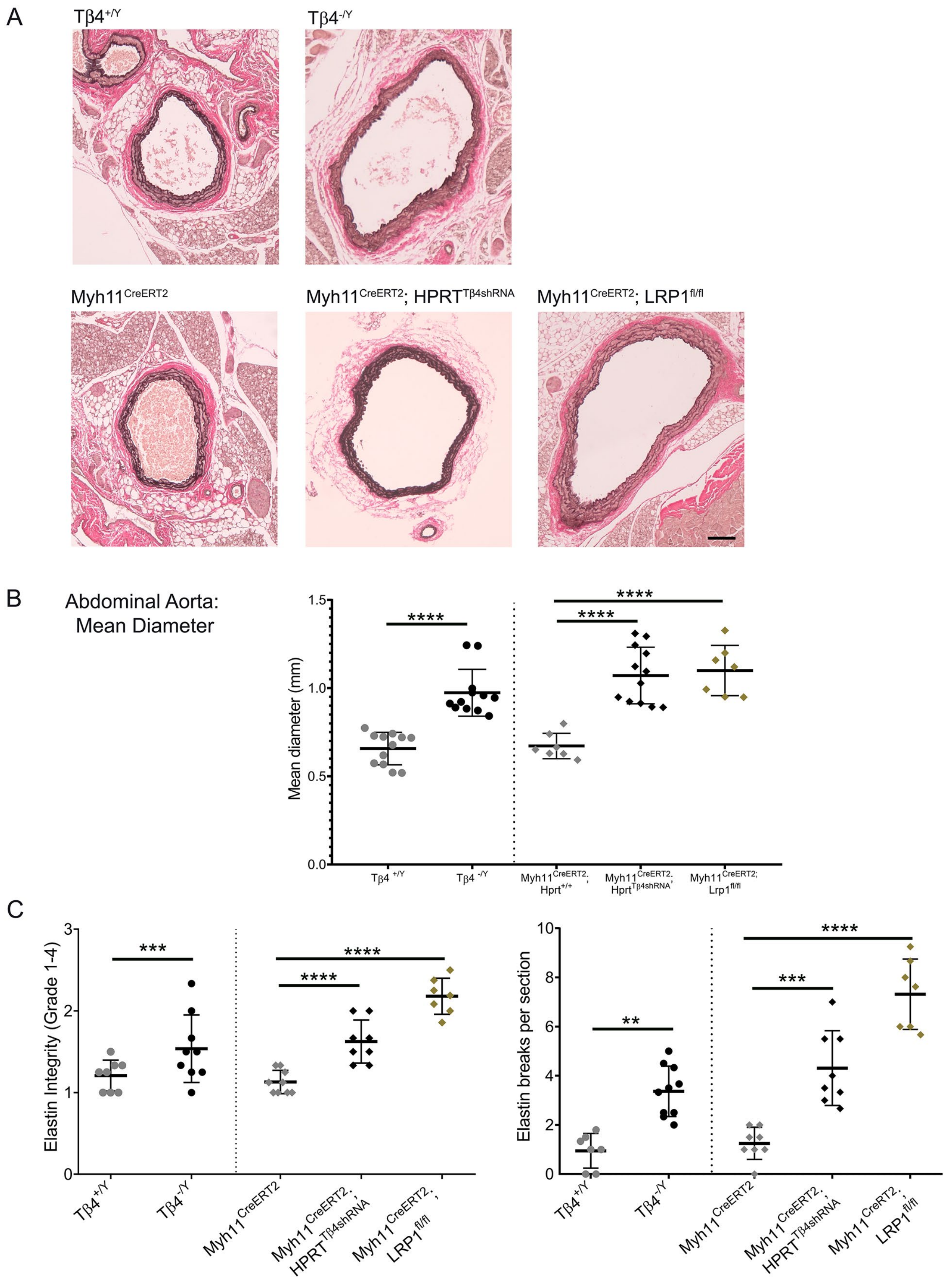


**Supplemental Figure 7. Global and VSMC-specific loss of Tβ4 phenocopies VSMC-specific *Lrp1* mutants.** Baseline phenotypes of $T\beta4^{-/Y}$, compared with $T\beta4^{+/Y}$, and $Myh11^{CreERT2}$; $Hprt^{T\beta4shRNA}$ and $Myh11^{CreERT2}$; $Lrp1^{fl/fl}$, compared with $Myh11^{CreERT2}$, revealed by Verhoeff-van Gieson staining (**A**), quantification of aortic diameter (**B**) and elastin integrity, quantified both by an elastin damage score and number of breaks per section (**C**). Significance was calculated using one-Way ANOVA with Tukey's multiple comparison tests (**B-C**). **p≤0.01; ***: p≤0.001; ****p≤ 0.0001. Scale bar: **A**: 100μm, applies to all images.

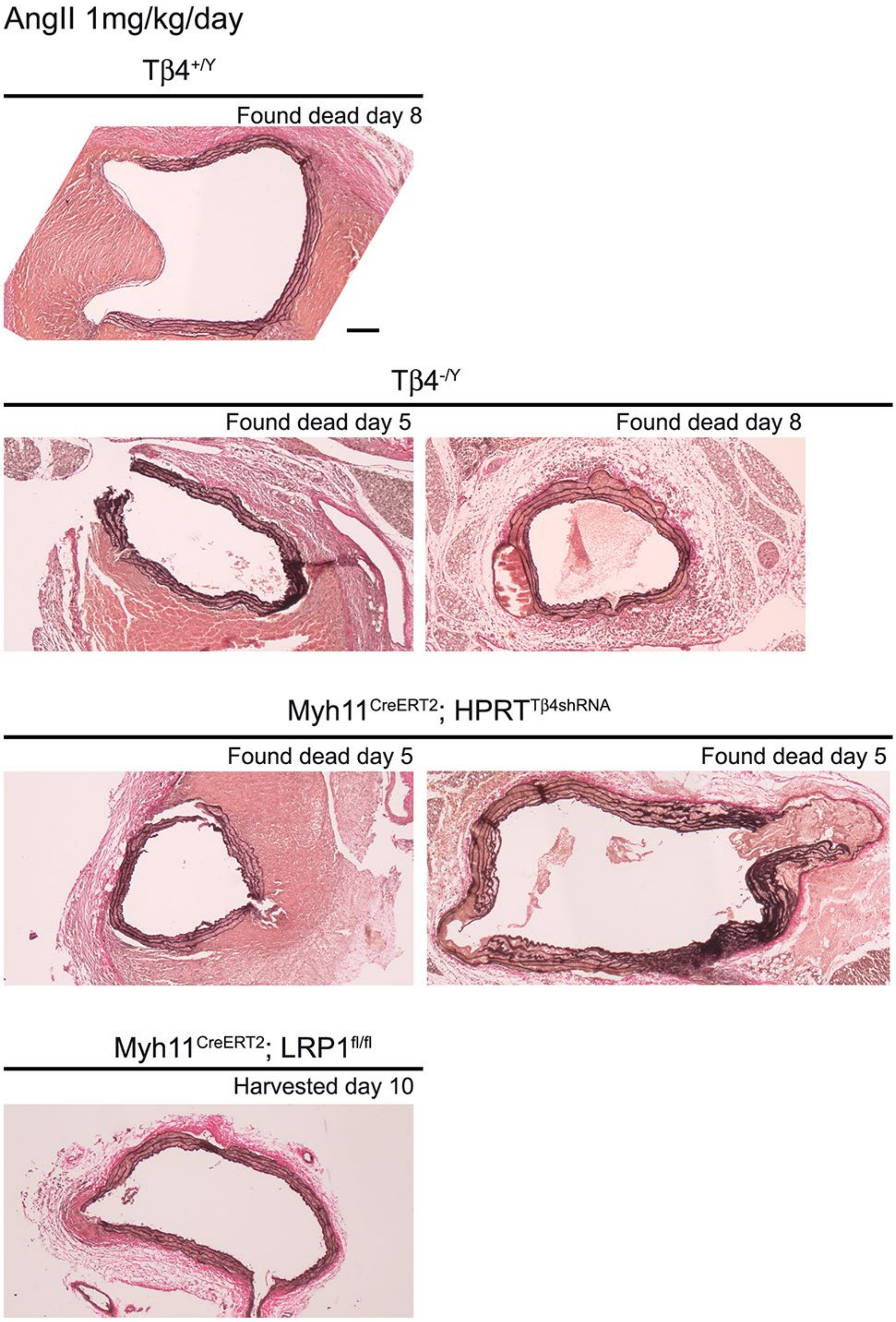


**Supplemental Figure 8. Representative examples of aortic rupture in AngII-infused mice.** A higher incidence of rupture and dissection were observed in mice lacking Tβ4 and, overall, they occurred earlier, with severe disruption of elastin lamellae and aortic dilatation. These samples could not be used for assessment of aortic diameter or elastin integrity (Figure 5) as the animals died prior to the experimental end point at day 10. Most deaths were due to aortic rupture, although occasionally dissection was detected in the same aorta, an example of which is shown for $T\beta4^{-/Y}$ (day 8). Scale bar: 200μm, applies to all images.

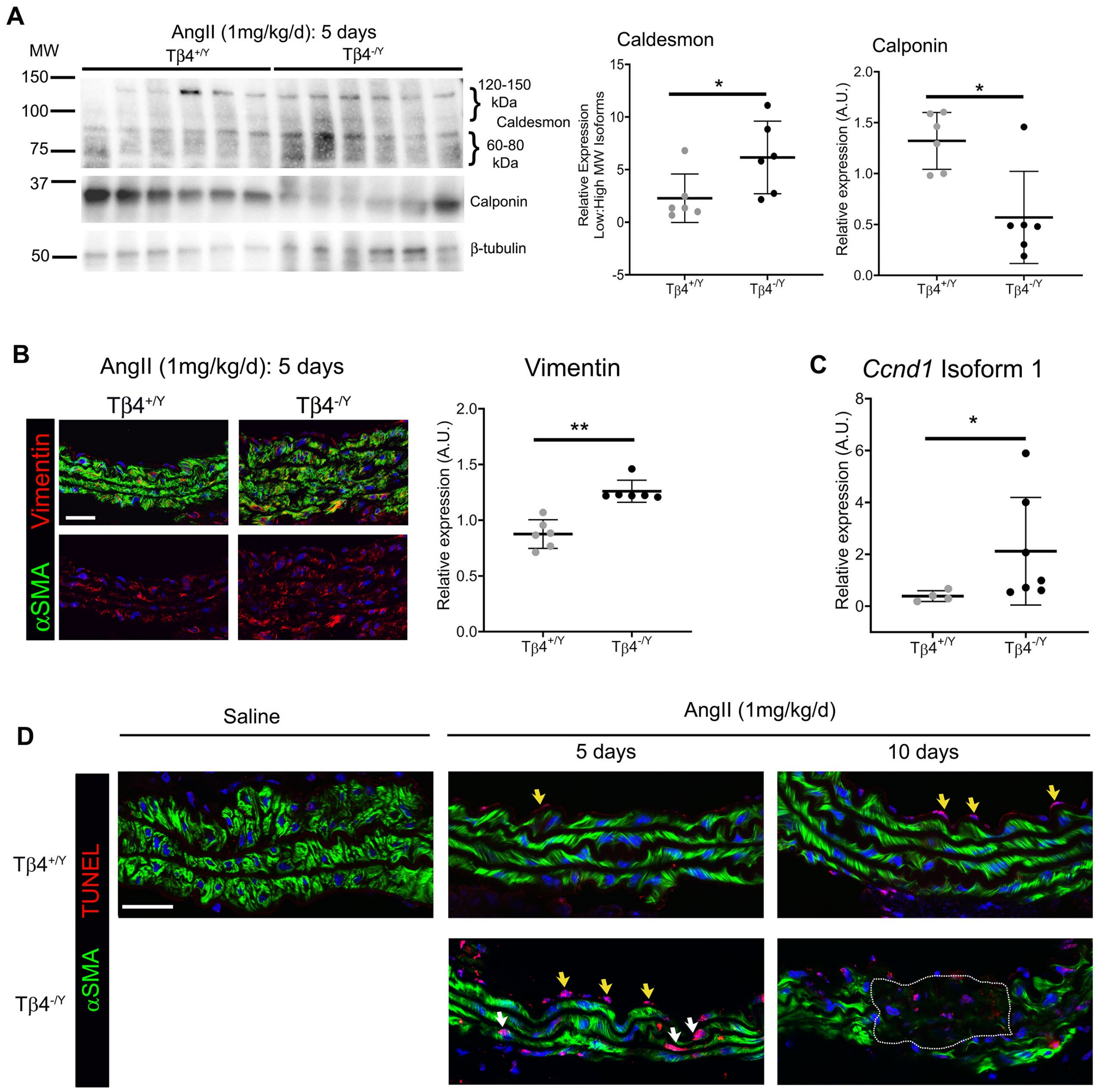


**Supplemental Figure 9. Mice lacking Tβ4 display more severe aneurysmal phenotypes, associated with augmented VSMC phenotypic switching and an apparent increase in apoptosis (in conjunction with Figure 5).** Quantification of high and low molecular weight Caldesmon isoforms and contractile marker Calponin by western blotting after 5 days' AngII infusion (**A**). Immunofluorescence to assess medial layer morphology and VSMC phenotype, with the synthetic marker vimentin along with contractile marker, αSMA (**B**). Increased expression of the proliferation marker, *Ccnd1*, was detected in some Tβ4$^{-/Y}$ aortas, compared with controls (qRT-PCR, **C**). TUNEL+ apoptotic cells were rarely detected within the medial layer; most aortas lacked TUNEL+ VSMCs, thus these images could not be described as representative. Endothelial (yellow arrows) and adventitial cells were more frequently TUNEL+ than VSCMs in this model (**D**). Data are presented as mean ± SD, with each data point representing an individual animal. Significance was calculated using a Mann Witney non-parametric test. * P ≤ 0.05; ** P ≤ 0.01. Scale bars: all 50μm.

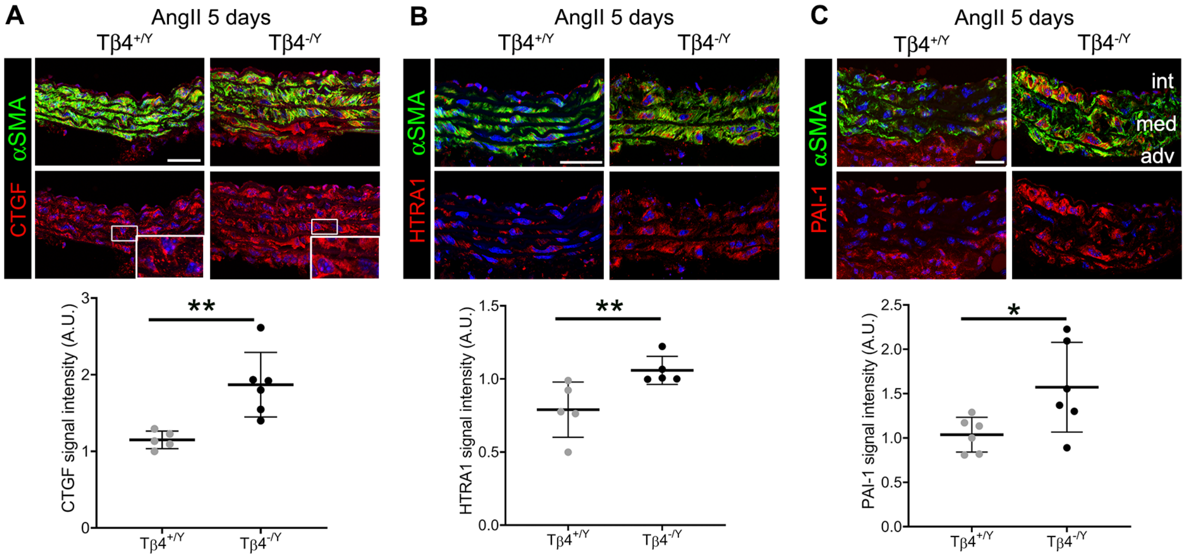


**Supplemental Figure 10. Endocytosis of LRP1 ligands is dysregulated in Tβ4 null aortas.** LRP1 ligands, CTGF (**A**), HTRA1 (**B**) and PAI-1 (**C**) were assessed by immunofluorescence and quantified; representative of n=6. Data are presented as mean ± SD, with each data point representing an individual animal. Significance was calculated using a Mann Witney non-parametric test. * P≤ 0.05; ** P ≤ 0.01. int: intima; med: media; adv; adventitia. Scale bars: all 50μm.

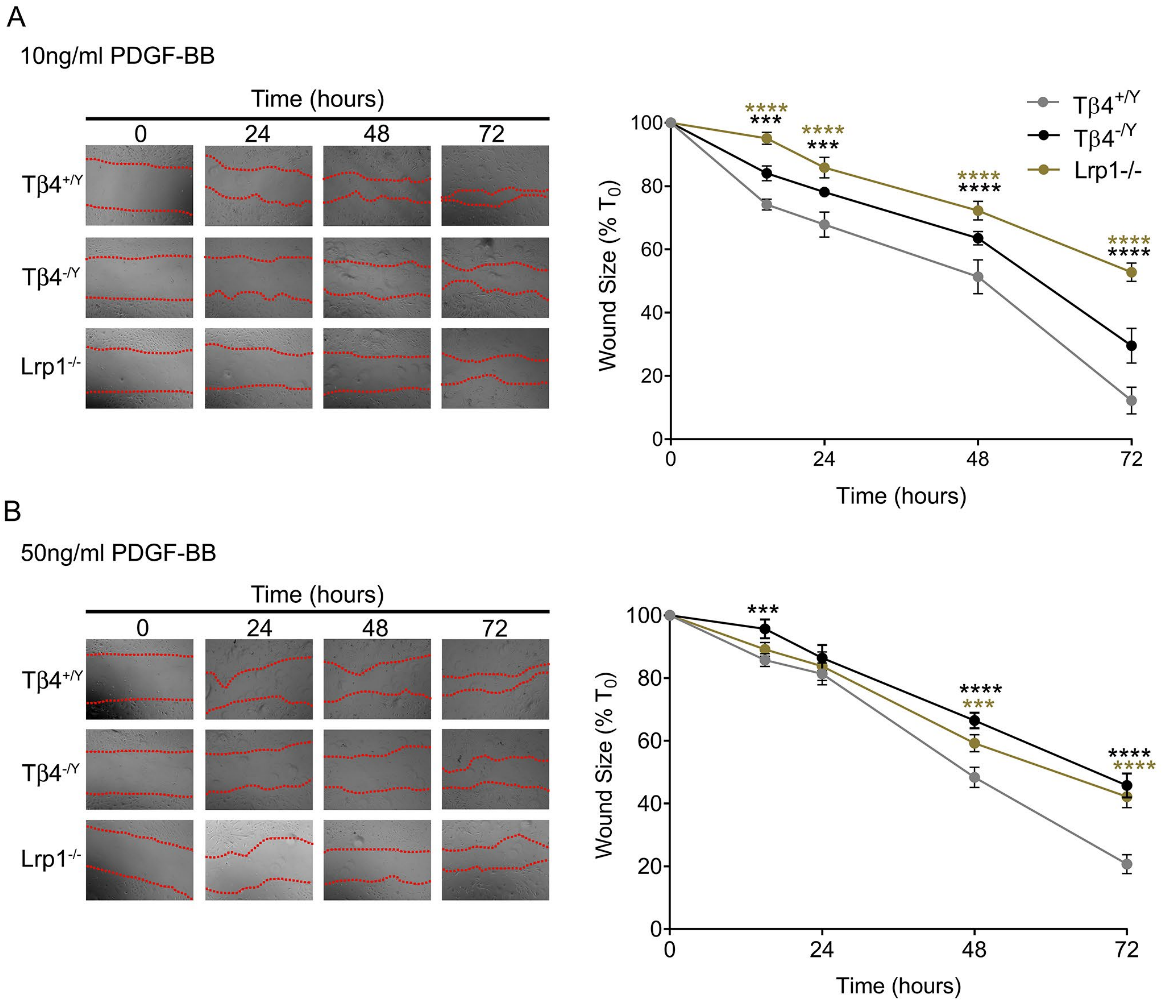


**Supplemental Figure 11. Despite augmented PDGFRβ signaling, Tβ4$^{-/Y}$ and Lrp1$^{-/-}$ aortic VSMCs migration rates were reduced, compared with control cells.**

Migration rates were determined by scratch wound assay for Tβ4$^{+/Y}$, Tβ4$^{-/Y}$ and Lrp1$^{-/-}$ aortic VSMCs, in response to 10ng/ml (**A**) or 50ng/ml (**B**) PDGF-BB. Representative images of same region of well at 0, 24, 48 and 72 hours after wounding. Data are presented as mean ± SEM from n=3 experiments, each from an independent VSMC isolation. Significance was calculated using two-way ANOVA with Dunnett's post hoc tests. ***: p≤0.001; ****p≤ 0.0001.

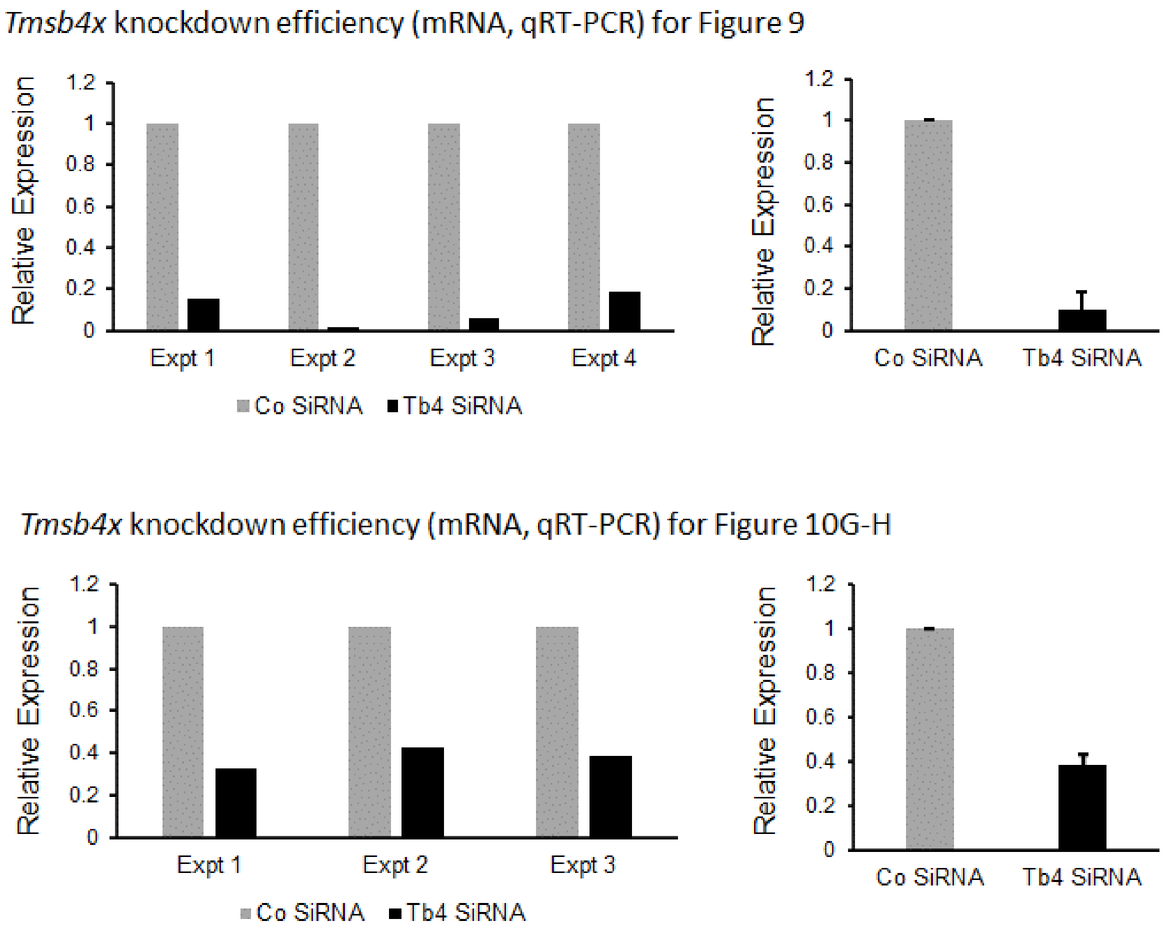


**Supplemental Figure 12. siRNA-mediated Tβ4 knockdown**. *Tmsb4x* Knockdown was assessed by qRT-PCR. A minimum of 85% knockdown by qPCR was set as the criterion for inclusion in the experiments in Figure 8. Knockdown data are shown for each of the individual experiments (n=3/4) and the mean qRT-PCR data.

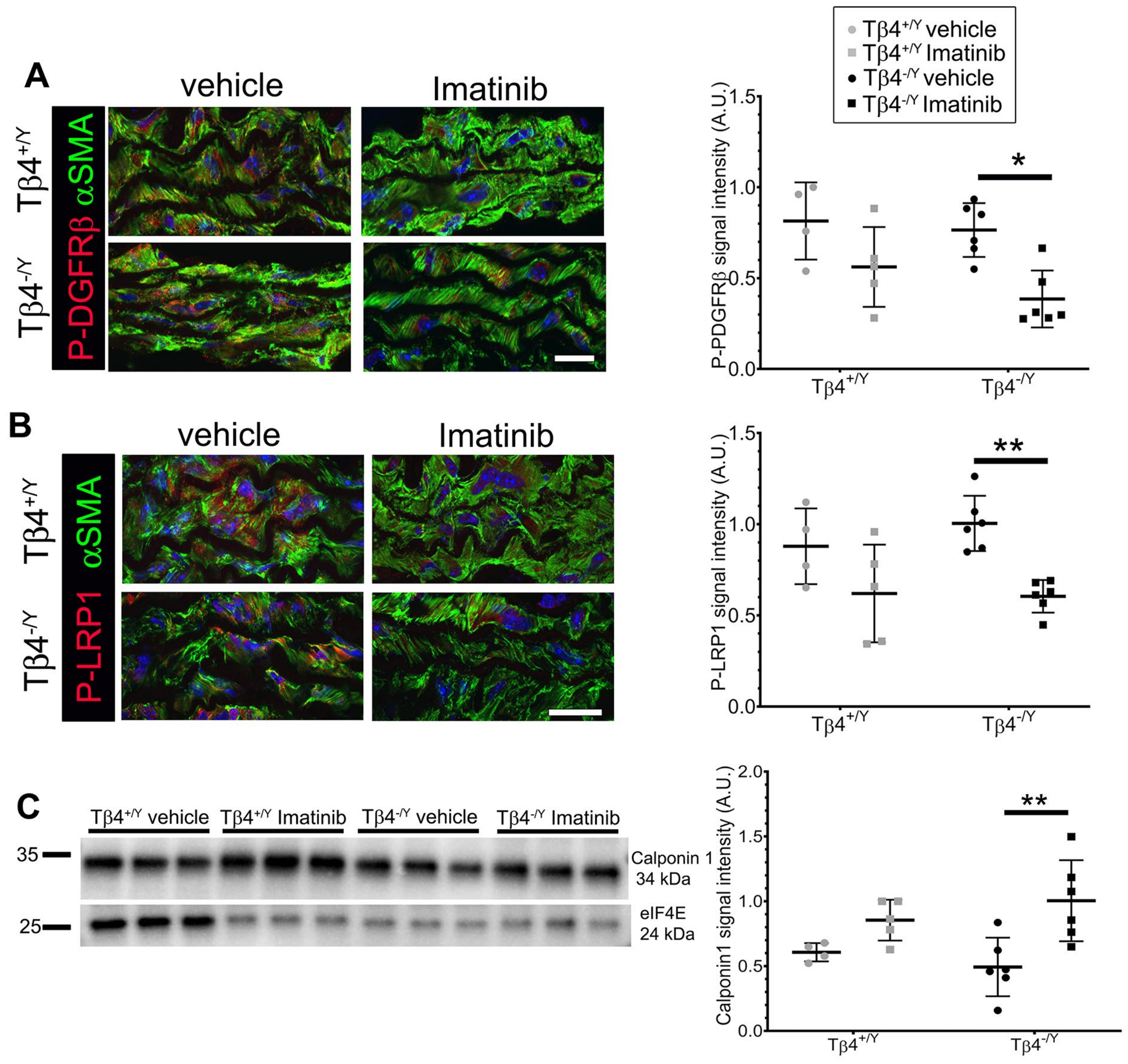


**Supplemental Figure 13. Restoration of normal PDGFRβ signaling ameliorates aneurysmal phenotype of Tβ4 null mice (in conjunction with Figure 11).** Imatinib attenuated PDGFRβ pathway activity in aortas of AngII-infused Tβ4$^{+/Y}$ and Tβ4$^{-/Y}$ mice, confirmed by immunostaining for phospho-LRP1 (Tyr 4507; **A**) and phospho-PDGFRβ (Tyr1021; **B**). Western blotting of Calponin1 (contractile marker; **C**), to further assess VSMC phenotype. All samples were harvested after 8 days' AngII infusion. Data are presented as mean ± SD, with each data point representing an individual animal. Significance was calculated using two-way ANOVA with Tukey's multiple comparison tests. * P≤ 0.05; ** P ≤ 0.01. Scale bars: **A**, **B**: 50μm.

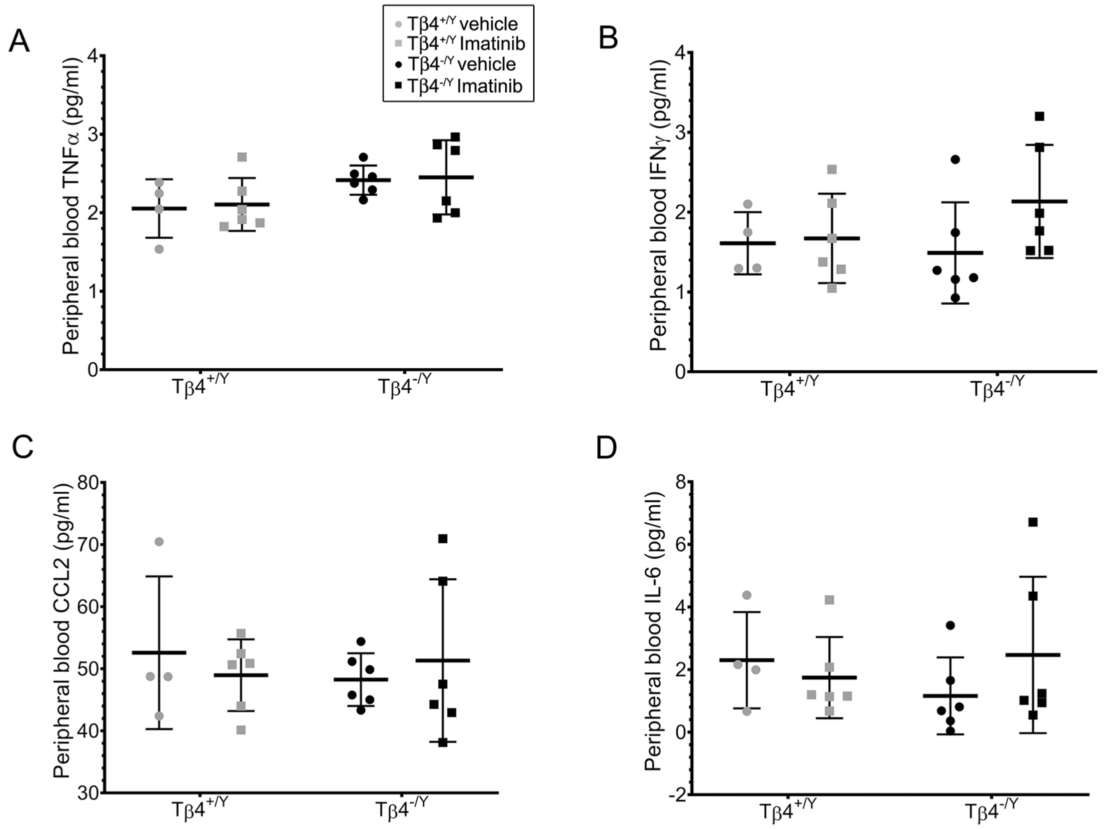


**Supplemental Figure 14. Pro-inflammatory cytokines are unaltered by Imatinib treatment in the AngII model of aortic aneurysm**. Serum from $T\beta4^{+/Y}$ and $T\beta4^{-/Y}$ mice were assayed by multiplexed automated ELISA for TNFα (**A**), IFNγ (**B**) CCL2 (**C**) and IL-6 (**D**) levels after 8 days' AngII infusion. Cytokine levels were not significantly altered regardless of whether mice received Imatinib or vehicle, nor were they altered by genotype (consistent with data in Figure 6). Significance was calculated using two-way ANOVA with Tukey's post hoc tests.

## Supplemental Table 1. Yeast 2 Hybrid screen: Putative interactors

| Gene | | No. clones |
|---|---|---|
| Ataxin-8 | *Atxn8* | 1 |
| ATP-binding cassette, sub-family B (MDR/TAP), member 6 | *Abcg6* | 1 |
| Basigin precursor | *Bsg* | 1 |
| Circadian locomotor output cycles protein kaput | *Clock* | 1 |
| Cleavage and polyadenylation specific factor 6 | *Cpsf6* | 1 |
| DET1 and DDB1 associated 1 | *Dda1* | 1 |
| Dihydrolipoamide branched chain transacylase E2 | *Dbt* | 1 |
| Eukaryotic translation elongation factor 2 | *Eef2* | 1 |
| EMI domain containing 2 | *Emid2* | 1 |
| Frizzled-8 precursor | *Fzd8* | 1 |
| Gasdermin-A3 | *Gsdma3* | 2 |
| Guanidinoacetate methyltransferase | *Gamt* | 1 |
| Guanine nucleotide-binding protein G(q) subunit alpha | *Gnaq* | 3 |
| Glypican 1 | *Gpc1* | 1 |
| High density lipoprotein (HDL) binding protein | *Hdlbp* | 1 |
| INO80 complex subunit D isoform 1 | *Ino80d* | 1 |
| Iroquois-class homeodomain protein IRX-1 | *Irx1* | 1 |
| Iroquois-class homeodomain protein IRX-2 | *Irx2* | 1 |
| Low density lipoprotein receptor-related protein 1 | *Lrp1* | 1 |
| Leucine-rich repeat-containing protein 3C precursor | *Lrrc3c* | 2 |
| Leucine-rich repeat-containing protein 39 isoform 2 | *Lrrc39* | 1 |
| Microfibrillar-associated protein 2 | *Mfap2* | 2 |
| Necdin | *Ndn* | 1 |
| Neuropilin-2 | *Nrp2* | 1 |
| Polyadenylate-binding protein 4-like isoform X | *Pabpc4l* | 1 |
| Potassium large conductance calcium-activated channel | *Kcnmb4* | 1 |
| Protocadherin 18 precursor | *Pcdh18* | 1 |
| Ribosomal protein L37 | *Rpl37* | 1 |
| Rho family GTPase 2 | *Rnd2* | 2 |
| Scaffold attachment factor B2 | *Safb2* | 1 |
| Serine/arginine-rich splicing factor 3 | *Srsf3* | 3 |
| Serine/threonine-protein kinase 38 isoform | *Stk38* | 2 |
| SHC (Src homology 2 doman containing)-transforming protein 4 | *Shc4* | 1 |
| SNW domain containing 1 | *Snw1* | 1 |
| Transcription factor SOX-5 | *Sox5* | 1 |
| Spectrin beta 2 | *Spnb2* | 1 |
| Stathmin 1 | *Stmn1* | 5 |
| SWI/SNF-related, matrix associated, actin dependent regulator of chromatin, subfamily a, member 2 | *Smarca2* | 5 |
| Ubiquitin-conjugating enzyme E2 N | *Ube2n* | 1 |
| Uncharacterized protein CXorf49 homolog | | 2 |
| Von Hippel-Lindau (VHL) binding protein 1 | *Vbp1* | 1 |
| WW domain containing adaptor with coiled-coil region | *Wac* | 1 |
| Genomic flanking Yamaguchi sarcoma viral (v-yes) oncogene homolog 1 | *Yes1* | 1 |

**Supplemental Methods**

***Animal models.*** Mice were housed and maintained in a controlled environment and all procedures involving the use and care of animals were performed in accordance with the Animals (Scientific Procedures) Act 1986 (Home Office, United Kingdom) and approved by the University of Oxford or University College London Animal Welfare and Ethical Review Boards. All mice used for the study were maintained on the C57Bl6/J background for at least 10 generations. The global Tβ4 Knockout Tβ4(-/Y) strain, in which exon 2 of *Tmsb4x* locus is deleted, were a kind gift of Martin Turner, Babraham Institute and have been previously described(1). VSMC-specific Tβ4 knockdown mice were generated by crossing the previously described *Hprt1*-targeted, floxed Tβ4 shRNA line(1, 2) with tamoxifen-inducible Myh11$^{CreERT2}$(3), to generate Myh11$^{CreERT2}$; Hprt$^{T\beta4shRNA}$. VSMC-specific Lrp1 knockouts were generated by crossing the previously described floxed Lrp1 line ((4); obtained from Jax) to homozygosity with Myh11$^{CreERT2}$(3), to generate Myh11$^{CreERT2}$; Lrp1$^{fl/fl}$. Conditional knockdown of Tβ4 or deletion of *Lrp1* were induced by oral gavage of 3 week old mice with 3 doses of tamoxifen (80mg/kg) on alternate days. Vascular injury studies were designed and executed in line with the recommendations of the American Heart Association Council on Arteriosclerosis, Thrombosis and Vascular Biology; and Council on Basic Cardiovascular Sciences(5).

***In vivo blood pressure measurement.*** One day after the MRI scan (exactly as previously described(6)), animals were anaesthetised with isoflurane (4%) and placed in supine position onto a heating plate with feedback control (Physitemp Instruments, NJ). Animals were kept at 37±1°C while oxygen and anaesthetics (1-2% isoflurane) were supplied via a nose cone (1 L/min). Body temperature and heart rate were recorded for the whole experiment using PowerLab with Chart 5 (ADInstruments, UK). A T-shaped middle-neck incision from mandible to the sternum was made. Blunt dissection was used to expose the left common carotid artery. A 5.0 Mersilk (Ethicon, NJ) suture was used to tie off the distal end of the artery while a micro vascular clip was used to occlude the proximal end of the exposed artery. A small incision near the distal end of the artery was made and a fluid filled tube (heparin; 100 U/mL diluted in 0.9% saline) with an inner diameter of 0.28 mm (Critchley Electrical Products Pty Ltd, Australia) was introduced into the artery and fixed in place with a suture. Following that, the micro vascular clip was removed and the arterial pressure was recorded using the MLT844 pressure transducer (ADInstruments, UK). After stabilisation of the signal for about 5-10 min an average of 1000 heart beats was used for analysis.

***Aneurysm model.*** 8-12 week old adult mice were infused with 1mg/kg/day Angiotensin II (Sigma) or saline by subcutaneous implantation of an osmotic mini pump (Alzet) for 10 days. Animals were regularly monitored and weighed post-surgery until harvest. For the rescue experiment, 12-week old male Tβ4$^{-/Y}$ and Tβ4$^{+/Y}$ mice were randomly assigned into groups to be orally gavaged with either 10 mg/kg Imatinib mesylate or sterile water, daily for a period of 10 days, starting two days prior to AngII mini pump implantation.

**Multiplexed automated cytokine ELISA** Peripheral blood was collected at harvest by cardiac puncture, placed into chilled microcentrifuge tubes coated with 0.5M EDTA and kept on ice. Samples were centrifuged at 1500 x g for 15 minutes and plasma supernatant collected into a fresh tube and stored at -80ºC. Using 25μl serum per assay, cytokine levels were quantified by multianalyte automated ELISA (*Ella* technology from ProteinSimple).

***Human tissue sampling.*** Subjects undergoing open abdominal aortic aneurysm repair were prospectively recruited from the Oxford Abdominal Aortic Aneurysm (OxAAA) study. The study was approved by the Oxford regional ethics committee (Ethics Reference: 13/SC/0250). All subjects gave written informed consent prior to the study procedure. Baseline characteristics

of each participant were recorded. During surgery to repair the AAA, a wedge of abdominal omentum containing a segment of omental artery was identified and biopsied en bloc. Isolation of omental artery was performed immediately in the operating theatre. The omental artery segment was cleared of peri-vascular tissue and snapped frozen. Prior to incision of the aortic aneurysm, a marker pen was used to denote the cross section of maximal dilatation according visual inspection. A longitudinal strip of the aneurysm wall along the incision was then excised. The aneurysm tissue was stripped of the peri-vascular tissue and mural thrombus. The tissue at the maximal dilatation was isolated, divided into smaller segments, and snap frozen for subsequent analysis. As a comparison to aneurysm tissue, non-aneurysmal lower limb arteries were obtained from patients who required a major lower limb amputation as treatment for limb ischaemia. Written consent was obtained from each patient at the time of surgery for the retrieval of a small segment of tibial artery (from the amputated limb stump) for research analysis.

***Histological Sample Preparation.*** For histological and immunohistochemical analyses, mouse aortic sinus, aortic arch and descending aorta were harvested, washed in phosphate-buffered saline (PBS; pH7.4) and fixed in 4% paraformaldehyde (PFA) at room temperature (RT), either for two hours (cryosectioning) or overnight (paraffin embedding). For histological analysis, samples were dehydrated using a series of graded ethanol concentrations (50-100%, each for at least 2 hours), and cleared in butanol overnight. Samples were incubated at 60°C in 1:1 butanol: molten pastillated fibrowax for 30 minutes, then three times in 100% molten wax, each for 4 hours to overnight, before embedding and sectioning (10 μm transverse sections). Paraffin sections were stained with Hematoxylin and Eosin, Elastic stain kit (Verhoeff van Gieson) and Masson's Trichrome kit (all from *Sigma-Aldrich*), each according to the manufacturer's protocol. After imaging, morphology was assessed on sections which had been anonymised and blinded for genotype. Elastin breaks per section were counted manually. Aortic diameters were measured using ImageJ. Elastin breaks/score and aortic diameter are all expressed as the mean of 6 sections per aorta. For immunohistochemical analyses, fixed tissues were rinsed in PBS and equilibrated in 30% sucrose (in PBS) overnight at 4°C. Samples were placed in 50:50 (30% sucrose: Tissue-Tek OCT), then embedded in OCT and chilled at -80°C.

***Flow Cytometric Analysis of Infiltrating Immune Cells.*** Before collection of the aorta, the mouse was transcardially perfused with phosphate-buffered saline (PBS; pH7.4) to wash and remove peripheral blood. The descending aorta, cleaned of adipose tissue, was cut into 4 pieces, then enzymatically dissociated using the Multi Tissue Dissociation kit 2 (Miltenyi Biotec) and gentleMACS Octo Dissociator with heaters according to manufacturer's instructions. Red blood cells were lysed, and cell counts were determined for each aorta. The cell suspension was stained with Zombie Violet Fixable Viability Dye (1:500; Biolegend) for 15 min at RT, followed by pre-treatment with anti-CD16/32 (1:50; Biolegend) for 5 min at RT to block Fcγ receptors, and surface staining with PeCy7 anti-CD45 (1:200; BD Biosciences; 30-F11), FITC anti-CD11b (1:200; BD Biosciences; M1/70), PE anti-CD14 (1:100; Life Technologies; Sa2-8) and APC anti-CCR2 (1:10; R&D systems; 475301) antibody for 30 min on ice. Stained cells were fixed (BD CellFIX) and analysed the next day on BD LSRFortessa X20 cytometer (BD biosciences). Flow cytometry data were analysed using FlowJo software.

***Immunofluorescence Staining on Cryosections.*** Frozen sections of descending aorta or aortic sinus (base of the heart) were cut at a thickness of 10 µm, air-dried for 5 minutes and rinsed in PBS. Sections were permeabilized in 0.5% Triton-X100/PBS for 10 minutes, rinsed in PBS and blocked for 1-2 hours (1% BSA, 10% goat serum or donkey serum; 0.1% Triton-X100 in PBS). Sections were incubated with primary antibodies (diluted as below) at 4°C overnight. Sections were washed 5-6 times in 0.1% Triton-X100 in PBS (PBST) then incubated

in secondary antibody for 1 hour at RT. Sections were washed 3 times in PBST and, incubated with 300 nmol/μL 4',6-diamidino-2-phenylindole (DAPI) in PBS for 5 minutes and rinsed a further twice in PBS. Slides were mounted with 50% glycerol:PBS and imaging was conducted on an Olympus FV1000 confocal microscope or a Leica DM6000 fluorescence microscope. Apoptosis was assessed using the Click-iT TUNEL Alexa Fluor 546 Imaging Assay kit (from ThermoFisher), according to the manufacturers' instructions.

***Cell Culture.*** Primary smooth muscle cells were isolated by enzymatic digestion of murine aortas, as previously described(7). After the first week, fetal bovine serum (FBS) was gradually reduced from 20% to 10% and cells maintained thereafter in DMEM (Gibco) supplemented with 10% FBS, 100 U/ml Penicillin-Streptomycin (Gibco), in a humidified incubator at 37°C and 5% $CO_2$. An immortalised mouse vascular aortic smooth muscle cell line (MOVAS-1) was purchased from ATCC and cultured in DMEM (ATCC) supplemented with 10% FBS, 100 U/ml Penicillin-Streptomycin (Gibco) and 0.2 mg/ml G -418 (Sigma) at 37°C and 5% $CO_2$.

***siRNA treatment.*** MOVAS-1 cells were transfected with 15 nM siRNA against Thymosin β4 (s201860, Thermo Scientific) or control siRNA (Silencer Select negative control, 4390844, Thermo Scientific) using Lipofectamine RNAiMAX (Thermo Scientific) for 16-36 hours. In parallel, siRNA-treated cells were starved in medium without FBS prior to stimulation with 20 ng/ml PDGF-BB for fixed periods of time and processed for Western blot, ELISA or Duolink, as described.

***Antibodies for Immunofluorescence.*** Cy3 conjugated Mouse anti-αSM actin (1:200; Sigma, C6198); FITC conjugated Mouse anti-αSM actin (1:200; Sigma, F3777); Rabbit anti-Vimentin (1:200; Abcam, ab45939); Rat anti-CD68 (1:100; BioRad, MCA1957); Rabbit anti-SM-MHC (1:1000; Abcam, ab125884); Rat anti-Ki67 (1:100; eBioscience, 14-5698); Rabbit anti-Caldesmon (1:250 Abcam, ab32330); Rabbit anti-Thymosin β4 (1:100; Immunodiagnostik, A9520); Mouse anti-LRP1 ICD hybridoma monocolonal antibody (1:25; ATCC CRL-1937); Rabbit anti-phospho-p44/42 MAPK (Erk1/2) (Thr202/Tyr204) (1:200; Cell Signalling Technology, 4370); Rabbit anti-phospho-LRP1 (Tyr4507) (1:100; Santa Cruz Biotechnology, sc-33049); Rabbit anti-phospho-PDGFRβ (Tyr1021) (1:100; Abcam, ab134048); Rabbit anti-phospho JNK1 + JNK2 + JNK3 (T183+T183+T221) (1:100; Abcam, ab124956); Armenian Hamster anti-PECAM1 (1:100; Abcam, ab119341); Cy3-conjugated Rabbit anti-LAMP1 (1:25; Abcam, ab67283), Rat anti-Transferrin receptor (1:25; Abcam, ab22391), Rabbit anti-CTGF(1:200; Abcam, ab6992), Rabbit polyclonal anti-HTRA1(1:200; Abcam, ab38611) and Rabbit anti-PAI-1(1:100; Abcam, ab66705), Rabbit anti-Vimentin(1:250; Abcam, ab45939) Rabbit anti-CD41(1:100; Abcam, ab134131) and Goat IgG anti P-Selectin(1:40; Research and diagnostics, AF737). AlexaFluor 647-conjugated Rabbit anti-EEA1 (1:50; Abcam, ab2900) and AlexaFluor 647-conjugated Rabbit anti-RAB7 (1:100; Abcam, ab198337). AlexaFluor-conjugated secondary antibodies raised against Rat, Rabbit, Mouse or Armenian Hamster IgG (Invitrogen) were used at 1:200.

***Immunoblotting***. SiRNA-treated and PDGFβ-stimulated cells were lysed in RIPA buffer (50 mM Tris-HCl pH7.5, 100 mM NaCl, 1% NP-40, 0.1% SDS, 0.5% sodium deoxycholate, PhosSTOP and cOmplete protease inhibitor cocktail (Roche)). Western blots were performed using a standard protocol. Proteins were transferred by semi-dry transfer onto PVDF membrane. After blocking, membranes were probed with primary antibodies overnight. A modified protocol was used for Thymosin β4 Western blotting(8). Proteins were separated on Tris-Tricine gels and cross-linked with 10% glutaraldehyde, before wet overnight transfer onto nitrocellulose membrane. Membranes were crosslinked with UV light (254 nm, 10 min) before blocking and overnight antibody probing. Antibodies used were Rabbit anti-phospho-PDGFRβ (Tyr1021) (1:500; Abcam, ab62437), Rabbit anti-PDGFRβ (1:1000; Abcam, ab32570), Rabbit

anti-LRP1 (1:10,000; Abcam, ab92544), Rabbit anti-phospho-Akt (S473) (1:500; Cell Signalling Technology, 4058), Rabbit anti-phospho-ERK1/2 (Thr202/Tyr204) (1:1000; Cell Signalling Technology, 9101), Rabbit anti-Calponin1(EP789Y)(1:400; Abcam,ab46794), Rabbit anti-Caldesmon (1:250 Abcam; ab32330), Rabbit βTubulin-HRP conjugated(9F3)(1:300; Cell Signalling Technology, 5346), Rabbit anti-AKT( 1: 250; Cell Signalling Technology, 9272), Rabbit anti-p44/42 (ERK1/2) MAPK(137F5)( 1:500; Cell Signalling Technology, 4695), Rabbit Anti-Filamin A antibody (EP2405Y) (1:500; Abcam, ab76289), Rabbit anti-eIF4E (1:1000; Cell Signalling Technology, 2067), Rabbit anti-GAPDH (1:1000; Proteintech, HRP-60004) and Sheep anti-Thymosin β4 (1:100; R&D, AF6796). HRP-conjugated secondary antibodies were from GE Healthcare (rabbit, NA934) and R&D (sheep, HAF016).

***Duolink® Proximity Ligation Assay***. Aortic sections, primary VSMCs or SiRNA-treated and PDGF-BB-stimulated MOVAS-1 cells were fixed with 4% PFA and permeabilised with 0.1% Tween-20. To assess proximity, the following antibody combinations were used: Mouse anti-LRP1 (1:100; Abcam, ab28320) and either Rabbit anti-PDGFRβ (1:100; Abcam, ab32570) or Rabbit anti-Thymosin β4 (1:100; Immundiagnostik, aa1-14). Duolink® In Situ PLA Rabbit Plus and Mouse Minus probes were used in combination with Detection Reagent Green (Sigma) following manufacture's protocol. Following Duolink® protocol, cells were co-stained for smooth muscle markers or endocytic compartments using the antibodies described above. Colocalisation was determined, using the ImageJ plug-in JACOP 2.0(9).

***Surface biotinylation ELISA***. SiRNA-treated and PDGF-BB-stimulated cells were washed twice with cold PBS and surface proteins were biotinylated with 0.5 mg/ml Sulfo-NHS-SS-Biotin (Thermo Scientific) for 30 min on ice. The reaction was quenched with 15 mM glycine (2x 5 min) and cells were lysed in GST lysis buffer (10% glycerol, 50 mM Tris-HCl pH7.5, 200 mM NaCl, 1% NP-40, 2 mM $MgCl_2$, PhosSTOP and cOmplete protease inhibitor cocktail (Roche)). Nunc-Immuno™ MicroWell™ 96 well plates were coated with antibody in 0.05 M $Na_2CO_3$ pH9.6 (anti-LRP1 (1:1000, Abcam, ab92544), anti-Transferrin Receptor (OKT-9) (1:1000; eBioscience, 14-0719-82) or anti-PDGFRβ (1:500, Abcam, ab32570)) overnight at 4°C. Plates were washed and blocked for 1 hour in 1% Hammarsten grade casein (Alfa Aesar) with 0.1% Tween-20 in PBS. Lysates were quantified by BCA assay, diluted in blocking buffer, equal amounts added to the plate and incubated overnight. Plates were washed, incubated with streptavidin-HRP (1:1000, Abcam, ab7403) for 1 hour and developed with TMB substrate (BD Biosciences). The reaction was stopped with 1M sulphuric acid and absorbance read at 450 nm.

***NMR Analysis. i) Protein expression and purification.*** The pGEX-4T1 vector containing the N-terminus GST-tagged intracellular domain of LRP 1 (residues 4445 to 4544, a kind gift from Petra May, University of Freiburg, Germany)(10) was transformed into E. coli BL21 competent cells. Cultures were grown at 37°C in $^{15}N$-$NH_4Cl$, or $^{15}N$- $NH_4Cl$- $^{13}C$ glucose minimal medium until an $OD_{600}$ of 0.9 was reached. Protein over-expression was induced with the addition of 0.5M IPTG, and incubated at 18°C for 12 hours. LRP1-ICD was purified using GST affinity chromatography, followed by cleavage from the GST tag using 12.5 units of thrombin. Protein was then concentrated and purified using a gel filtration HiLoad 16/600 Superdex 75 pg column in a buffer containing 25 mM sodium phosphate pH 6.5, and 150 mM NaCl. Chromatography was performed on an Akta FPLC purification device. Following purification, the protein was evaluated using mass spectrometry to validate its size, followed by concentration for CD and NMR analysis. **ii)** ***NMR spectroscopy.*** LRP1-ICD protein was concentrated to 90 μM for Tβ4 titration analysis, or 300 μM for triple resonance backbone experiments. $^{1}H$-$^{15}N$ SOFAST HMQC experiments (11) were used to monitor titrations by increasing the ratio of synthetic Tβ4 peptide (a kind gift from RegeneRx Biopharmaceuticals)

to LRP1-ICD protein from 0:1 to 10:1, a point at which no further changes were observed in the NMR spectra. To eliminate the possibility of a buffer effect, Tβ4 was desalted and dialyzed against 25 mM sodium phosphate pH 6.5 and 150 mM NaCl, i.e. the identical buffer used for LRP1-ICD. Backbone $^{1}H$, $^{15}N$, $^{13}C'$, $^{13}C\alpha$ and $^{13}C\beta$ assignments for LRP1-ICD were obtained from standard triple resonance experiments (CBCACONH, HNCACB, HNCO, and HNCACO(12)). NMR experiments were carried out at 298 K on Bruker 500 or 900 MHz spectrometers equipped with cryogenic probes (Bruker). Data was processed using NMRPipe(13, 14) and analyzed using CCPN.

***Proteomic analysis of LRP1-PDGFRβ complexes.*** Abdominal aortas of n=3 $T\beta4^{+/Y}$ mice were harvested in and lysed in RIPA buffer (50 mM Tris-HCl pH7.5, 100 mM NaCl, 1% NP-40, 0.1% SDS, 0.5% sodium deoxycholate, PhosSTOP and cOmplete protease inhibitor cocktail (Roche)) using a Qiagen TissueLyser LT. MOVAS-1 cells were grown to 80% confluency in 10cm dishes and then switched to medium without FBS overnight. Cells were stimulated with 20 ng/ml PDGF-BB on ice for 1 hour. The cells were then shifted to 37°C for 10 min or kept on ice (0 min control). Cells were washed twice with cold PBS before addition of 5 mM Disuccinimidyl Sulfoxide (DSSO) for 30 min on ice to crosslink protein complexes. The crosslinking reaction was quenched for 15 min with 20 mM Tris-HCl, pH8. Cells were washed twice with cold PBS and harvested by scraping into PBS. After centrifugation (400x g, 10min, 4°C), cell pellets were resuspended in lysis buffer (50 mM Tris, 150 mM NaCl, 1% Nonidet P-40, PhosSTOP and cOmplete protease inhibitor cocktail (Roche)). Cell and aorta lysates were quantified by BCA assay (Pierce) and equal protein amounts were used for immunoprecipitation (1mg for aorta lysates, 90-150 μg for MOVAS-1 lysates). Lysates were precleared with 50 μl of Protein A Dynabeads (Life Technologies) and 10 μg rabbit IgG (Life Technologies, 31887) per sample. Protein complexes were precipitated overnight at 4°C with 50 μl of fresh Protein A Dynabeads and 10 μg of anti-N-terminal LRP1 antibody (R2629(15)) (a kind gift from Dudley Strickland, University of Maryland) or 2.5 μg anti-PDGFRβ antibody (Y92) (Abcam, ab32570). Beads were washed once with lysis buffer, twice with wash buffer (50 mM Tris-Hcl, pH7.5, 150 mM NaCl) and once with ultrapure water. 20% of beads were used for Western blotting to confirm immunoprecipitation success and the remaining beads were submitted for analysis by mass spectrometry. On-bead digestion was performed, as described(16), with denaturation in 8M Urea for 30 minutes, followed by trypsin digestion overnight. After clean-up by solid phase extraction, peptides were resuspended in 5% formic acid, 5% dimethyl sulfoxide and trapped on an Acclaim PepMapTM 100 pre-column (C18, 100µm i.d. x 2cm, nanoViper, 100 Å, Thermo Fisher Scientific) using solvent A (0.1% Formic Acid in water) at a pressure of 60 bar and separated on an Ultimate 3000 UHPLC system (Thermo Fischer Scientific) coupled to a QExactive mass spectrometer (Thermo Fischer Scientific). The peptides were separated on a PepMap™ analytical column (C18, 75µm i.d.x 2µm, 50cm, nanoViper, 100 Å; Thermo Fischer Scientific) and then electrosprayed directly into an QExactive mass spectrometer (Thermo Fischer Scientific) through an EASY-Spray nano-electrospray ion source (Thermo Fischer Scientific) using a linear gradient (length: 60 minutes, 5% to 35% solvent B (0.1% formic acid in acetonitrile), flow rate: 250nL/min). Raw data were acquired on the mass spectrometer in a data-dependent mode (DDA). Full scan MS spectra were acquired in the Orbitrap (scan range 380-1800 m/z, resolution 70000, AGC target 3e6, maximum injection time 100ms). After the MS scans, the 15 most intense peaks were selected for HCD fragmentation at 28% of normalised collision energy. HCD spectra were also acquired in the Orbitrap (resolution 17500, AGC target 1e5, maximum injection time 128 ms) with first fixed mass at 100 m/z. The raw data files generated were processed using MaxQuant (Version 1.6.10.43)(17), integrated with the Andromeda search engine. For protein group identification, peak lists were searched against the mouse database (UPR_mus musculus_08/20, version 08/20) as well as list of common contaminants by Andromeda.

Trypsin with a maximum number of missed cleavages of 2 was chosen. Oxidation (M) and Deamidation (N,Q) were used as variable modifications while Carbamidomethylation (C) was set as a fixed modification. Protein and PSM false discovery rate (FDR) were set at 0.01. Match between runs was applied.

***RNA isolation and gene expression profile by quantitative real time (qRT-PCR).*** Total RNA was isolated from the aortic arch or descending aorta of adult mice using the RNeasy Fibrous Tissue Mini kit (Qiagen), according to the manufacturer's instructions. cDNA was prepared using a reverse transcription kit (Applied Biosystems). qRT-PCR was performed on a ViiA 7 instrument (Life technologies) using fast SYBR Green (Applied Biosystems). Data were normalised to β-actin expression (endogenous control with the most stable expression selected from a panel of 6 housekeeping genes). Fold-changes were determined by the $2^{-\Delta\Delta}$CT method(18).

***Primers for qRT-PCR (5'-3').***
*Actb* F: GGCTGTATTCCCCTCCATCG; R: CCAGTTGGTAACAATGCCATGT;
*Sm22α* F: CAACAAGGGTCCATCCTACGG; R: ATCTGGGCGGGCCTACATCA;
*Tropomyosin IV* F: CGCAAGTATGAGGAGGTTGC; R: AGTTTCAGATACCTCCGCCCT;
*Tmsb4x* F: ATGTCTGACAAACCCGATATGGC; R: CCAGCTTGCTTCTCTTGTTCA;
*Ccnd1* F: 5'GTT CAT TTC CAA CCC ACC CTC; R: AGA AAG TGC GTT GTG CGG TAG;
*Pdgfb* F: TGT TCC AGA TCT CGC GGA AC; R: GCG GCC ACA CCA GGA AG.

***RNAscope® single molecule RNA in situ hybridisation***. The RNAscope® Multiplex Fluorescent v2 Assay (AcDBio) was used to visualize and quantify single molecule mRNA targets per cell on cryosections. Fixed aortic sections were pre-treated with RNAscope Pre-treatment kit to unmask target RNA and permeabilise. Protocol was performed according to the manufacturer's instructions, using the RNAscope® Probe - Mm-Tmsb4x-C2 472851-C2, followed by sequential amplification of signal using Opal detection reagents (Akoya Biosciences). Sections were imaged using an Olympus FV1000 confocal microscope.

***Proliferation assays.*** After trypsinization, 1000 cells/well were plated in DMEM; 10% FBS into 96-well plates. After overnight serum starvation, medium was replaced with serum-free DMEM containing PDGF-BB (2-50ng/ml). At the harvest time point, plates were washed frozen and processed for DNA-based assay, as described(19), and background subtracted using a plate which had been incubated with medium but no cells.

***Migration assays.*** Primary aortic VSMCs were cultured as a confluent monolayer in 12-well plates. After overnight serum starvation, scratch wounds were introduced with a P200 tip. After washing, medium was replaced with serum-free DMEM containing PDGF-BB (2-50ng/ml) and 100 µM hydroxyurea (Sigma). Wells were imaged at t0, and every 24 hours thereafter, using a Zeiss Axio Imager microscope. Wounds were measured and expressed as % relative to t0.

***Statistics.*** Randomisation of animals to treatment or genotype groups was introduced at the time of mini pump implantation (aneurysm) or harvest (baseline). Thereafter, tissues were processed and analysed by an independent observer whilst blinded to treatment or genotype. Statistical analyses were performed with GraphPad Prism Software. For the quantitative comparison of two groups, two-tailed unpaired Student's t-test was used to determine any significant differences, after assessing the requirements for a t-test using a Shapiro-Wilk test for normality and an F-Test to compare variances. Alternatively, a Mann-Whitney non-parametric test was used. For comparison of three groups or more, a one-way ANOVA with Tukey's post-hoc test was used. For analyses involving two independent variables, a two-way ANOVA with Bonferroni, Holm-Sidak or Dunnett's post-hoc test was used, after Shapiro-Wilk

test for normality. Significance is indicated in the figures, as follows: *: p≤0.05; **: p≤0.01; ***: p≤0.001; ****: p≤0.0001.

***Study approval.*** The Oxford Abdominal Aortic Aneurysm (OxAAA) study was approved by the Oxford regional ethics committee (Ethics Reference: 13/SC/0250). All procedures involving the use and care of animals were performed in accordance with the Animals (Scientific Procedures) Act 1986 (Home Office, United Kingdom) and approved by the University of Oxford or University College London Animal Welfare and Ethical Review Boards.

## References


1. Rossdeutsch A, Smart N, Dube KN, Turner M, and Riley PR. Essential role for thymosin beta4 in regulating vascular smooth muscle cell development and vessel wall stability. *CircRes.* 2012;111(4):e89-102.
2. Smart N, Risebro CA, Melville AAD, Moses KA, Schwartz RJ, Chien KR, et al. Thymosin b4 induces adult epicardial progenitor mobilization and neovascularization. *Nature.* 2007;445(7124):177-82.
3. Wirth A, Benyo Z, Lukasova M, Leutgeb B, Wettschureck N, Gorbey S, et al. G12-G13-LARG-mediated signaling in vascular smooth muscle is required for salt-induced hypertension. *NatMed.* 2008;14(1):64-8.
4. Boucher P, Gotthardt M, Li WP, Anderson RG, and Herz J. LRP: role in vascular wall integrity and protection from atherosclerosis. *Science.* 2003;300(5617):329-32.
5. Daugherty A, Tall AR, Daemen M, Falk E, Fisher EA, Garcia-Cardena G, et al. Recommendation on Design, Execution, and Reporting of Animal Atherosclerosis Studies: A Scientific Statement From the American Heart Association. *Arterioscler Thromb Vasc Biol.* 2017;37(9):e131-e57.
6. Smart N, Riegler J, Turtle CW, Lygate CA, McAndrew DJ, Gehmlich K, et al. Aberrant developmental titin splicing and dysregulated sarcomere length in Thymosin beta4 knockout mice. *Journal of molecular and cellular cardiology.* 2017;102:94-107.
7. Cherepanova OA, Gomez D, Shankman LS, Swiatlowska P, Williams J, Sarmento OF, et al. Activation of the pluripotency factor OCT4 in smooth muscle cells is atheroprotective. *Nature medicine.* 2016;22(6):657-65.
8. Banerjee I, Zhang J, Moore-Morris T, Lange S, Shen T, Dalton ND, et al. Thymosin beta 4 is dispensable for murine cardiac development and function. *CircRes.* 2012;110(3):456-64.
9. Bolte S, and Cordelieres FP. A guided tour into subcellular colocalization analysis in light microscopy. *Journal of microscopy.* 2006;224(Pt 3):213-32.
10. Zurhove K, Nakajima C, Herz J, Bock HH, and May P. Gamma-secretase limits the inflammatory response through the processing of LRP1. *SciSignal.* 2008;1(47):ra15.
11. Schanda P, Kupce E, and Brutscher B. SOFAST-HMQC experiments for recording two-dimensional heteronuclear correlation spectra of proteins within a few seconds. *J Biomol NMR.* 2005;33(4):199-211.
12. Sattler M, Schleucher J, and Griesinger C. Heteronuclear multidimensional NMR experiments for the structure determination of proteins in solution employing pulsed field gradients. *Progress in Nuclear Magnetic Resonance Spectroscopy.* 1999;34(2):93-158.
13. Delaglio F, Grzesiek S, Vuister GW, Zhu G, Pfeifer J, and Bax A. NMRPipe: a multidimensional spectral processing system based on UNIX pipes. *J Biomol NMR.* 1995;6(3):277-93.
14. Skinner SP, Fogh RH, Boucher W, Ragan TJ, Mureddu LG, and Vuister GW. CcpNmr AnalysisAssign: a flexible platform for integrated NMR analysis. *J Biomol NMR.* 2016;66(2):111-24.

15. Mikhailenko I, Battey FD, Migliorini M, Ruiz JF, Argraves K, Moayeri M, et al. Recognition of α2-Macroglobulin by the Low Density Lipoprotein Receptor-related Protein Requires the Cooperation of Two Ligand Binding Cluster Regions. *Journal of Biological Chemistry.* 2001;276(42):39484-91.
16. Wiśniewski JR, Zougman A, Nagaraj N, and Mann M. Universal sample preparation method for proteome analysis. *Nature Methods.* 2009;6(5):359-62.
17. Cox J, and Mann M. MaxQuant enables high peptide identification rates, individualized p.p.b.-range mass accuracies and proteome-wide protein quantification. *Nature Biotechnology.* 2008;26(12):1367-72.
18. Livak KJ, and Schmittgen TD. Analysis of relative gene expression data using real-time quantitative PCR and the 2(-Delta Delta C(T)) Method. *Methods (San Diego, Calif).* 2001;25(4):402-8.
19. Rago R, Mitchen J, and Wilding G. DNA fluorometric assay in 96-well tissue culture plates using Hoechst 33258 after cell lysis by freezing in distilled water. *Analytical biochemistry.* 1990;191(1):31-4.

# Full unedited blots for Figure 1H

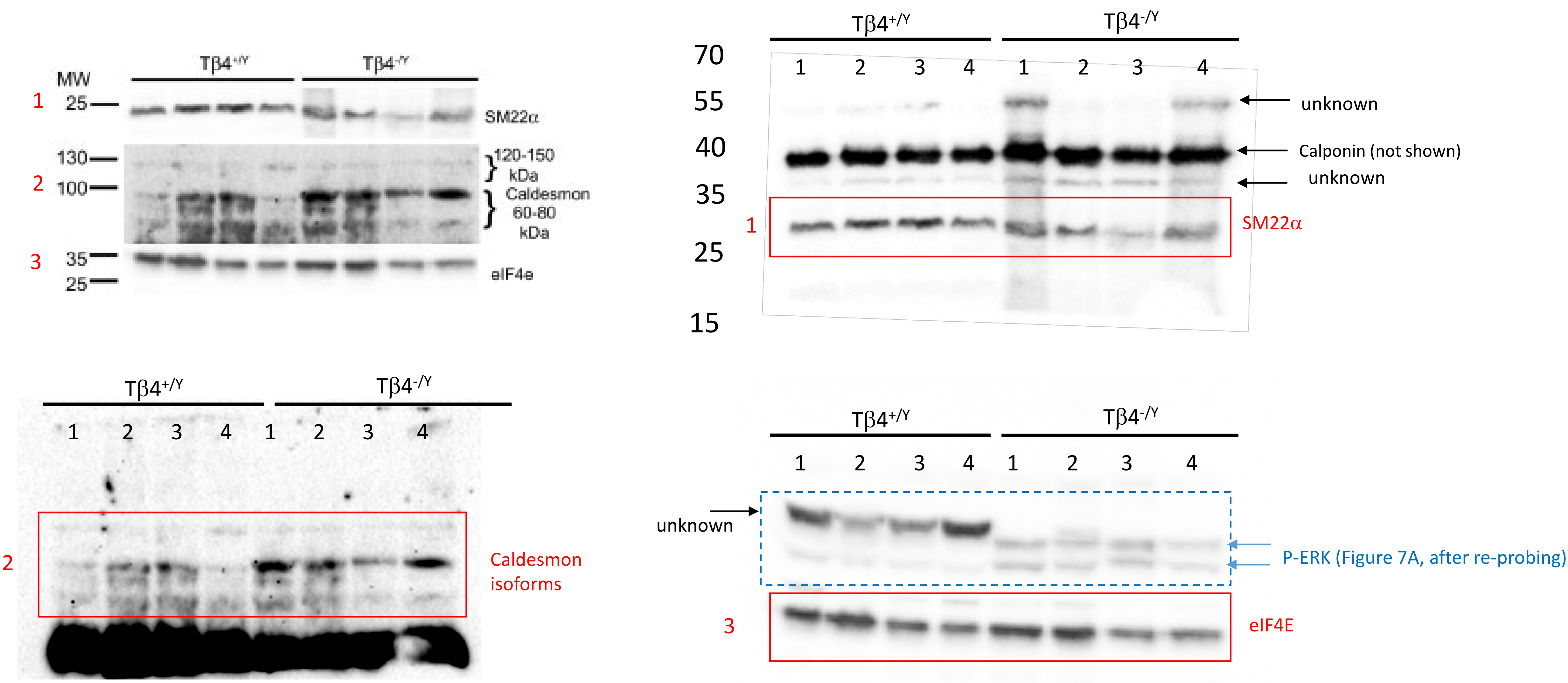

Full unedited blots for Figure 7A

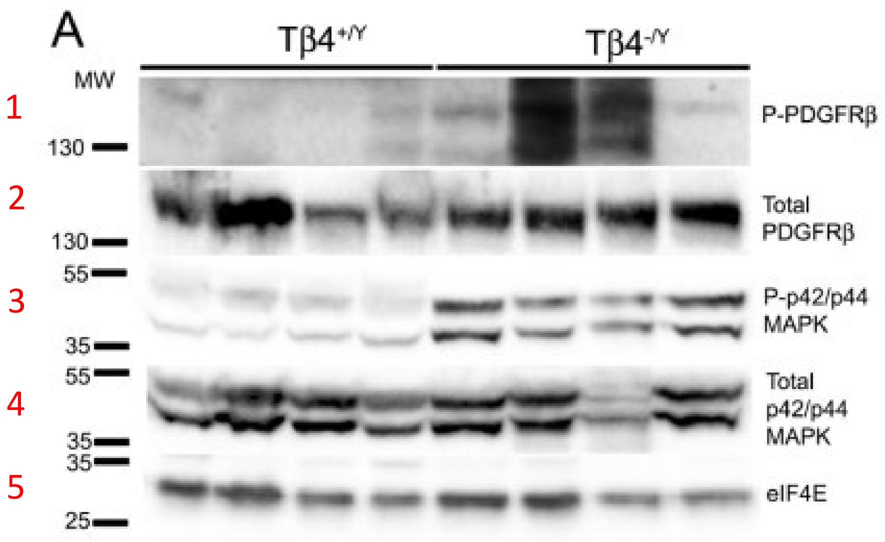


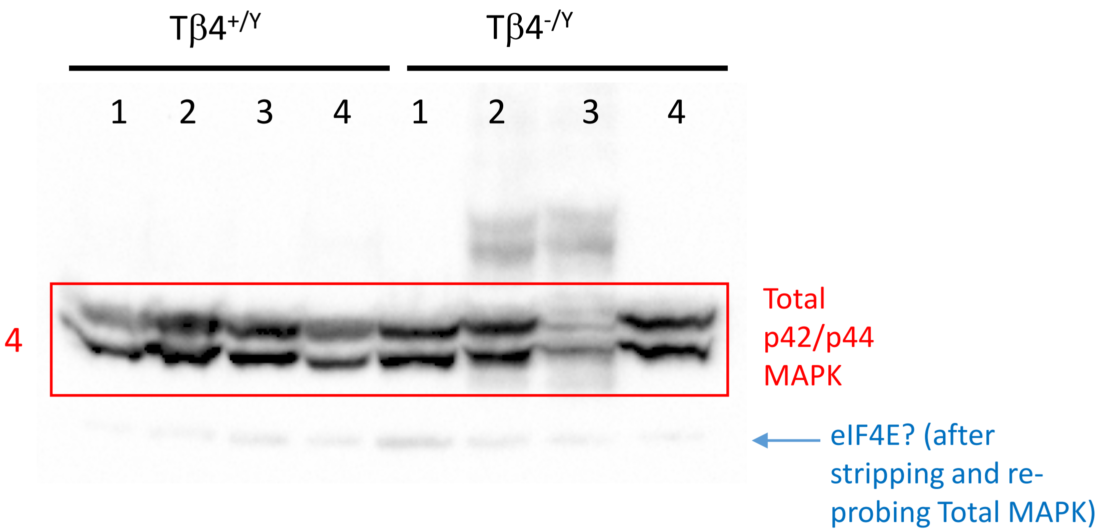


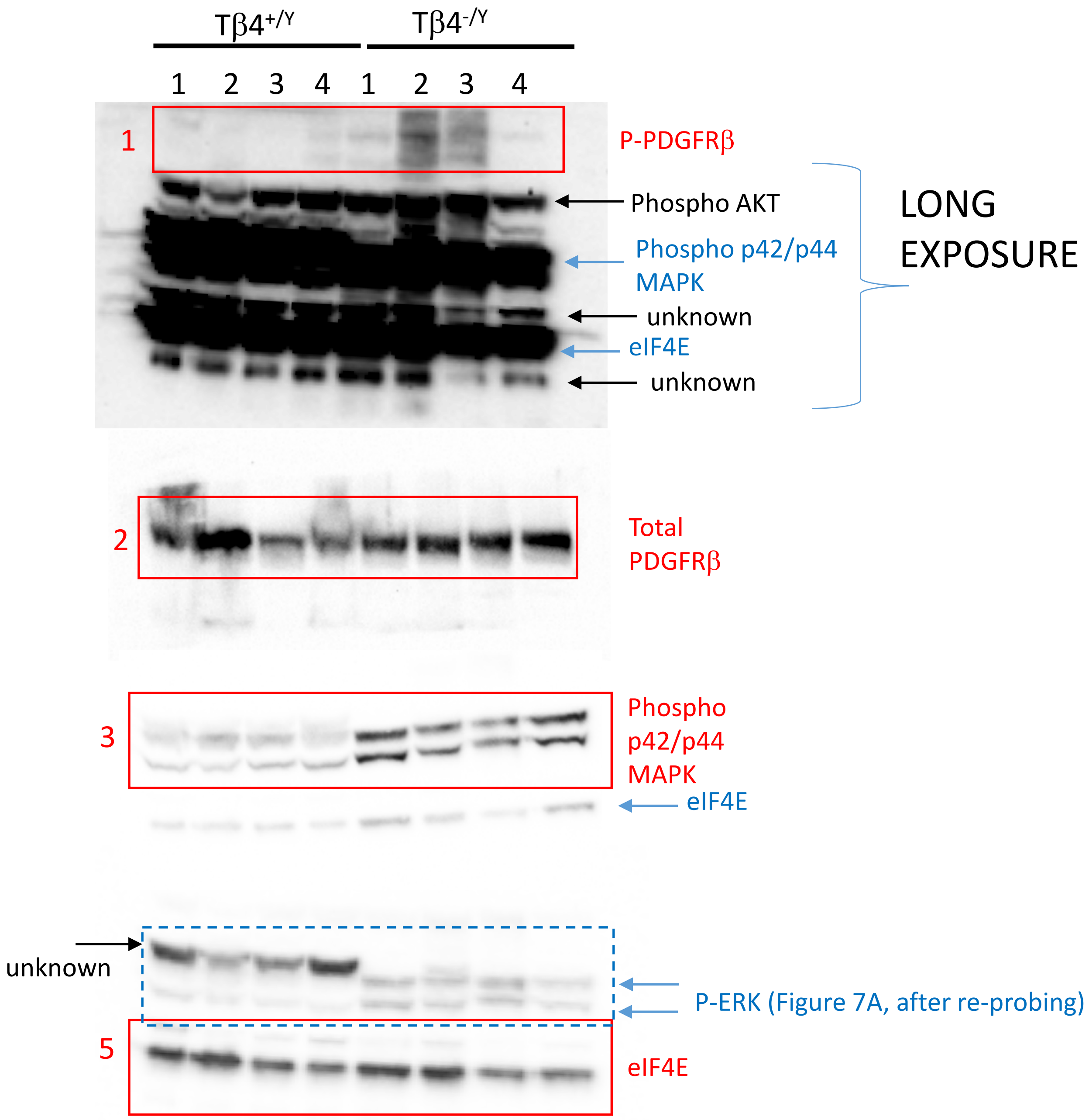

# Full unedited blots for Figure 7C

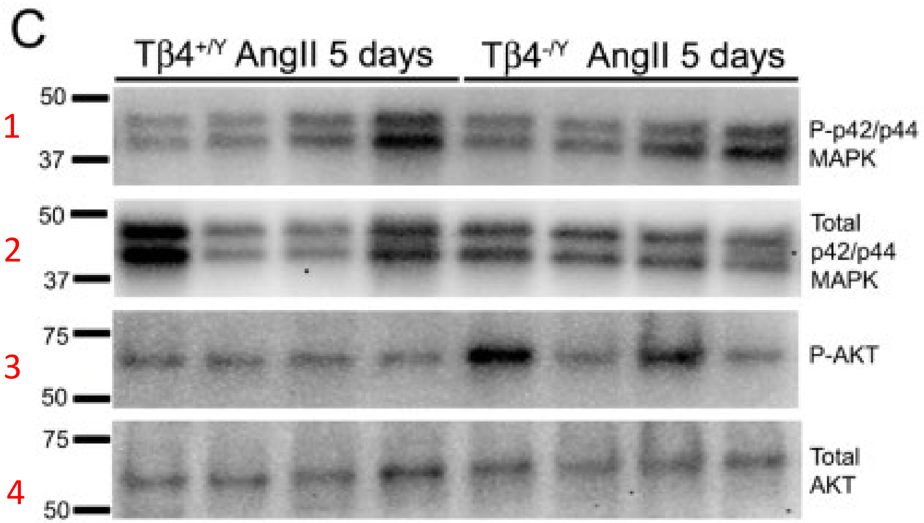


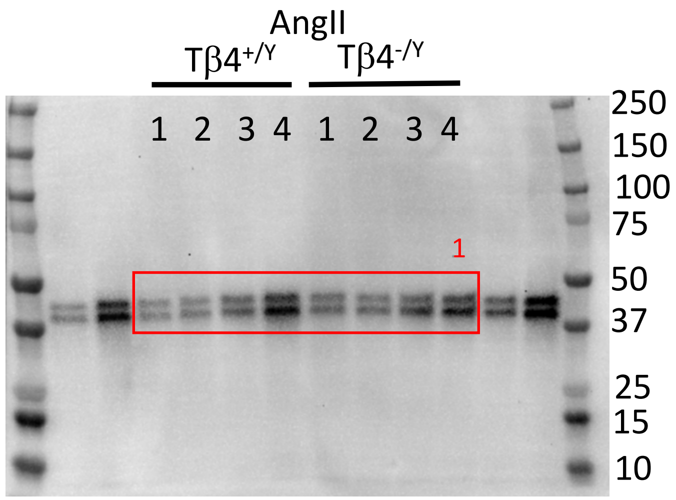


Phospho p42/p44 MAPK

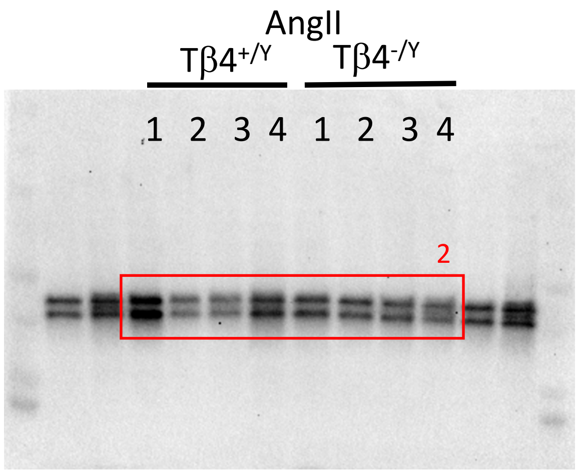


Total p42/p44 MAPK

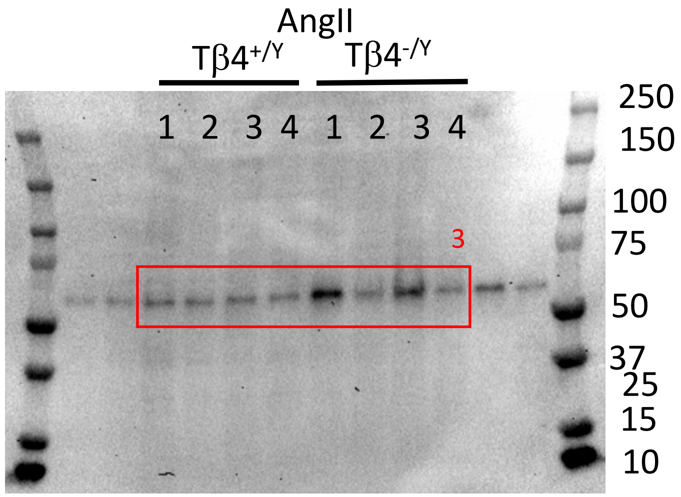


Phospho AKT

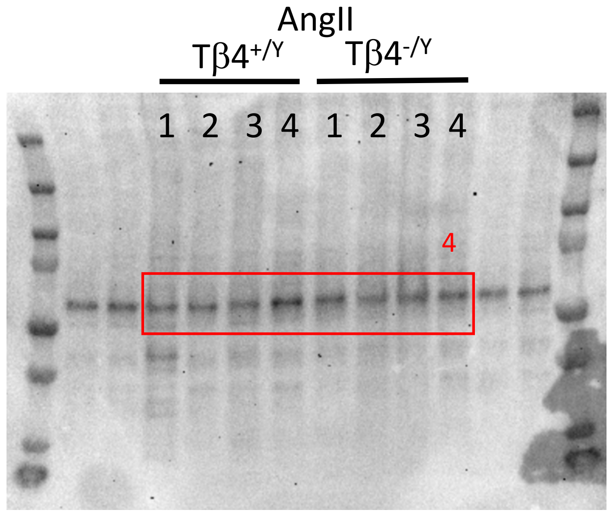


Total AKT

Full unedited blots for Figure 9A

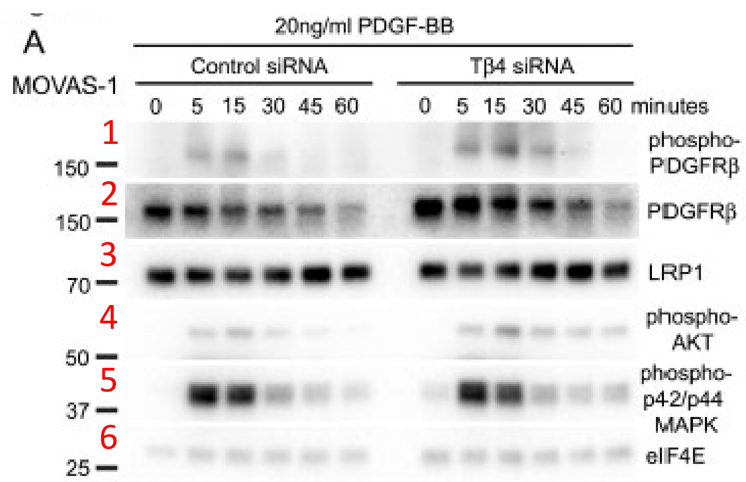


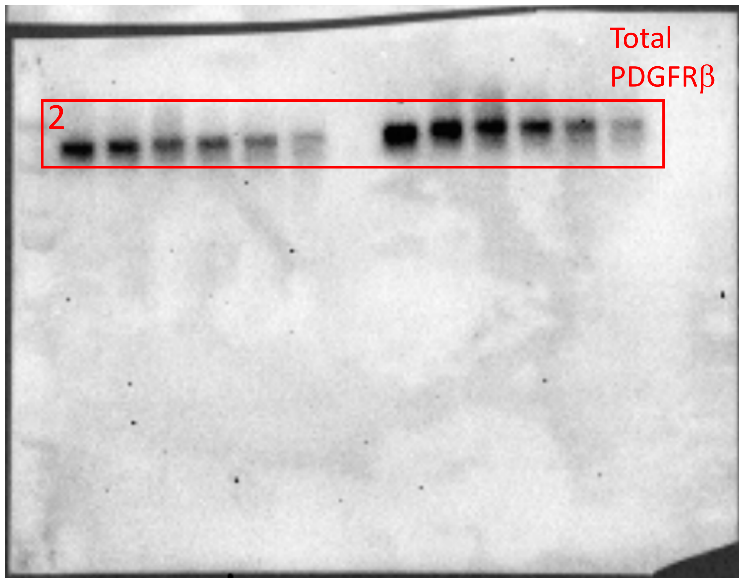


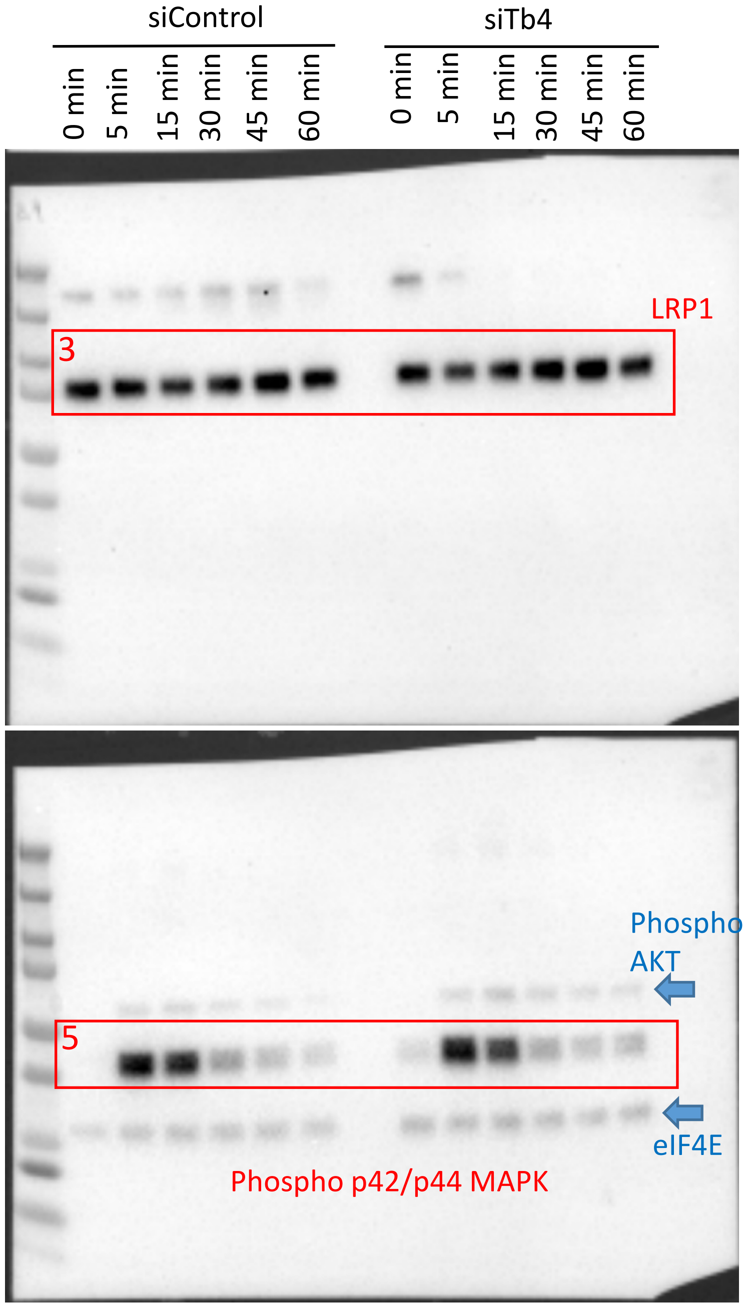


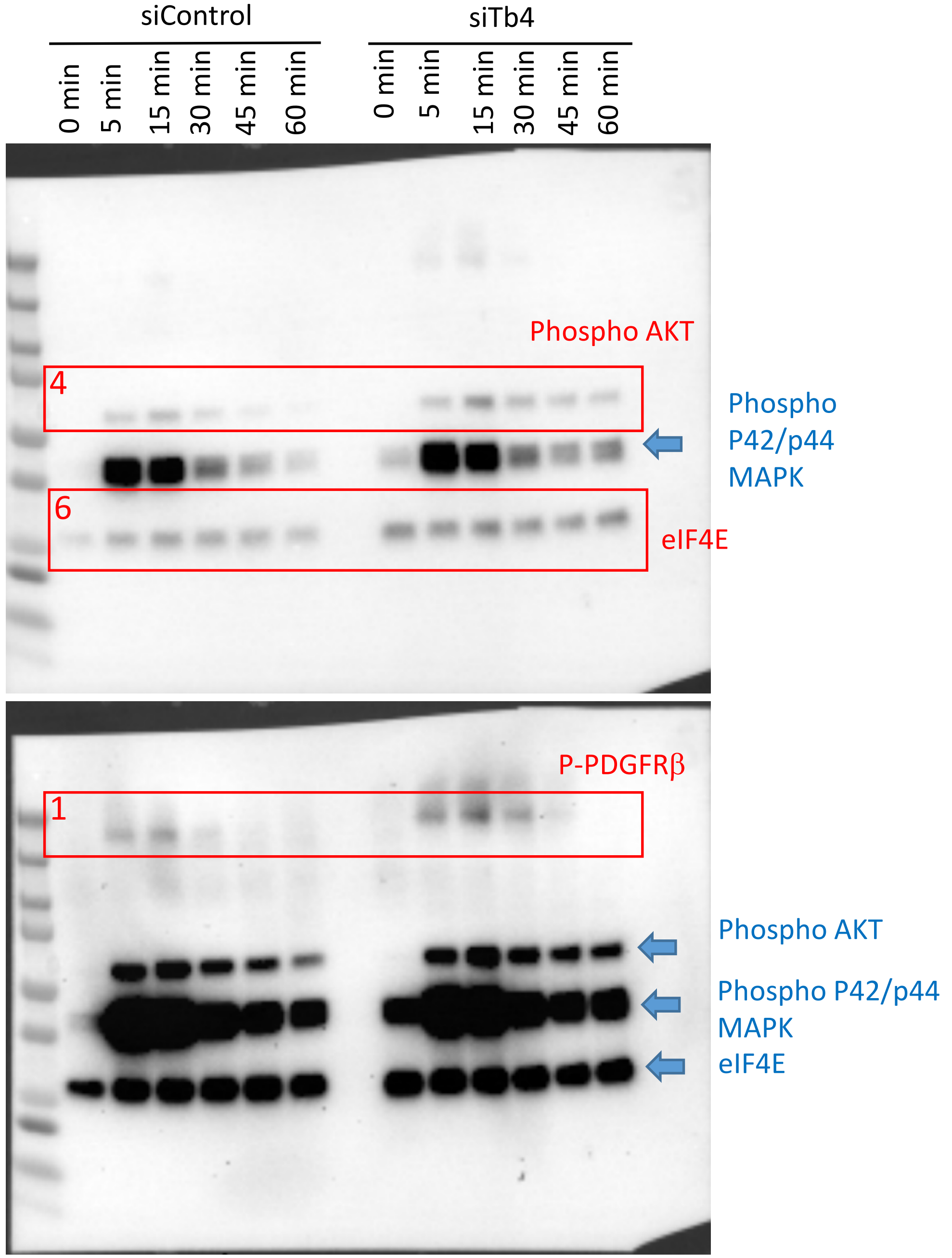

Full unedited blots for Figure 10G

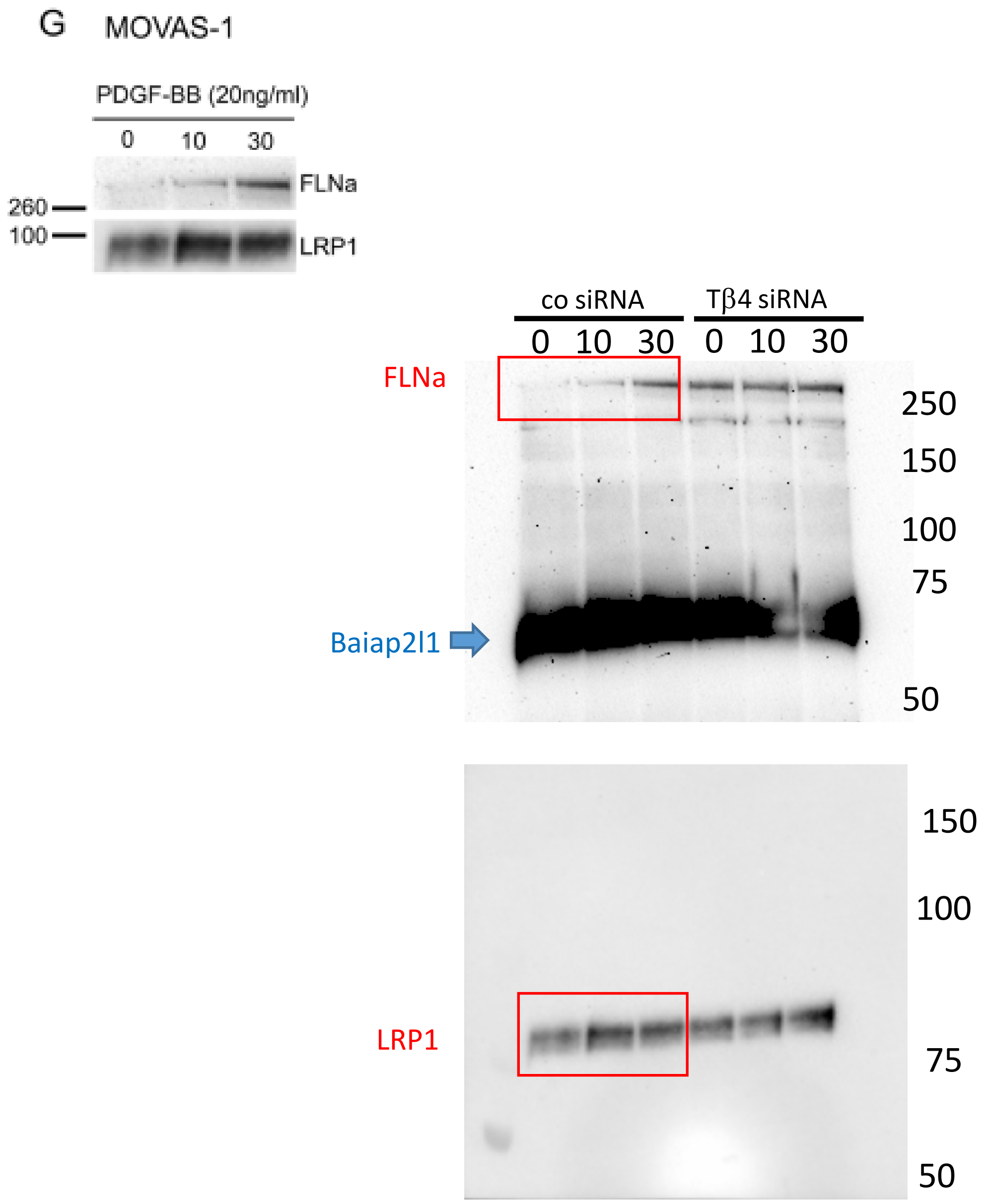


Full unedited blots for Figure 10H

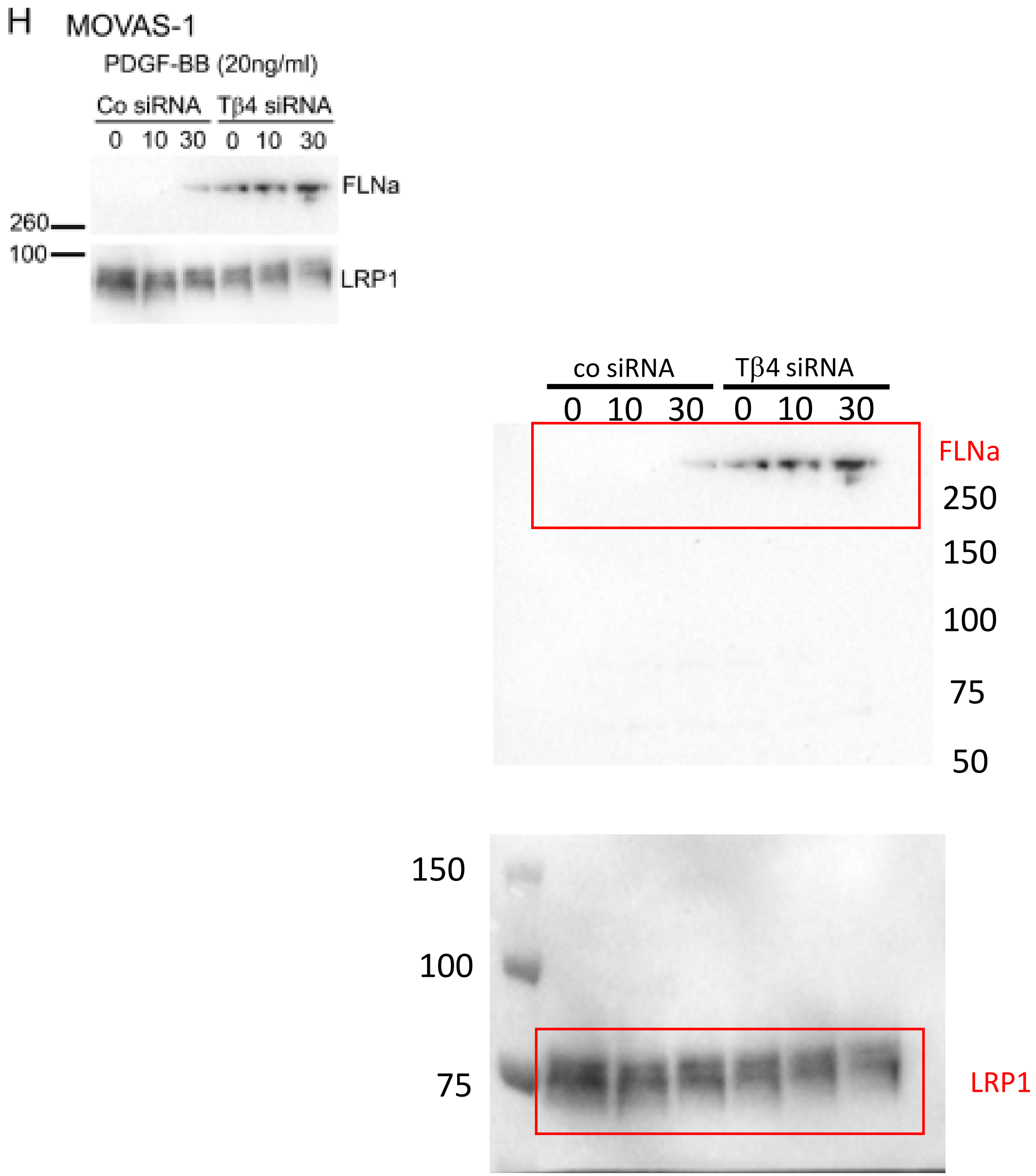

# Full unedited blots for Figure 11A

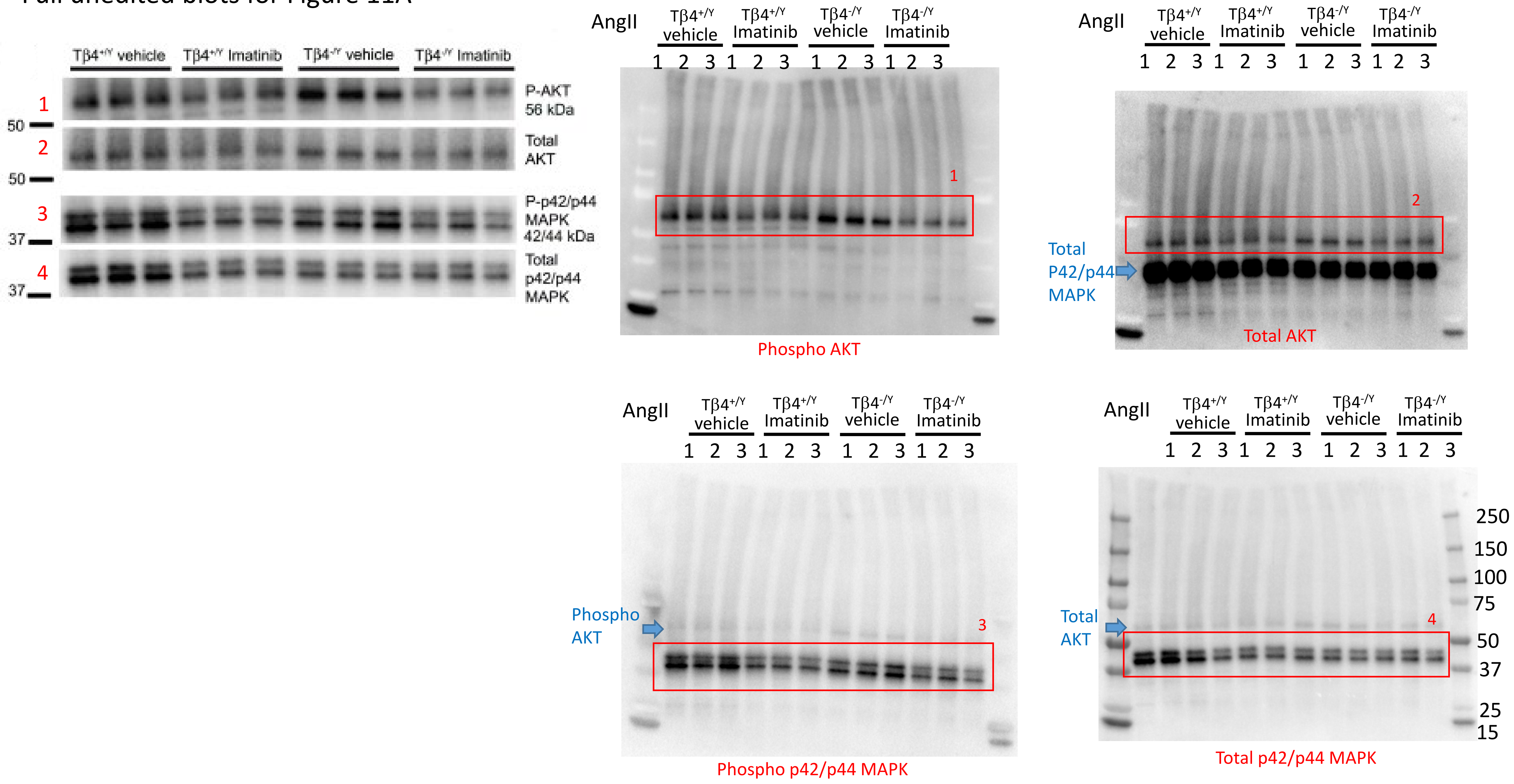

# Full unedited blots for Supplemental Figure 9A

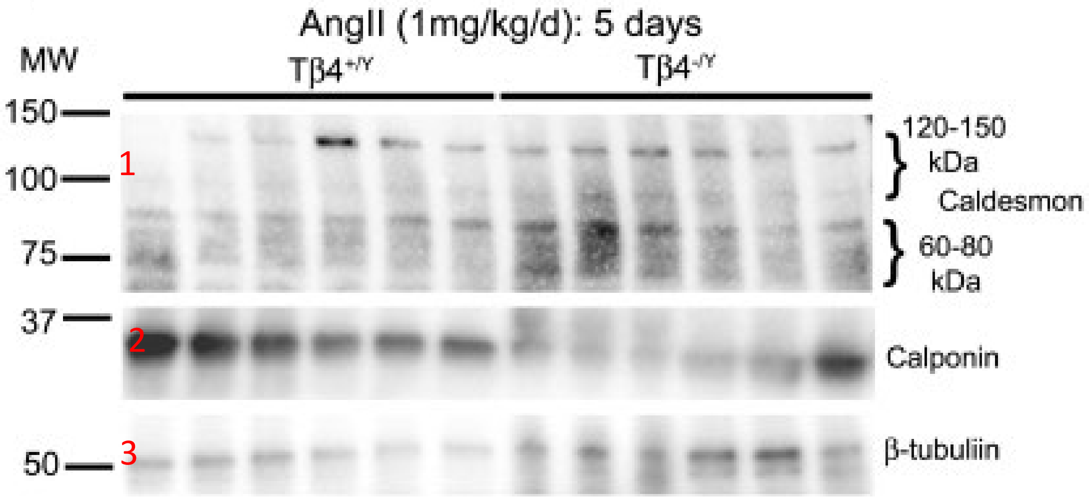


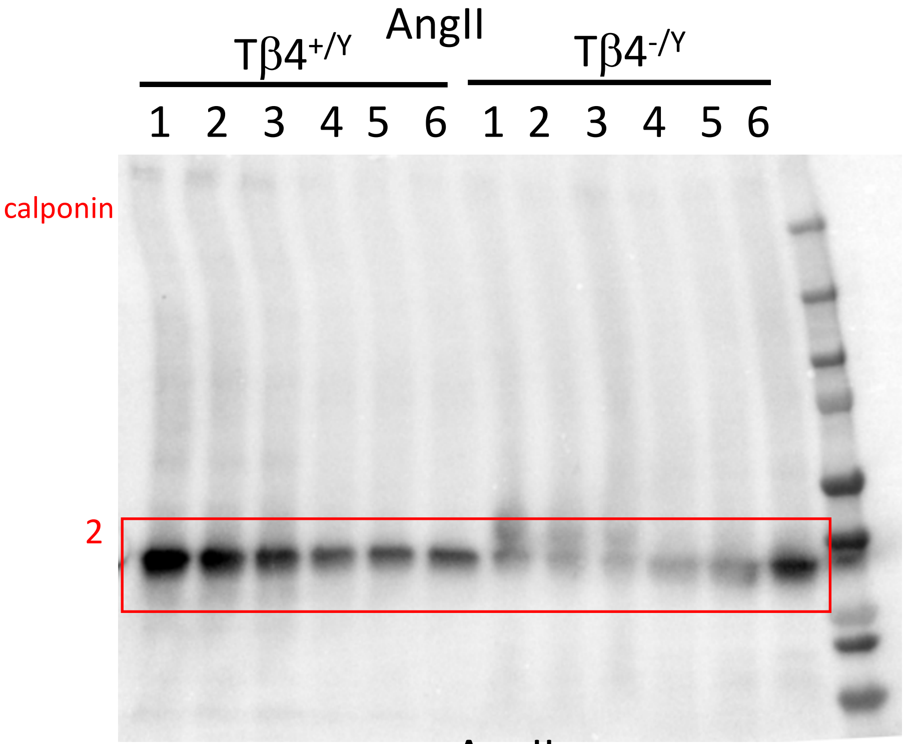


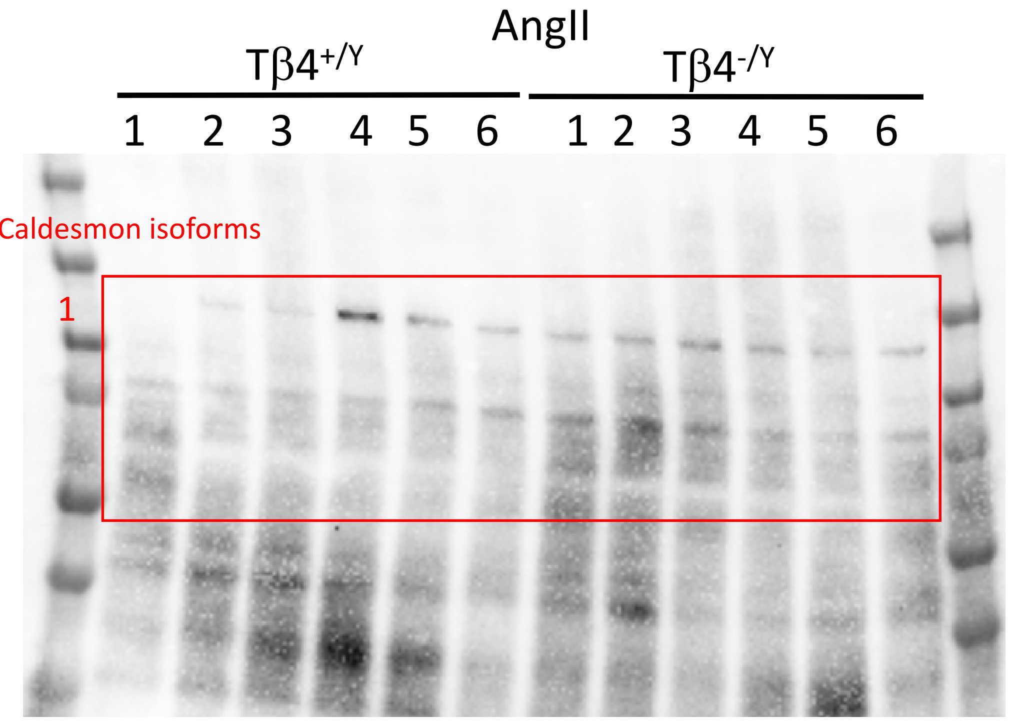


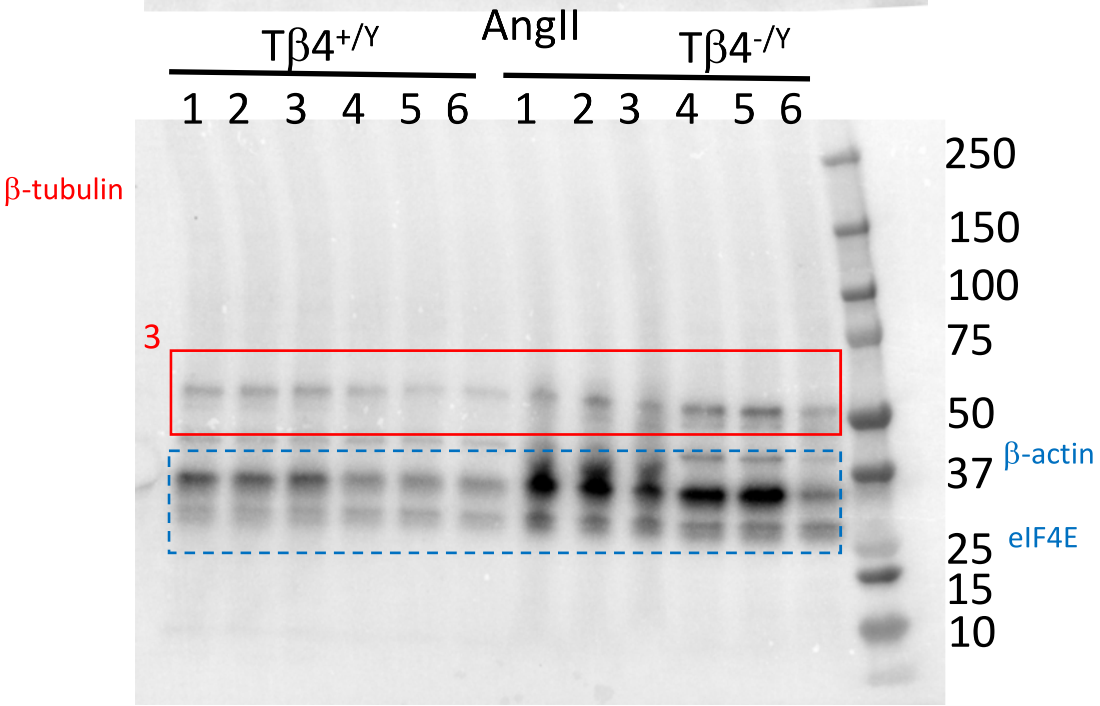


Additional endogenous controls were used to validate loading. For densitometry, we normalised against β-tubulin or eIF4E, which correlated well with one another. β-actin showed some degradation (common for β-actin) and is potentially unreliable due to the role of TB4 in binding actin.

# Full unedited blots for Supplemental Figure 13C

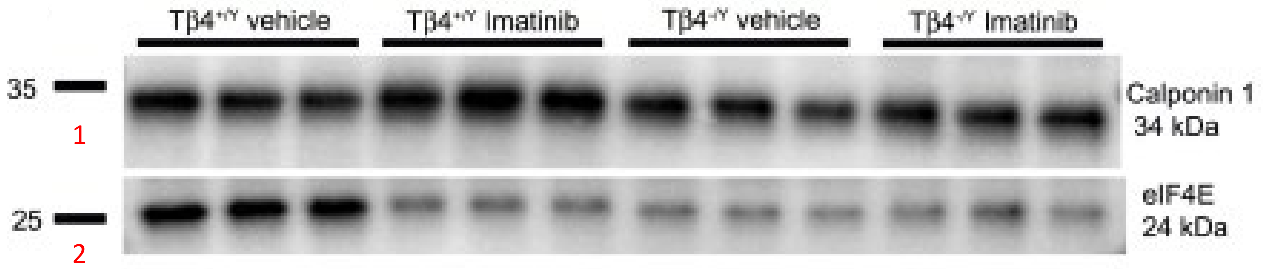


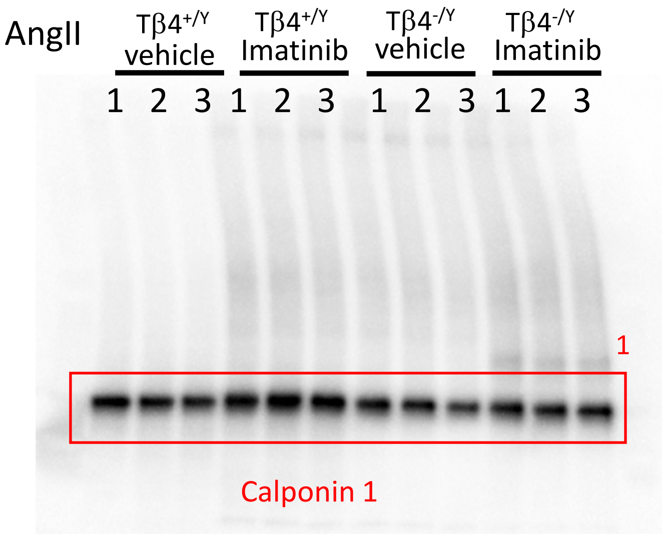


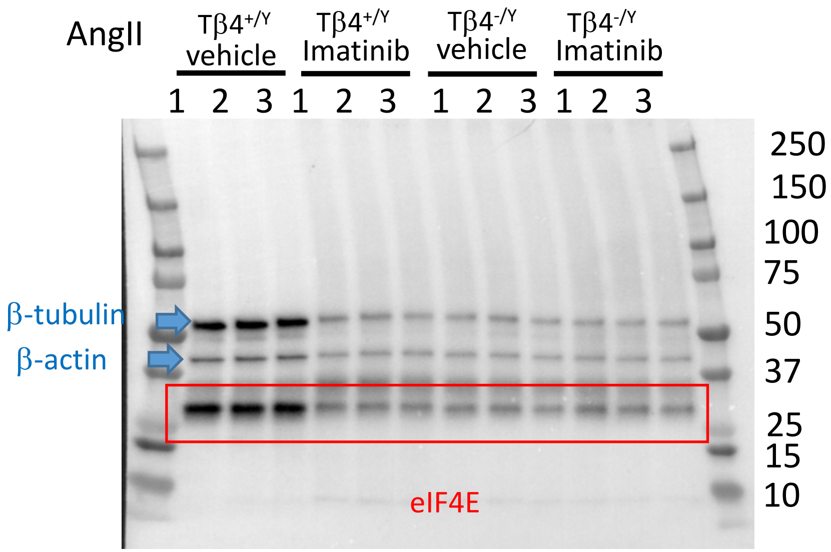

Supplement: Supplemental data [file jci-131-127884-s023.pdf]
